# Discovery of VU6052254: A Novel, Potent M_1_ Positive Allosteric Modulator

**DOI:** 10.1021/acschemneuro.5c00825

**Published:** 2025-11-26

**Authors:** Julie L. Engers, Joseph D. Bungard, Christopher C. Presley, Irene Zagol-Ikapitte, Katherine J. Watson, Sichen Chang, Colin O’Carroll, P. Markus Dey, Ethan S. Burstein, Jonathan W. Dickerson, Analisa Thompson Gray, Michael Bubser, Jerri M. Rook, Hyekyung P. Cho, Valerie M. Kramlinger, Olivier Boutaud, Carrie K. Jones, P. Jeffrey Conn, Darren W. Engers, Craig W. Lindsley

**Affiliations:** † Warren Center for Neuroscience Drug Discovery, 5718 Vanderbilt University, Nashville, Tennessee 37232, United States; ‡ Department of Pharmacology, Vanderbilt University School of Medicine, Nashville, Tennessee 37232, United States; § Department of Chemistry, Vanderbilt University, Nashville, Tennessee 37232, United States; ∥ Vanderbilt Institute of Chemical Biology, Vanderbilt University, Nashville, Tennessee 37232, United States; ⊥ Acadia Pharmaceuticals Inc., San Diego, California 92130, United States; # Vanderbilt Institute for Therapeutic Advances, Vanderbilt University, Nashville, Tennessee 37232, United States

**Keywords:** muscarinic acetylcholine receptor subtype 1 (M_1_), positive allosteric modulator (PAM), tertiary alcohol, cognition, metabolism

## Abstract

We recently disclosed VU0467319, a muscarinic acetylcholine receptor subtype 1 (M_1_) Positive Allosteric Modulator (PAM) clinical candidate that had successfully completed a Phase I Single Ascending Dose (SAD) clinical trial, but the identification of an inactive metabolite constituting a major portion of the total plasma AUC detracted from the molecules’ pharmacokinetic profile and contributed to clinical development discontinuation. Attempts to block metabolism with the incorporation of deuterium atoms proved successful *in vitro* and *in vivo* at low exposures; however, in high-dose nonclinical toxicology studies, the degree of oxidative metabolism and metabolite accumulation was comparable to that of the proteo-congener. Here, we describe a second-generation back-up effort based on the VU0467319 scaffold to discover VU6052254. Strategic placement of a tertiary hydroxyl moiety afforded VU6052254, a potent M_1_ PAM (EC_50_ = 59 nM, 79% ACh max), with high CNS exposure (rat K_p_ = 1.07; K_p,uu_ = 1.27; P-gp ER = 1.97, P_app_ = 23 × 10^–6^ cm/s), reduced metabolism across species, excellent pharmacodynamic responses (MED in rat NOR = 1 mg/kg PO; MED in rat CFC = 0.3 mg/kg PO), excellent multispecies PK (Cl_p_ *s* < 10 mL/min/kg, %*F* > 65), and favorable human PK and dose projections. Based on these beneficial attributes, VU6052254 was nominated for further nonclinical development. However, possible CYP_450_ induction liability as well as uncertain projected margins for human efficacy at those systemic concentrations where dose/exposure-related clinical and anatomic pathology kidney findings were observed in a 14-day exploratory toxicity study in male rats, precluded further development.

## Graphical abstract

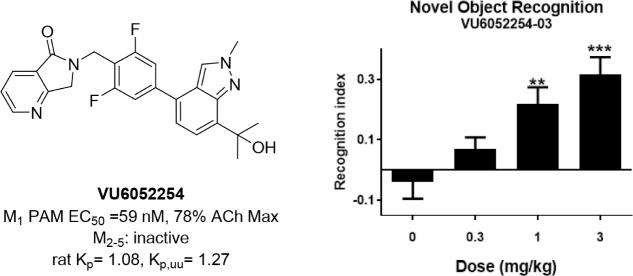

## Introduction

The Warren Center for Neuroscience Drug Discovery (WCNDD), and many other laboratories, have been actively developing small molecules that selectively activate the M_1_ muscarinic acetylcholine receptor (both allosteric agonists and positive allosteric modulators (PAMs)) for almost two decades.^1–22^ M_1_ PAMs proved to be an effective pharmacological approach for selectively activating M_1_ receptors and avoiding activation of M_2–5_ as well avoiding on-target excitotoxicity observed with agonists and potent ago-PAMs in preclinical species.^5–22^ Over the years, we disclosed many essential M_1_ PAM *in vivo* tool compounds, with minimal M_1_ receptor direct agonism, and insights into both their pro-cognitive efficacy and excitotoxicity.^14,16–18^ Recently, these efforts culminated in the discovery and development of VU0467319 (**1**), an M_1_ PAM clinical candidate that successfully completed a Phase I SAD clinical trial (Figure 1) pro-cognitive efficacy and without any observed cholinergic toxicity.^22–24^

**Figure 1:**
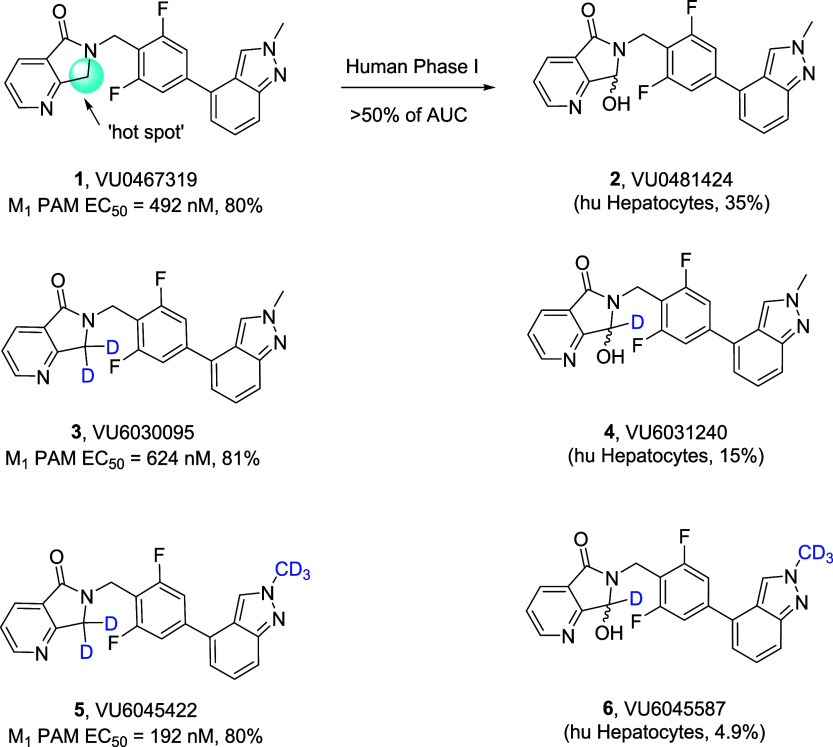
Structures of VU0467319 (**1**) and the oxidative metabolite (**2**), the dideutero congener **3** (and its metabolite **4**), and the penta-deutero analog **5** (and its oxidative metabolite **6**).

Unfortunately, an inactive metabolite **2** (VU0481424) proved to be the major circulating VU0467319-related drug product in man (VU0481424 represented >50% of plasma AUC of total VU0467319 drug administered) which led, in part, to termination of **1**, and a shift in focus to identify a back-up M_1_ PAM with diminished metabolite production.^22–26^

The team elected to implement subtle perturbations to PAM **1**, as it possessed the preferred PAM pharmacology and was well-tolerated in initial IND-enabling studies and in humans at projected therapeutic exposures. Both steric and electronic strategies, while blocking the metabolic “hot spot”, led to a significant diminution in M_1_ PAM potency. The team then explored the installation of a *gem*-dideutero moiety at the metabolic “hot spot” to assess if the kinetic isotope effect, employing a stronger C–D bond, would reduce oxidative metabolism (Figure 1).^27–29^ This exercise led to the development of dideutero VU6030095 (**3**), a surprisingly weaker M_1_ PAM than **1**, but which produced less of the analogous oxidative metabolite VU6031240 (**4**).^29^ The need for an analytical standard led to the development of pentadeutero congener VU6045422 (**5**), which produced far less of the corresponding metabolite VU6045587 (**6**) and was unexpectedly a more potent M_1_ PAM than **1**. PAM **5** displayed greatly enhanced metabolic stability *in vitro* (multispecies microsomes and hepatocytes) and *in vivo* in standard multispecies PK studies. However, in a dog Maximum Tolerated Dose (MTD) study, as well as in a high-dose rat 14-day toxicology study, the amount of oxidative metabolism of **5** was comparable to that of VU0467319 (**1**), and the development of PAM **5** was also terminated.^29^ Importantly, these data highlight how H-D bioisosterism is unpredictable in terms of both potency and disposition. Here, we detail the next iteration of M_1_ PAM back-up chemistry that produced a tertiary alcohol congener, VU6052254, a highly potent M_1_ PAM with minimal M_1_ agonism, exceptional multispecies preclinical PK, robust efficacy across multiple rodent cognition assays, and greatly diminished oxidative metabolism at the known “hot spot.”

## Results and Discussion

### Design

As discussed, all attempts to block oxidative metabolism at the benzylic lactam center of **1**, with steric, electronic, or kinetic isotope effect strategies proved both unsuccessful and unpredictable. We then decided to consider polar chemical moieties that we could introduce distant to the metabolic “hot spot” that might be able to either reduce or shunt metabolism.^22,29^ As shown in Figure 2, we evaluated a variety of polar protic (e.g., alcohols and amines) and polar aprotic (e.g., sulfones and ethers) functional groups across various positions of **1** to improve solubility and unbound fraction. Composite SAR proved very steep. Analogs were devoid of M_1_ PAM potency/efficacy, displayed greatly reduced CNS penetration and/or possessed a high degree of unacceptable M_1_ agonism. From this exercise, a single compound emerged, harboring a tertiary hydroxyl moiety^30^ at the 7-position of the indazole, VU6052254 (henceforth PAM **7**). PAM **7** (EC_50_ = 59 nM, 79%) was significantly more potent than **1** (EC_50_ = 492 nM, 80%) and in human hepatocytes incubated for 4 h at 37 °C, **7** was more stable than **1**, producing only 11% of the undesired oxidative metabolite at the known “hot spot.” However, in order to further evaluate PAM **7** as a putative backup candidate, we needed to quickly develop a multigram-scale synthetic route.

**Figure 2:**
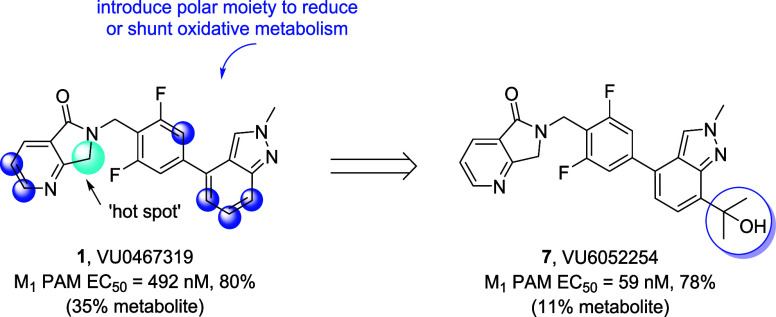
Optimization of PAM **1** to afford **7**. Various positions of **1** were substituted with polar moieties to modify the disposition. Analogs were either inactive M_1_ PAMs or engendered unacceptable degrees of M_1_ agonism. Ultimately, a single analog **7** emerged, harboring a tertiary hydroxyl moiety on the 7-position of the indazole that was more potent and more stable than **1**.

A high-yielding, convergent, six-step route for the multigram synthesis of PAM **7** was developed (Scheme 1). Starting from commercial ester **8**, indazole formation provided **9**,^31^ which was selectively *N*-methylated at the 2-position with Meerwein’s salt to deliver **10** in 75% yield for the two-step sequence. Addition of excess methyl Grignard reagent in the presence of CeCl_3_ afforded key tertiary alcohol intermediate **11** in 75% yield. In parallel, ethyl 2-formylnicotinate **12** undergoes a reductive amination with benzyl amine **13**, and intramolecular amidation gives lactam **14** in 57% yield. Conversion of the aryl bromide to the corresponding boronic ester **15** proceeds smoothly, providing the second key intermediate. Finally, a Suzuki coupling between intermediates **11** and **15** yields PAM **7** in six steps and ∼20% overall yield on multigram scales.

**Scheme 1:**
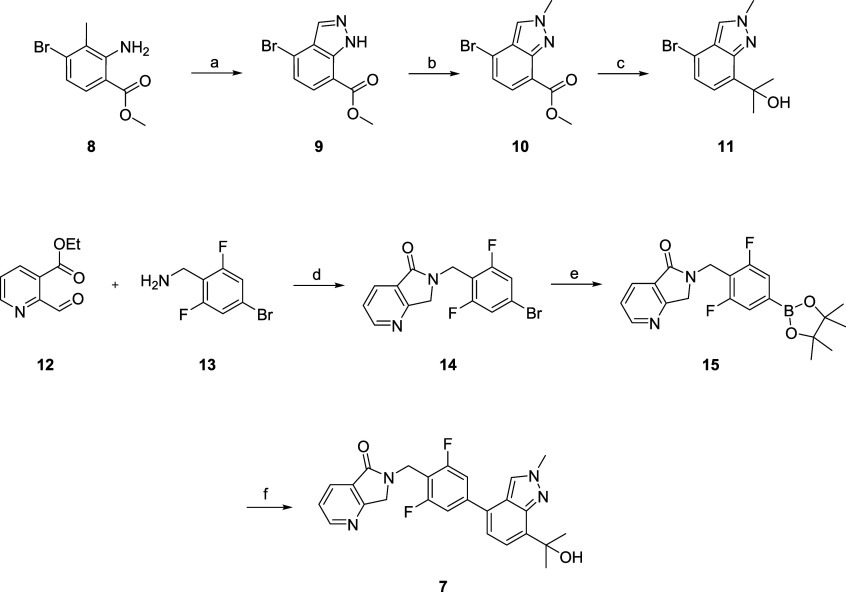
Synthesis of VU6052254 (**7**)­^a^

a Reagents and conditions: (a) i. Ac_2_O, CHCl_3_; ii. KOAc, isoamyl nitrite, 80 °C, 16 h; (b) Me_3_OBF_4_, EtOAc, rt, 16 h, 85% over two steps; (c) MeMgBr, CeCl_3_, THF, 0 °C, 75%; (d) Na­(OAc)_3_BH, DCE, rt, 16 h, 57%; (e) *bis*-pinacolborane, 5 mol % Pd­(dppf)­Cl_2_, KOAc, 1,4-dioxane, 100 °C, 2 h; (f) Cs_2_CO_3_, 5 mol % Pd­(dppf)­Cl2, 1,4-dioxane/H_2_O, 100 °C, 2 h, 57% over two steps.

### Hepatocyte Stability (*In Vitro*)

Prior to full pharmacological and DMPK characterization, PAM **7** was incubated in rat, dog, monkey, and human hepatocytes, along with **1** and **5**, to determine the amount of remaining parent PAM, as well as quantification of undesired oxidative metabolites **2**, **6,** and 1**6** (Figure 3).^22,29^ As previously reported, PAM **1** generates metabolite **2** in high levels (>23%) across all four species but is significant in human (34.9%) and monkey (74.3%), though why monkey is so high is unclear.^22,29^ The penta-deutero derivative **5** displayed far greater metabolic stability across species, generating low levels of the analogous metabolite **6** (<10%), except for monkey (21%).^29^ PAM **7**, with the tertiary alcohol moiety, was more stable (<11% across species, except for monkey, 26.8%) than **1**, but less than **5**. However, the hepatocyte stability of **7** was far better than **1** for rat, dog, and human and was therefore poised for further advancement of compound characterization.

**Figure 3:**
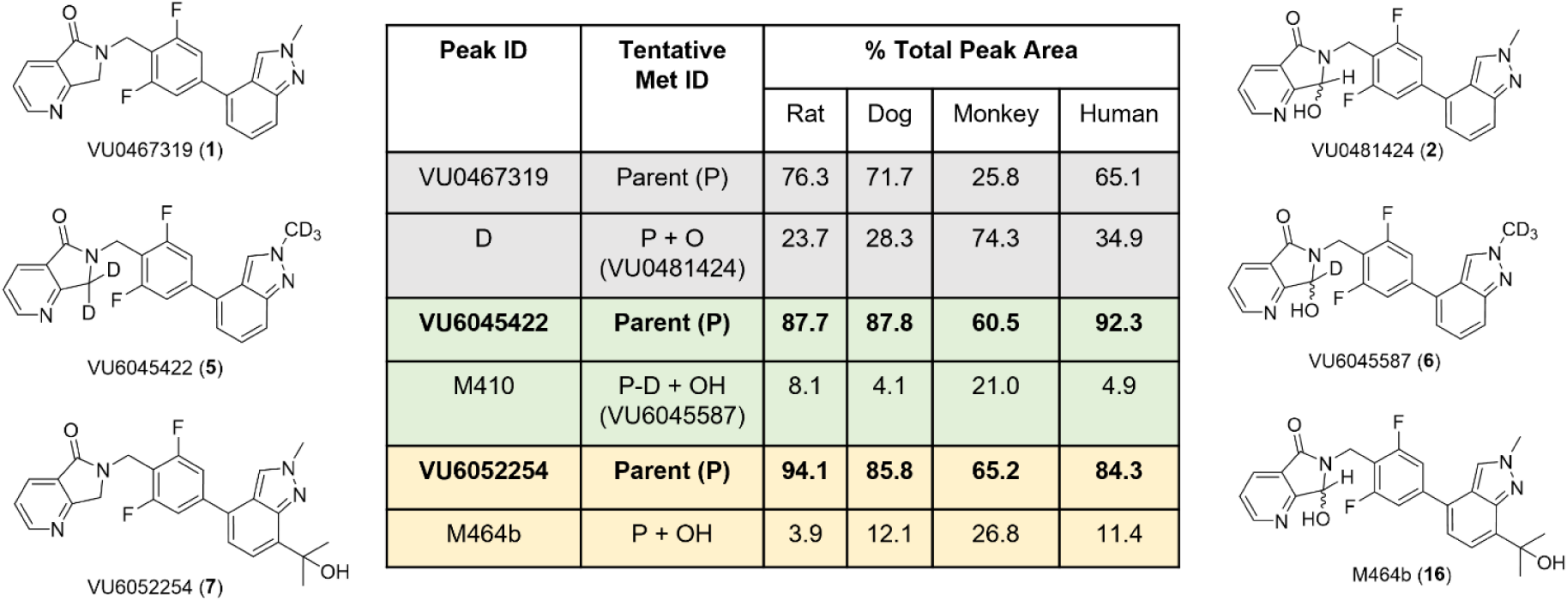
Multispecies hepatocyte metabolite stability of M_1_ PAMs **1**, **5,** and **7** and quantification of oxidative metabolites **2**, **6**, and 1**6**, respectively, produced after 4 h incubation. Both compounds **5** and **7** were metabolically more stable than compound **1** across all four species.

### Molecular Pharmacology and *In Vitro* DMPK

PAM **7** was an attractive small molecule from both a pharmacological and DMPK perspective (Table 1). Compound **7** was a potent M_1_ PAM on both human and rat M_1_ (human EC_50_ = 59 nM, 78% ACh max; rat EC_50_ = 78 nM, 94% ACh max) with minimal M_1_ agonism (human EC_50_ = 3,640 nM, 22% ACh max; rat EC_50_ > 10,000 nM, 17% ACh max). Moreover, PAM **7** was inactive on both human and rat M_2–5_ (EC_50_ *s* > 30,000 nM). In a Eurofins Lead Profiling Screen of 68 G-Protein Coupled Receptors (GPCRs), ion channels, and transporters, PAM **7** was generally devoid of off-target activities (<50% displacement of radioligands at 10 μM) with the exception of adrenergic α_2a_ (IC_50_ = 3.2 μM) and calcium L-Type rat (IC_50_ = 1.1 μM). In a functional hERG manual patch clamp (MPC) assay, PAM **7** was inactive (IC_50_ > 30 μM), and in the broader CV ion channel EP panel, PAM **7** had no significant activities. Thus, based on this attractive molecular pharmacology profile, further progression of PAM 7 was deemed warranted.

**Table 1:**
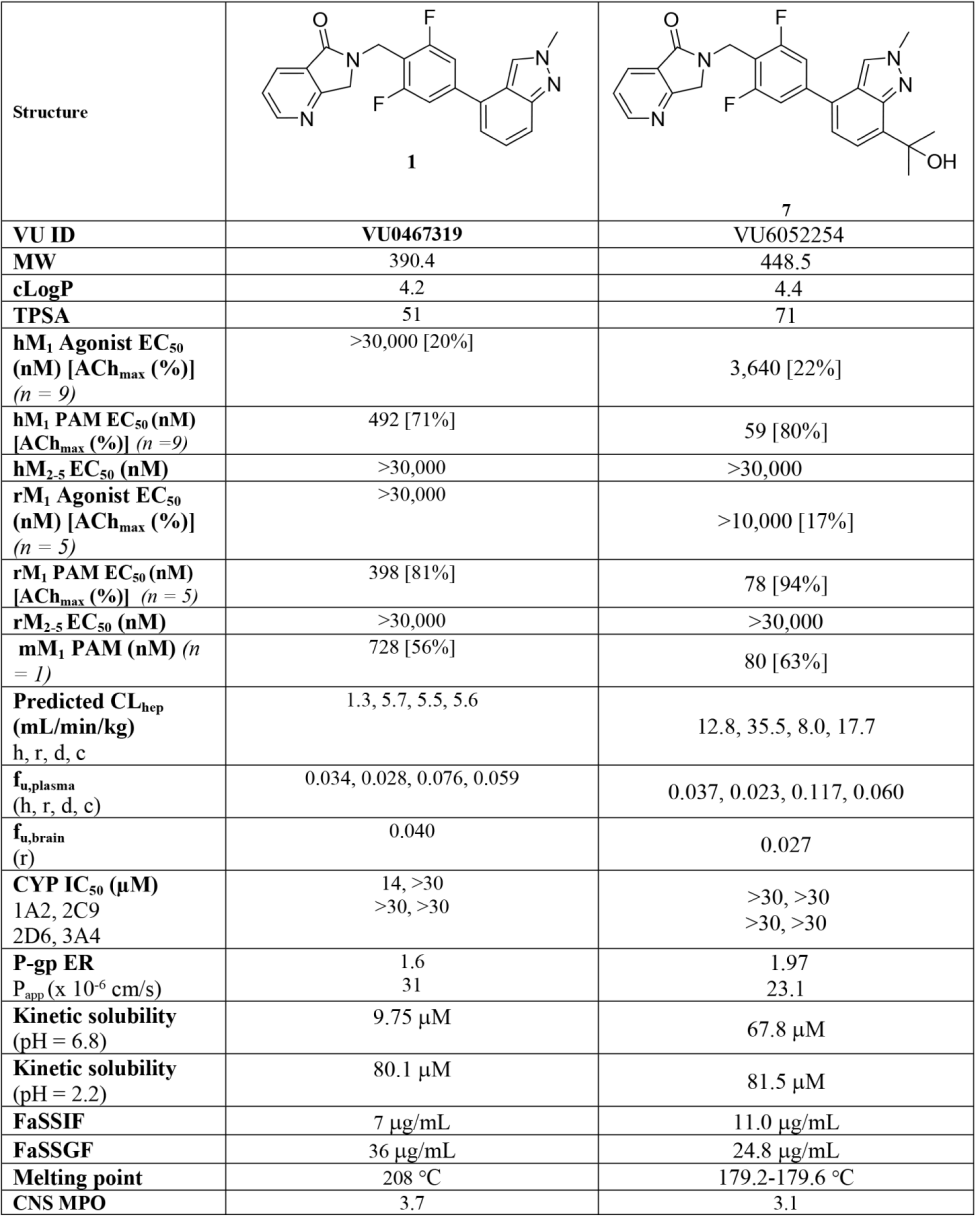
Pharmacology and *In Vitro* DMPK Profile of M_1_ PAM 7

In tier 1 *in vitro* DMPK panels, PAM **7** displayed low-to-moderate predicted hepatic clearance (h,r,d,c: CL_hep_ = 12.8, 35.5, 8.0, and 17.7 mL/min/kg) and acceptable unbound fraction in both plasma (h,r,d,c: *f*_u_ = 0.037, 0.023, 0.117, 0.060) and in rat brain (*f*_u_ = 0.027). PAM **7** had a clean CYP_450_ profile (IC_50_ > 30 μM vs 3A4, 2D6, 2C9, 2B6, 2C19, 2C8, and 1A2) with no evidence of time-dependent inhibition. Importantly, PAM **7** was predicted to be CNS penetrant in human, MDCK-MDR1 (P-gp) ER = 1.97 with high P_app_ (23.1 × 10^–6^ cm/s). PAMs **1** and **5** suffered from low aqueous solubility, which made formulation in high-dose toxicology studies challenging; fortunately, the introduction of the tertiary hydroxyl moiety in **7** led to a significant improvement in aqueous solubility under both neutral (pH 6.8 = 67.8 μM) and acidic (pH 2.2 = 81.5 μM) conditions and was mirrored in simulated intestinal (FaSSIF = 11.0 μg/mL) and gastric fluid FASSGF = 24.8 μg/mL). Finally, PAM **7** had a low, sharp melting point (179.2–179.6 °C).

### *In Vitro* Electrophysiology

Previously, we have shown that potent M_1_ ago-PAMs are known to induce long-term depression (LTD) and are cognitively disrupting.^14,16–18,22,29^ In order to derisk the weak M_1_ agonist activity of PAM **7**, electrophysiology studies in mouse native tissue layer V medial prefrontal cortex (mPFC) were performed to ensure that **7** did not induce LTD. At a concentration of 10 μM (125-fold above the mouse functional PAM EC_50_ of 80 nM), only mild changes in field excitatory postsynaptic potentials (fEPSPs) were observed (86% baseline; 112% PPR). Based on our previous experience,^14,16–18,22,29^ PAM **7** is expected to display robust pro-cognitive efficacy and maintain activity dependence of prefrontal cortical (PFC) function.

### *In Vivo* DMPK and Behavior

The multispecies *in vivo* PK for PAM **7** (Table 2) was highly attractive, exemplified by low clearance, good half-lives, and high oral bioavailability. The rat PK profile of **7** (CL_p_ = 5.8 mL/min/kg, *t*_1/2_ = 3.7 h, V_ss_ = 1.3 L/kg, and with 100%F), with a good *in vitro:in vivo* correlation (IVIVC) and excellent CNS exposure (K_p_ = 1.08; K_p,uu_ = 1.27) compared to **1**. Likewise, the dog (CL_p_ = 8.4 mL/min/kg, *t*_1/2_ = 4.1 h, V_ss_ = 2.3 L/kg, and with 65%F) and NHP (CL_p_ = 7.3 mL/min/kg, *t*_1/2_ = 4.9 h, V_ss_ = 1.5 L/kg, and with 76%F) PK profiles of **7** were exceptional and improved versus the clinical M_1_ PAM **1**. These data not only supported advancing into rat pharmacodynamic (PD) studies but also suggested that human PK predictions would be favorable. Thus, the team decided to advance PAM **7** into the next tier of profiling: high-dose mouse seizure liability, modified Irwin battery, and rat novel object recognition (NOR).

**Table 2:** IV/PO Pharmacokinetic Parameters of 7

Parameter	Rat (SD)	Dog (beagle)	NHP (cyno)
Dose (mg/kg) iv/po	1/10	1/3	1/3
CL_p_ (mL/min/kg)	5.8	8.4	7.3
V_ss_ (L/kg)	1.3	2.3	1.5
Elimination *t*_1/2_ (h)	3.7	4.1	4.9
F (%) po	>100	65	76
*C*_max_ (μM)	15.9	0.7	1.1
*T*_max_ (hr)	1	1.33	4.7
AUC (μM×hr)	110	9.0	12.4
K_p_	1.08	-	-
K_p,uu_	1.27	-	-

### Derisking Cholinergic Adverse Events (AEs)

As detailed previously, we incorporate a high-throughput phenotypic seizure liability assay (high-dose intraperitoneal (i.p.)), as mice are the most sensitive rodent species to cholinergic mechanisms, and, when the M_1_ receptor is over stimulated, mice readily display seizures detectable on the Racine scale.^14,16–18,22,29^ At 100 mg/kg IP, potent M_1_ ago-PAMs such as BQCA, rapidly initiated Racine scale 4/5 seizures 30 min postadministration.^14,16–18,22,29^ In contrast, PAM **7** at a dose of 56.6 mg/kg IP had negligible AEs and at 100 mg/kg IP (Figure 4) exhibited Racine scale scores less than 2. These effects were absent when performed in M_1_ KO mice, confirming that these AEs are in fact M_1_-mediated. Satellite IP PK studies (56.6 and 100 mg/kg IP in C57Bl6 mice, 30% captisol) demonstrated that total brain exposures were 29.8 μM (382-fold above the mouse M_1_ EC_50_) and 42.2 μM (541-fold above the mouse M_1_ EC_50_), respectively. Total brain drug levels correlate better with PD efficacy^14,16–18,22,29^ versus free drug levels in brain, as the arbitrarily selected EC_20_ value for the *in vitro* PAM EC_50_ potency determination likely underestimates endogenous ACh tone. Thus, there is a significant therapeutic index for PAM **7** from this assay, and robust target-mediated convulsions were not present. To assess peripheral cholinergic toxicity, a modified Irwin neurological battery was performed to score autonomic or somatosensory side effects (Figure 5). At a dose of 56.6 mg/kg of IP, BQCA produced significant SLUDGE (**S**alivation **L**acrimation **U**rination **D**efecation **G**astrointestinal distress **E**mesis) effects in mice. In contrast, at a dose of 56.6 mg·kg IP (382-fold above the mouse M_1_ EC_50_) minimal adverse effects or SLUDGE were noted, but at 100 mg/kg IP (541-fold above the mouse M_1_ EC_50_), there were more pronounced adverse effects seen (e.g., mice exhibited hypothermia, sedation, respiratory depression, and diarrhea between the 3 and 6 h time points). Still, if the dose and exposure required for efficacy afforded the appropriate therapeutic window, then PAM **7** could still advance.

**Figure 4:**
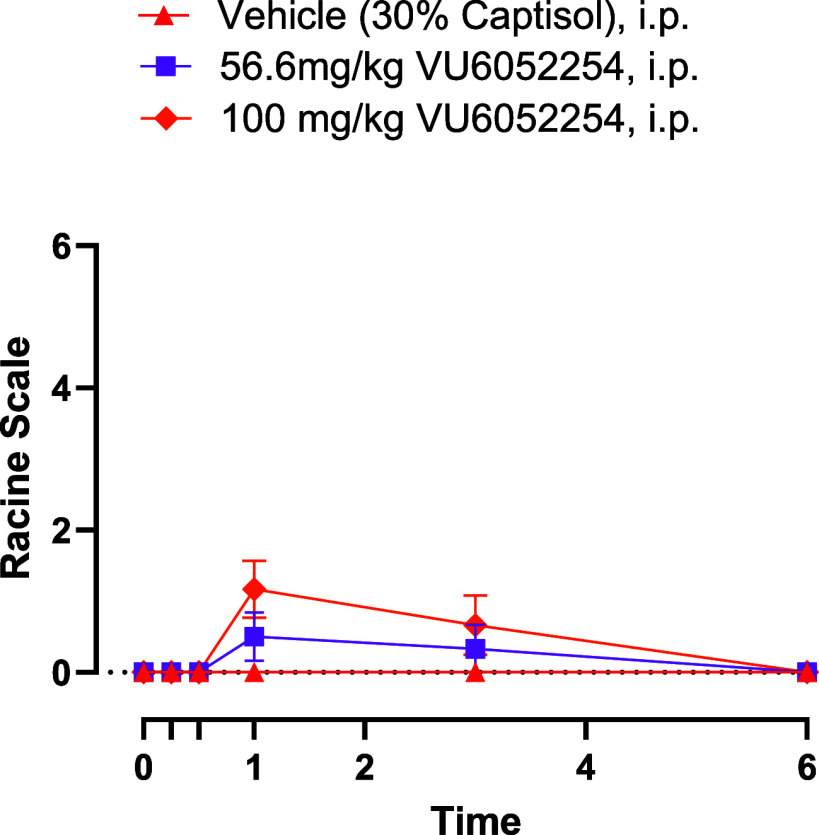
Racine score test in mice. Pretreatment with M_1_ PAM **7** (56.6 and 100 mg/kg, i.p., 10 mL/kg, 360 min) resulted in minimal AEs at 60 min postadministration with a lack of AEs observed at 6 h. *N* = 3/group of male C57Bl/6 mice.

**Figure 5:**
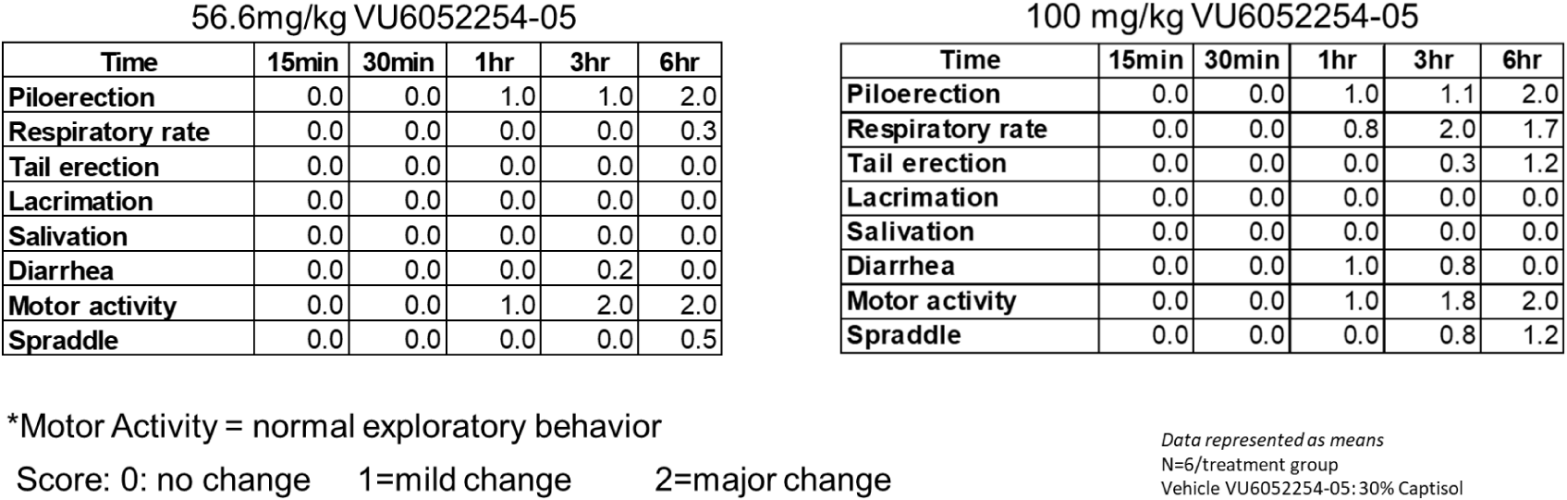
Effects of VU6052254 (**7**) on the modified Irwin Neurological Test battery in mice. At a dose of 56.6 mg/kg and over a 6 h time course, minimal adverse cholinergic events (SLUDGE) were noted. However, at 100 mg/kg of IP, adverse cholinergic events (SLUDGE) were noted.

Rat NOR has been our primary PD assay to assess rodent cognition for the M_1_ PAM program.^14,16–18,22,29^ Here (Figure 6), pretreatment of PAM **7** (0.3 to 3.0 mg/kg of PO) dose-dependently increased the recognition index during the NOR test in Sprague–Dawley rats relative to the vehicle-treated control group (*N* = 13–18). The minimum effective dose (MED) was 1 mg/kg of PO (2.65 μM total brain, ∼34x the rat M_1_ PAM EC_50_ and 71 nM free brain, or 0.91x the rat M_1_ PAM EC_50_). Relative to the seizure assay and the Irwin battery, there is an ∼50-fold margin in terms of dose, and ∼11- to 16-fold margin in terms of total brain exposure. With the AEs noted in the most sensitive species for cholinergic AEs, the team felt adequate CNS safety margins existed to continue compound assessment.

**Figure 6:**
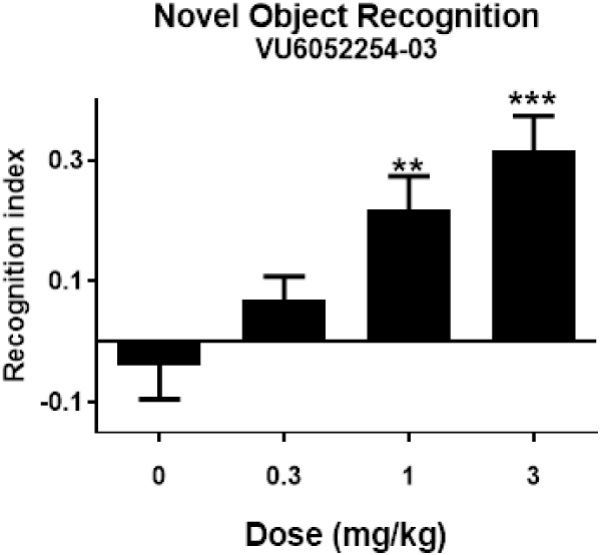
Novel object recognition (NOR) test in rats with VU6052254 (M_1_ PAM **7)**. **A)** PAM **7** dose-dependently enhanced the recognition memory in rats. Pretreatment with 0.3, 1, and 3 mg/kg **7** (PO 10% Tween 80 in water) 1 h prior to exposure to identical objects significantly enhanced recognition memory assessed 24 h later. Minimum effective dose (MED) is 1 mg/kg. *N* = 13–18/group of male Sprague–Dawley rats. ANOVA, **p < 0.01, ***p < 0.001 Dunnett posthoc test.

### Contextual Fear Conditioning

While NOR has been the primary PD driver for our M_1_ PAM programs to date, the team wanted to explore other rodent models of cognition and settled on Contextual Fear Conditioning (CFC). The CFC assay is an associative learning paradigm, involving hippocampus-mediated memory functions, in which the rat learns to associate their environment (context) with a fear-inducing stimulus (e.g., a foot shock) that elicits a “freezing response”.^32,33^ PAM **7** produced a robust dose-dependent enhancement in the acquisition of contextual fear conditioning in rats (Figure 7), with an MED of 0.3 mg/kg PO (58 nM total brain correlating to 0.74x the rat M_1_ PAM EC_50_). The effect plateaued with increasing doses/exposures without the presence of a U-shaped dose–response curve over the dose range tested (correlating to total brain drug levels 7.3x the rat M_1_ PAM EC_50_ at 3.0 mg/kg p.o.).

**Figure 7:**
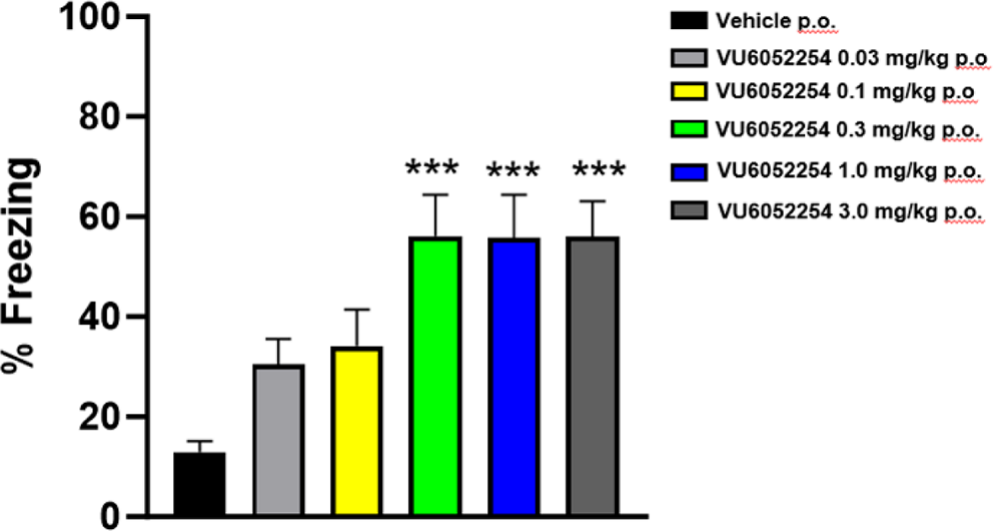
Contextual fear conditioning (CFC) test in rats with VU6052254 (M_1_ PAM **7)**. PAM **7** dose-dependently enhanced the acquisition of CFC in rats. Pretreatment with 0.03, 0.1, 0.3, 1, and 3 mg/kg **7** (PO 10% Tween 80 in water) significantly enhanced CFC. Minimum effective dose (MED) is 0.3 mg/kg. *N* = 13–18/group of male Sprague–Dawley rats. ANOVA, ***p < 0.001 Dunnett posthoc test.

Next, we wanted to evaluate the efficacy of M_1_ PAM **7** in the CFC paradigm after a pharmacological challenge. Careful titration of the ability of scopolamine, a *pan*-muscarinic antagonist, was evaluated in the assay, and a dose of 0.25 mg/kg of IP was shown to disrupt acquisition of CFC by ∼65%. As shown in Figure 8, PAM **7** was able to reverse the scopolamine-induced disruption of the acquisition of CFC with an oral MED of 1 mg/kg (correlating to total brain ∼3× the rat M_1_ PAM EC_50_).

**Figure 8:**
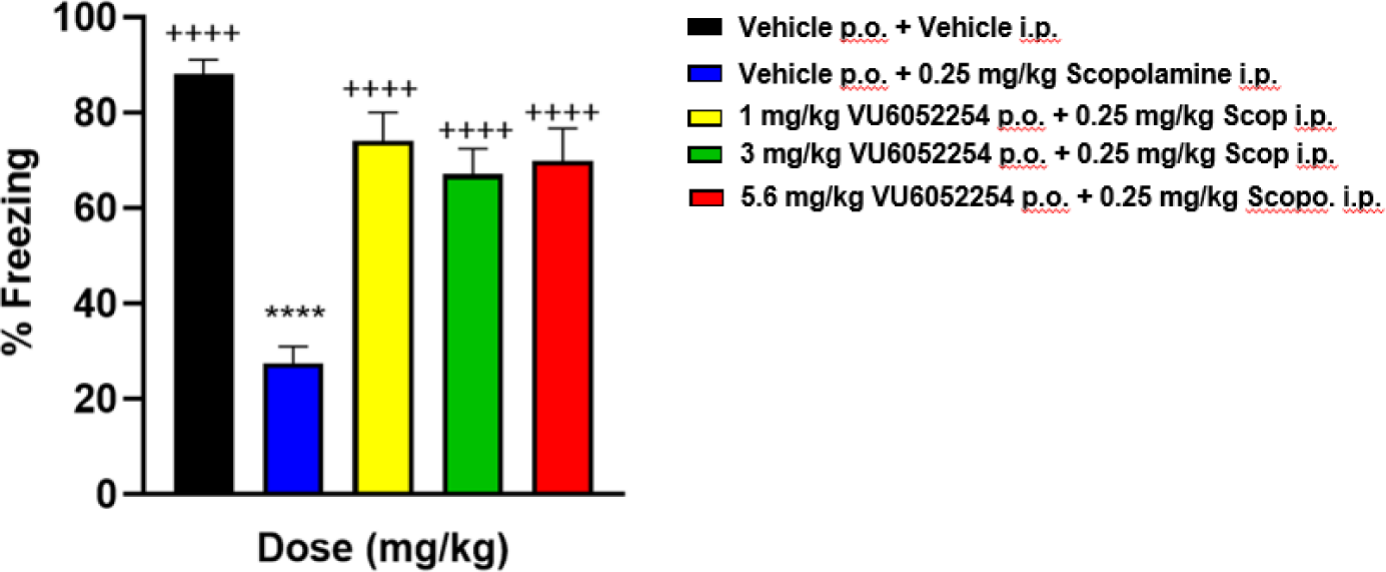
Contextual fear conditioning (CFC) test with scopolamine (0.25 mg/kg IP) challenge in rats with VU6052254 (M_1_ PAM **7)**. Scopolamine alone (0.26 mg/kg) produced a robust decrease in the acquisition of CFC pretreatment with 1, 3, and 5.6 mg/kg of PAM **7** (PO 10% Tween 80 in water) significantly reversed the scopolamine-induced disruption of CFC. Minimum effective dose (MED) is 0.3 mg/kg. *N* = 10–12/group of male Sprague–Dawley rats. ANOVA, ***p < 0.0001 Dunnett posthoc test versus vehicle: ++++ *p* < 0.0001 versus vehicle +0.25 mg/kg scopolamine.

### Further Candidate Profiling

Human PK and dose predictions indicated that PAM **7** is predicted (presented geometric mean of six methods) to be a low-clearance compound in human (CL_p_ = 2.6 mL/min/kg) with a moderate half-life (*t*_1/2_ = 5.1 h), an ideal volume (V_ss_ = 1.16 L/kg) and to have 79% oral bioavailability (see Supporting Information for details on geometric mean of six methods used for these predictions). The predicted dose for **7** ranged from 32 to 84 mg QD. Based on this analysis, PAM 7 was advanced to safety and ADME studies.

PAM **7** was negative in the AMES assay (4 strain, with and without S9), and there was no evidence of GSH trapping human microsomes. In a panel of eight recombinant enzymes, CYP_450_ phenotyping indicated that PAM **7** was metabolized predominantly by 3A4 (96.8%) with a minor contribution from 3A5 (3.2%). Earlier work demonstrated that there were no CYP_450_ inhibition or time-dependent CYP_450_ inhibition liabilities, but we needed to evaluate induction, especially since **7** is solely metabolized by 3A4/5. In human hepatocytes from three donors, PAM **7** was a weak inducer at 10 μM of 1A2 (4.9%), 2B6 (11.5%), and 3A4 (7.6%). At 30 μM, 3A4 induction increased to 30.5%, when using mRNA as the reporter. This was a clear concern as the potential exists for **7** to induce its own metabolism, though the exposure required to do so is high (30 μM). As we have seen variability between not only donors but also across CROs that conduct these assays, we repeated the induction profiling at a different CRO that evaluates not only mRNA (message) but actual protein (translation). Here, PAM **7** was found not to be an inducer of 3A4—neither mRNA nor protein; however, we would need to critically monitor exposure levels after chronic dosing to ensure no induction liability.

PAM 7 was advanced to dose-escalation studies in rats (Figure 9). Despite high exposures obtained in this study, no cholinergic signs or adverse events were noted in rats, further highlighting the cholinergic sensitivity of mice. We performed a vehicle screen in order to maximize exposure before initiating an exploratory rat single-dose and 14-day repeated-dose toxicology and toxicokinetic study, identifying PEG-400 as an optimal vehicle for providing higher, dose-proportional systemic exposures for PAM **7** (Figure 10).

**Figure 9:**
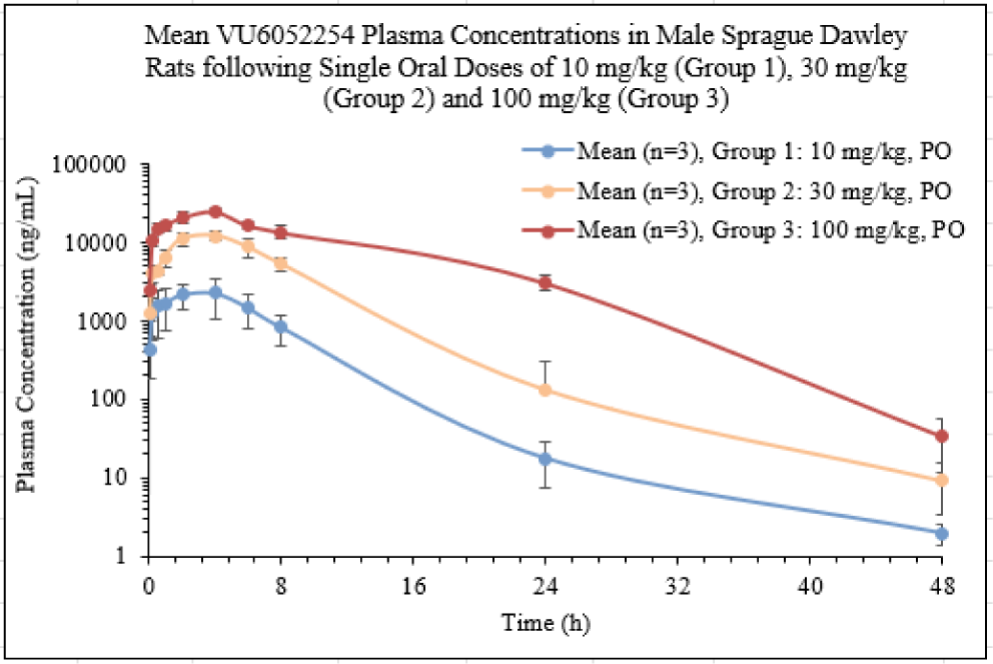
Rat oral dose escalation PK study at 10 (AUC last 23,800 h*ng/mL; *C*_max_ = 2,340 ng/mL), 30 (AUC last 66,500 h*ng/mL; *C*_max_ = 7,640 ng/mL) and 100 mg/kg (AUC last 96,200 h*ng/mL; *C*_max_ = 10,900 ng/mL) in 10% aqueous Tween 80 suspension dosing.

**Figure 10:**
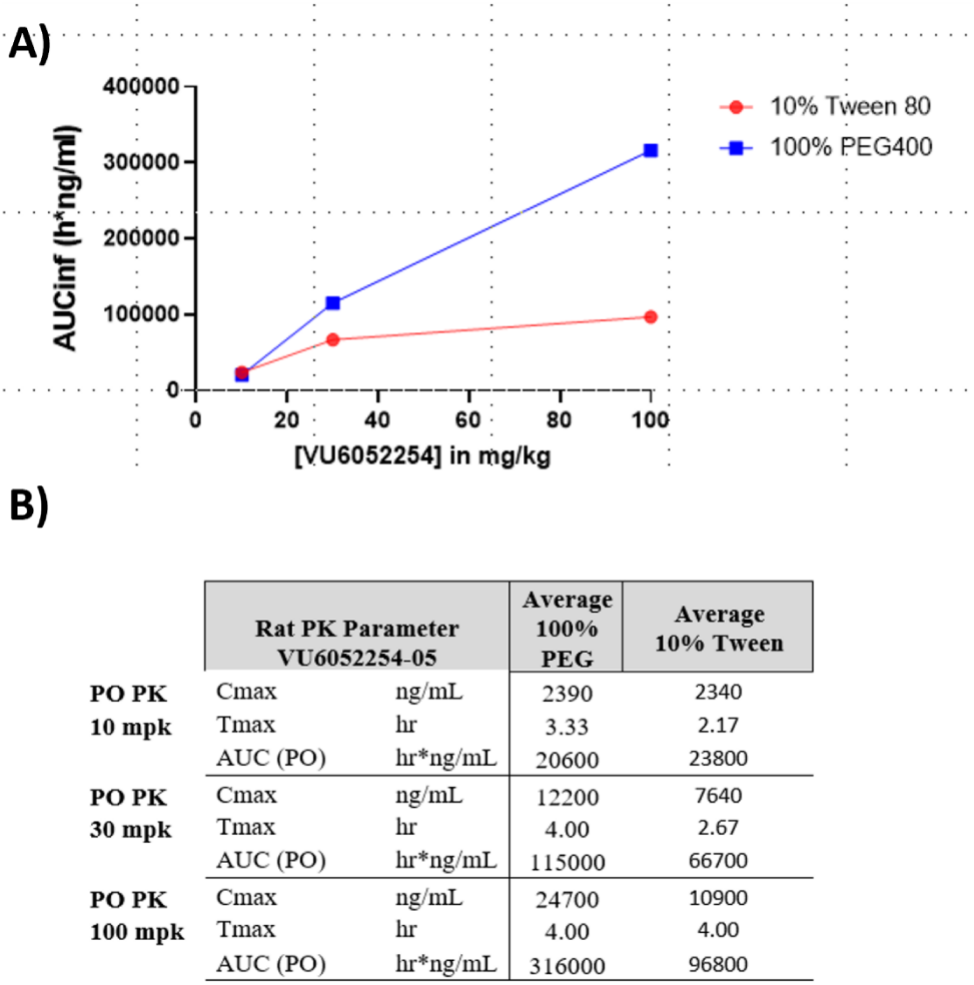
Rat oral dose escalation PK study at 10, 30, and 100 mg/kg in 10% aqueous Tween 80 suspension and PEG-400 soluble dosing. Total exposure for *C*_max_ and oral AUC was higher in PEG-400 and the soluble vehicle dose escalated in a near-linear fashion at higher doses.

PAM **7**, after a 250 g scale-up, advanced into 14-day rat toxicology studies, with vehicle (PEG-400), 30 mg/kg PO and 300 mg/kg PO cohorts, *N* = 6 per sex per cohort. Day 1 exposures were *C*_max_ = 20.9 μM, AUC_last_ = 224 h*μM at 30 mg/kg and *C*_max_ = 37.9 μM, AUC_last_ = 758 h*μM at 300 mg/kg. At Day 14, exposure in the 30 mg/kg cohort decreased to *C*_max_ = 10.0 μM, AUC_last_ = 107 h*μM at 30 mg/kg but increased to *C*_max_ = 52.9 μM, AUC_last_ = 909 h*μM in the 300 mg/kg cohort. No cholinergic/SLUDGE adverse effects were observed, even though total plasma concentrations were 125x and 227x the plasma concentration at MED in the CFC assay (0.3 mg/kg PO). There were no PAM **7**-related effects noted in hematology test results in males administered up to 30 mg/kg, and only a minimally higher fibrinogen concentration in males administered 300 mg/kg. There were no gross changes on organ weights or histology. Higher serum creatinine concentrations were however noted in males at both dose levels (+100% at 30 mg/kg/day and +300% at 300 mg/kg/day). Microscopic pathology evaluation of renal cortex and medulla revealed a dose-related increase in number of pyknotic cells in the lumen of descending tubules/proximal collecting tubules (e.g., tubular degeneration). At the higher dose of 300 mg/kg/day, pyknotic cells were also seen in collecting tubules, and less extensively, in the renal papilla. Based on the observations of correlative clinical and anatomic kidney toxicity findings in an exploratory 14-day toxicity and toxicokinetics rat study, in conjunction with uncertain margins to projected systemic human therapeutic efficacy and CYP3A4 induction concerns, the development of PAM **7** was discontinued.

## Conclusions

A second-generation chemical optimization back-up program for VU0467319, an M_1_ PAM clinical candidate that successfully completed a Phase I SAD clinical trial, is reported. In an attempt to shunt metabolism, strategic introduction of a tertiary hydroxyl moiety afforded VU6052254 (PAM 7), a potent M_1_ PAM (EC_50_ = 59 nM, 79% ACh max), with high CNS exposure (rat K_p_ = 1.07; K_p,uu_ = 1.27; P-gp ER = 1.97, P_app_ = 23 × 10^–6^ cm/s) and reduced oxidative metabolism across species. The tertiary hydroxyl imparted significantly improved physiochemical properties and aqueous solubility, which led to excellent pharmacodynamic responses (MED in rat NOR and scopolamine CFC = 1 mg/kg of PO; MED in rat CFC = 0.3 mg/kg of PO). The low clearance and high oral bioavailability across nonclinical species (Cl_p_ *s* < 10 mL/min/kg, %*F* > 65) afforded favorable human PK and dose projections. A 14-day toxicology study in rats achieved high exposures with no cholinergic adverse effects; however, correlative dose-related clinical and anatomic kidney findings in 14-day rat toxicity studies (not observed with VU0467319) at uncertain exposure margins to the projected human efficacious dose along with *in vitro* CYP_450_ induction liability concerns precluded further development. VU6052254, however, represents a robust M_1_ PAM tool compound, with minimal agonism to study selective M_1_ activation in rodent PD models.

## Supplementary Material



## Notes

Additional experimental details, methods for the synthesis and characterization of all compounds, *in vitro* and *in vivo* DMPK protocols, and supplemental figures (PDF)

## ABBREVIATIONS

PAM: positive allosteric modulator;
PBL: plasma/brain level;
DMPK: drug metabolism and pharmacokinetics;
AE: adverse event;
M_1_: muscarinic acetylcholine receptor subtype 1;
MED: minimum effective dose;
NOR: novel object recognition;
CFC: contextual fear conditioning
