# Supplementary material for: Discovery of VU6052254: A Novel, Potent M1 Positive Allosteric Modulator
Source: ACS Chem Neurosci. 2025 Nov 26;16(24):4741–50. doi: 10.1021/acschemneuro.5c00825 (PMC12715762; doi:10.1021/acschemneuro.5c00825)
Supplement: Supplementary file 1 [file cn5c00825_si_001.pdf]

Supporting Information for

Discovery of VU6052254: A Novel, Potent M_1 Positive Allosteric Modulator

Julie L. Engers,[1,2] Joseph D. Bungard,[1,2] Christopher C. Presley,[1,2] Irene Zagol-Ikapitte,[1,2] Katherine J. Watson,[1,2] Sichen Chang, [1,2] Colin O'Carroll[5], P. Markus Dey,[5] Ethan S. Burstein,[5] Michael Bubser,[1,2] Jerri M. Rook[1,2], Hyekyung P. Cho,[1,2] Valerie M. Kramlinger,[1,2] Olivier Boutaud,[1,2] Carrie K. Jones,[1,2] P. Jeffrey Conn,[1,2,4] Darren W. Engers,[1,2] and Craig W. Lindsley*[1,2,3,4,6]

Affiliation:

[1]Warren Center for Neuroscience Drug Discovery, Vanderbilt University, Nashville, TN 37232, USA

[2]Department of Pharmacology, Vanderbilt University School of Medicine, Nashville, TN 37232, USA

[3]Department of Chemistry, Vanderbilt University, Nashville TN 37232, USA

[4]Vanderbilt Institute of Chemical Biology, Vanderbilt University, Nashville, TN 37232, USA

[5]Acadia Pharmaceuticals Inc., San Diego, CA 92130, USA

[6]Vanderbilt Integrative Discovery Institute, Vanderbilt University, Nashville, TN 37232, USA

*To whom correspondence should be addressed at craig.lindsley@vanderbilt.edu

TABLE OF CONTENTS

Eurofins Lead Profiling Screen....S2

Charles River Functional hERG....S4

Charles River Cardiac EP Panel....S5

Procedures for Biological Experiments....S6

General Methods....S13

Synthetic Procedures and Spectra....S15

Supplemental Figures....S64

Table S1. Eurofins Lead Profiling Screen Data - Study TW04-0011004

This is a radioligand binding panel of 67 targets including GPCRs, ion channels, transporters and nuclear hormones. Biochemical assay results are presented as the percent inhibition of specific binding at a 10 μM concentration of **VU6052254** (**7**).

**Target/Protein**	**Species**	**% Inhibition at 10 μM**
Adenosine A_1	Human	-2
Adenosine A_{2A}	Human	3
Adenosine A_3	Human	8
Adrenergic α_{1A}	Human	13
Adrenergic α_{1B}	Human	8
Adrenergic α_{1D}	Human	11
Adrenergic α_{2A}	Human	75
Adrenergic β_1	Human	5
Adrenergic β_2	Human	-4
Androgen (Testosterone)	Human	-4
Bradykinin B_1	Human	0
Bradykinin B_2	Human	-18
Calcium Channel L-Type, Benzothiazepine	Rat	80
Calcium Channel L-Type, Dihydropyridine	Rat	59
Calcium Channel N-Type	Rat	-11
Cannabinoid CB_1	Human	25
Dopamine D_1	Human	6
Dopamine D_{2S}	Human	-2
Dopamine D_3	Human	-10
Dopamine $D_{4.2}$	Human	-5
Endothelin ET_A	Human	11
Endothelin ET_B	Human	6
Epidermal Growth Factor (EGF)	Human	-14
Estrogen ERα	Human	12
$GABA_A$, Flunitrazepam, Central	Rat	4
$GABA_A$, Muscimol, Central	Rat	-10
$GABA_{B1A}$	Human	7
Glucocorticoid	Human	9
Glutamate, Kainate	Rat	-2
Glutamate, NMDA, Agonism	Rat	-3
Glutamate, NMDA, Glycine	Rat	-9
Glutamate, NMDA, Phencyclidine	Rat	-1
Histamine H_1	Human	4
Histamine H_2	Human	2
Histamine H_3	Human	-11
Imidazoline I_2, Central	Rat	64

Interleukin IL-1 R1	Human	-10
Leukotriene, Cysteinyl $CysLT_1$	Human	8
Melatonin MT_1	Human	-1
Muscarinic M_1	Human	18
Muscarinic M_2	Human	3
Muscarinic M_3	Human	7
Neuropeptide Y Y_1	Human	-10
Neuropeptide Y Y_2	Human	-9
Nicotinic Acetylcholine α3β4	Human	4
Nicotinic Acetylcholine α1, Bungarotoxin	Human	2
Opiate δ_1 (OP1, DOP)	Human	-2
Opiate κ (OP2, KOP)	Human	15
Opiate μ (OP3, MOP)	Human	2
Phorbol Ester	Mouse	12
Platelet Activating Factor (PAF)	Human	1
Potassium Channel [K_{ATP}]	Hamster	11
Potassium Channel hERG	Human	2
Prostanoid EP_4	Human	21
Purinergic P2X	Rat	4
Purinergic P2Y, Non-Selective	Rat	21
Rolipram	Rat	61
Serotonin (5-HT_{1A})	Human	12
Serotonin (5-HT_{2B})	Human	20
Serotonin (5-HT_3)	Human	-14
Sigma σ_1	Human	6
Sodium Channel, Site 2	Rat	22
Tachykinin NK_1	Human	4
Thyroid Hormone	Rat	-1
Transporter, Dopamine (DAT)	Human	7
Transporter, GABA	Rat	-12
Transporter, Norepinephrine (NET)	Human	1
Transporter, Serotonin (SERT)	Human	-1

Cat #	Assay Name	Species	Conc.	% Inh.	IC_{50}*	K_i	n_H
203630	Adrenergic α_{2A}	hum	10 μM	80	3.15 μM	1.57 μM	1.20
214510	Calcium Channel L-Type, Benzothiazepine	rat	1 μM	52	1.06 μM	0.94 μM	1.04

Results

Table S2: Effects of VU6052254-03 on hERG Currents

Test Article ID	Conc (µM)	Mean % hERG Inhibition	Standard Deviation	Standard Error	n	Individual Data Points (% Inhibition)
VU6052254-03	1	4.7	2.9	1.5	4	7.9
						5.6
						4.4
						0.8
	3	9.2	3.9	1.9	4	9.0
						13.9
						9.4
						4.4
	10	17.9	4.0	2.0	4	20.7
						20.3
						12.0
						18.5
	30	42.7	6.7	3.4	4	45.9
						41.7
						33.8
						49.3
	100*	17.9	5.7	3.3	3	20.2
						22.1
						11.5

* Formulation contained precipitation

Table S3: Effects of VU6052254-03 on Ion Channel Currents

Ion Channel	Test Article ID	Conc (µM)	Mean % Inhibition	Standard Deviation	Standard Error	n	Individual Data Points (% Inhibition)
hCav1.2	VU6052254-03	10	27.9	11.7	6.7	3	30.6
							37.9
							15.0
hCav3.2	VU6052254-03	10	6.4	5.0	2.0	6	-0.4
							13.6
							3.4
							10.3
							5.3
							6.4
hHCN2	VU6052254-03	10	14.8	6.9	3.4	4	21.1
							10.5
							7.4
							20.4
hKv1.3	VU6052254-03	10	-33.1	5.0	2.0	6	-25.2
							-36.6
							-34.4
							-35.3
							-38.0
							-28.8
hKv1.5	VU6052254-03	10	3.7	8.8	4.4	4	15.9
							3.3
							0.1
							-4.7
hKvLQT1/minK	VU6052254-03	10	11.9	5.0	2.9	3	16.9
							12.0
							6.9
hNav1.5 (Tonic)	VU6052254-03	10	4.9	6.6	3.8	3	8.4
							-2.7
							9.1
hNav1.5 (Phasic)	VU6052254-03	10	13.8	8.0	4.6	3	16.6
							4.8
							20.0

Procedures for Biological Experiments

Calcium mobilization assays:

All functional cell-based assays were performed in stable Chinese Hamster Ovary (CHO) cell lines constitutively expressing human M_5 or human M_1 receptors. For full muscarinic selectivity, CHO cells expressing human M_3, human M_2 plus G_{qi5} or human M_4 plus G_{qi5} were used. Cells were plated at 15,000 cells per 20 μL per well in black 384-well, TC-treated, clear-bottomed plates (Greiner) in Ham's F12 medium supplemented with 10% FBS and 20 mM HEPES. Cells were incubated overnight at 37 °C under 5% CO_2. The following day, the medium was removed and replaced with 1.2 μM Fluo-4 AM (Invitrogen) in assay buffer (Hank's Balanced Salt Solution supplemented with 20 mM HEPES and 2.5 mM Probenecid, pH 7.4) and the cells were incubated for 50 minutes at 37 °C under 5% CO_2. Dye was then removed and replaced with 20 μL of fresh assay buffer. Test compounds at a 10 mM concentration in DMSO were serially diluted in DMSO (either 1:3 or 1:5 dilution) to create a 10-point concentration series. The DMSO solutions were then diluted in assay buffer resulting in compound solutions at 2-times the final assay concentration with the highest assay concentration of 30 μM. The compound plate, cell plate, and plates containing EC_{20} and EC_{80} acetylcholine concentrations were placed in a Hamamatsu FDSS 6000 or 7000 kinetic imaging plate reader equipped to measure Ex_{480}/Em_{540} fluorescence. Data were collected at 1 frame per second. After 2 seconds of collecting baseline fluorescence, 20 μL of the compound solutions were added to the cell plate. This was followed by the addition of an EC_{20} concentration of acetylcholine at 142 seconds. At 267 seconds, an EC_{80} concentration of acetylcholine was added along with a maximally effective acetylcholine concentration in wells not containing a compound to allow data normalization. The fluorescence signal was collected for a total of 300 seconds. Compound concentration response curves (CRCs) were collected in triplicate across three separate plates. Data were imported and analyzed in Dotmatics Informatics software. by normalizing all data in the individual kinetic traces to the initial fluorescence read. The magnitude of each agonist addition was then determined and normalized to the average maximum response. This percent maximum response was plotted against log[compound] and fit to a four parameter logistical equation to determine log(IC_{50}). The IC_{50} determined using the EC_{80} of acetylcholine is the value reported. Compound CRC curves that did not plateau below 10% ACh_{max} were assigned a low potency value of >10 μM.

Drug Metabolism Methods:

In vitro

Plasma protein binding and Brain homogenate binding: Determination of fraction unbound (f_u) in plasma was conducted in vitro via equilibrium dialysis using HTDialysis (HTD) membrane plates. The top half of the plate was filled with 100 uL of Dubelco's Phosphate Buffered Saline, pH 7.4 (DPBS). Compounds were diluted into plasma from each species (5 µM final concentration), which was aliquoted in triplicate to the 'bottom half' of the prepared HTD plate wells. The HTD plate was sealed and incubated for 6 hours at 37 ˚C. Following incubation, each well (both top and bottom halves) were transferred (20 µL) to the corresponding wells of a 96-shallow-well (V-bottom) plate. The daughter plates were then matrix-matched (DPBS side wells received equal volume of plasma, and plasma side wells received equal volume of DPBS), and extraction solution (120 µL; acetonitrile containing 50 nM carbamazepine as IS) was added to all wells of both daughter plates to precipitate protein and extract test article. The plates were then sealed and centrifuged (3500 rcf) for 10 minutes at ambient temperature. Supernatant (60 µL) from each well of the daughter plates was then transferred to the corresponding wells of new daughter plates (96-shallow-well, V bottom) containing water (Milli-Q, 60 µL/well), and the plates were sealed in preparation for LC-MS/MS analysis (see LC-MS/MS analysis method below).

The unbound fraction (f_u) was calculated following the equation below, and mean values for each species were calculated from 3 replicates.

A similar approach was used to determine the degree of brain homogenate binding, which employed the same methodology and procedure with the following modifications: 1) a final compound concentration of 1 µM was used, 2) naïve rat brains were homogenized in DPBS (1:3 composition of brain: DPBS, w/w) using a Mini-Bead Beater™ machine in order to obtain brain homogenate, which was then treated in the same manner as the plasma samples in the previously described plasma protein binding assay. Fraction unbound for both plasma and brain samples was determined using Equation 4.

$$f_u = \frac{Conc_{buffer}}{Conc_{plasma}}$$

Equation 4 Determination of fraction unbound in plasma.

The diluted fraction unbound (f_u2) in brain was calculated in the same manner by using brain homogenate rather than plasma. Undiluted fraction unbound for the brain was calculated using Equation 5

$$f_u = \frac{1/4}{\left\{\left(\frac{1}{fu2}\right) - 1\right\} + 1/4}$$

Equation 5 Determination of fraction unbound in brain. F_u2 represents the diluted fraction unbound.

Intrinsic clearance: Human or rat hepatic microsomes (0.5 mg/mL) and 1 µM test compound were incubated in 100 mM potassium phosphate pH 7.4 buffer with 3 mM $MgCl_2$ at 37 °C with constant shaking. After a 5 min preincubation, the reaction was initiated by the addition of NADPH (1 mM). At selected time intervals (0, 3, 7, 15, 25, and 45 min), aliquots were taken and subsequently placed into a 96-well plate containing cold acetonitrile with internal standard (50 ng/mL carbamazepine). Plates were then centrifuged at 3000 rcf (4 °C) for 10 min, and the supernatant was transferred to a separate 96-well plate and diluted 1:1 with water for LC/MS/MS analysis. The *in vitro* half-life ($_{1/2}$, min, Eq. 1), intrinsic clearance (CL_{int}, mL/min/kg, Eq. 2), and subsequent predicted hepatic clearance (CL_{hep}, mL/min/kg, Eq. 3) was determined employing the following equations:

(1) $T_{1/2} = \frac{Ln(2)}{K}$

where k represents the slope from linear regression analysis of the natural log percent remaining of a test compound as a function of incubation time

(2) $CL_{int} = \frac{0.693}{in\ vitro T_{1/2}}\ x\ \frac{mL\ incubation}{mg\ microsomes}\ x\ \frac{45\ mg\ microsomes}{gram\ liver}\ x\ \frac{20^{a}\ gram\ liver}{kg\ body\ wt}$

[a]scale-up factors: of 20 (human) or 45 (rat)

(3) $CL_{hep} = \frac{Q_h \cdot CL_{int}}{Q_h + CL_{int}}$

where Q_h (hepatic blood flow, mL/min/kg) is 21 (human) or 70 (rat).

LC/MS/MS Bioanalysis of Samples from Plasma Protein Binding and Intrinsic Clearance Assays:

Samples were analyzed on a Thermo Electron TSQ Quantum Ultra triple quad mass spectrometer (San Jose, CA) via electrospray ionization (ESI) with two Themo Electron Accella pumps (San Jose, CA), and a Leap Technologies CTC PAL autosampler (Carrboro, NC). Analytes were separated by gradient elution on a dual column system with two Thermo Hypersil Gold (2.1 x 30 mm, 1.9 µm) columns (San Jose, CA) thermostated at 40 °C. HPLC mobile phase A was 0.1% formic acid in water and mobile phase B was 0.1% formic acid in acetonitrile. The gradient started at 10% B after a 0.2 min hold and was linearly increased to 95% B over 0.8 min; hold at 95% B for 0.2 min; returned to 10% B in 0.1 min. The total run time was 1.3 min and the HPLC flow rate was 0.8 mL/min. While pump 1 ran the gradient method, pump 2 equilibrated the alternate column isocratically at 10% B. Compound optimization, data collection, and processing was performed using Thermo Electron's QuickQuan software (v2.3) and Xcalibur (v2.0.7 SP1).

***In vivo* DMPK experimental:**

Determination of brain to plasma ratio:

Animal care and use

Adult male Sprague-Dawley rats weighing 275 - 299 g were obtained from Envigo (Indianapolis, Indiana) for the behavioral and pharmacokinetic studies. Rats were group-housed (2 - 3 animals per cage) under a 12 h light/12-h dark cycle with food and water ad libitum.

Adult male C57BL/6J mice aged 8 – 10 weeks were obtained from Jackson Laboratories (Bar Harbor, Maine) and were group-housed (5 animals per cage) under a 12 h light/12-h dark cycle with food and water ad libitum.

All animal study procedures were approved by the Institutional Animal Care and Use Committee and were conducted in accordance with the National Institutes of Health regulations of animal care

covered in Principles of Laboratory Animal Care (National Institutes of Health). All rats were fasted overnight prior to testing.

In-life phase

For determination of the brain over plasma ratio (K_p), compounds were formulated in 8% ethanol, 32% PEG400 and 60% DMSO (*v/v/v*) and administered as a single 0.2 mg/kg IV dose (1 mL/kg) to male, Sprague Dawley rats (*n* = 1) via injection into a surgically-implanted jugular vein catheter. At 15 min post dosing, blood sample was collected into chilled, K_2EDTA anticoagulant-fortified tube and immediately placed on wet ice. The blood sample was then centrifuged (1700 rcf, 5 minutes, 4 ˚C) to obtain plasma sample. At the same post-administration time point, whole brain sample was obtained by rapid dissection, rinsed with PBS, and immediately frozen in individual tissue collection box (dry ice). All brain and plasma samples were stored at -80 ˚C until analysis by LC-MS/MS.

Sample Analysis: Concentrations in plasma and brain homogenates were quantified by liquid chromatography tandem mass spectrometry (LC-MS/MS). Whole brains were homogenized in 3 mL of 70:30 IPA:water in a mini bead beater for 3 min, and centrifuged at 3,500 g for 5 min. 5 uL of the supernatant was diluted in 15 uL of blank plasma for quantification of the analytes. Plasma samples were centrifuged at 3,500 g for 5 min. A standard curve was generated by diluting the analytes DMSO stocks with blank plasma to obtain a final concentration of 10,000 ng/ml followed by a serial dilution down to 0.5 ng/ml. Quality controls were generated by a serial dilution of the 5,000 ng/ml standard curve solution in blank plasma to obtain 3 concentrations of 500, 50, and 5 ng/ml. 20 uL of brain diluted in plasma, plasma, blank plasma, standard curve and QC samples were loaded in a V-bottom 96-well plate. 120 uL of acetonitrile containing 0.05 uM carbamazepine (internal standard) was added to each well and the plate was centrifuged at 3,500 g for 5 min. 60 uL of the supernatant of each well (protein free) was transferred to a new 96-well plate containing 60 uL of water. The plates were sealed for analysis by LC-MS/MS.

Plasma and brain tissue samples originating from *in vivo* studies were analyzed by electrospray ionization using an AB Sciex Q-TRAP 5500 (Foster City, CA) that was coupled to a Shimadzu LC-20AD pump (Columbia, MD) and a Leap Technologies CTC PAL auto-sampler (Carrboro, NC). Analytes were separated by gradient elution using a C18 column (3 x 50 mm, 3 mm; Fortis Technologies Ltd, Cheshire, UK) that was thermostated at 40 °C. HPLC mobile phase A was 0.1% formic acid in water (pH unadjusted); mobile phase B was 0.1% formic acid in acetonitrile (pH

unadjusted). A 10% B gradient was held for 0.2 min and was linearly increased to 90% B over 0.8 min, with an isocratic hold for 0.5 min, before transitioning to 10% B over 0.05 min. The column was re-equilibrated (1 min) before the next sample injection. The total run time was 2.55 min, and the HPLC flow rate was 0.5 ml/min. The source temperature was set at 500 °C, and mass spectral analyses were performed using a Turbo-Ion spray source in positive ionization mode (5.0-kV spray voltage) and using multiple-reaction monitoring of transitions specific for the analytes. All data were analyzed using AB Sciex Analyst 1.5.1 software.

Brain plasma concentration ratio (K_p) was calculated by dividing brain concentration by plasma concentration for each animal. Unbound brain to unbound plasma concentration ratio ($K_{p,uu}$) is calculated using the following formula: $K_{p,uu}$ = (Brain ng/g x brain fu)/(plasma ng/ml x plasma fu).

Pharmacokinetic profiles in rats following oral single escalating doses

Single escalating oral dosing in Sprague-Dawley rats was performed at Frontage Laboratories according to their non-GLP Standard Operating Procedure and IACUC protocols. In short, compounds were formulated in 10% Tween 80 in water and dosed at 10 mg/kg. At different times, arterial blood was collected from a femoral artery catheter, and compound concentration was determined in plasma by LC-MS/MS following their non-GLP protocol. PK parameters were determined using Phoenix WinNonlin software (version 6.3).

In-vitro determination of blood-brain barrier penetration potential

Blood-brain barrier penetration was determined using MDR1-MDCK cell monolayers by Absorption Systems, following their protocol. In short, compounds were incubated at 5 mM final concentration on one side of the cell monolayer for 2 hours. Compounds concentration on either side of the monolayer was determined by LC-MS/MS and apparent permeability and efflux ratio were determined as described in Wang, Q. et al.[1]

Behavioral Manifestations of Seizure Activity

To evaluate induction of behavioral manifestation of seizure activity, C57Bl/6 mice received administration of vehicle or 100 mg/kg M_1 PAM **5**. Compound was formulated in 30% Captisol (pH 7.0) at a concentration of 10 mg/mL and injected i.p. at 10 ml/kg ($n = 4$). Animals were

monitored continuously and scored for behavioral manifestations of seizure activity at 5, 15, 30 min, and 1 h. Behavioral manifestations of seizures were scored using a modified Racine scoring system. Briefly, a score of 0 represents no behavior alterations; score 1, immobility, mouth and facial movements, or facial clonus; score 2, head nodding, tail extension; score 3, forelimb clonus, repetitive movements; score 4, rearing and tonic clonic seizure; and score 5, continuous rearing and falling, severe generalized tonic clonic seizure.

Novel Object Recognition Task

Rats were habituated for 10 min for 2 consecutive days in an empty novel object recognition (NOR) arena consisting of dark-colored plexiglass box (40 × 64 × 33 cm^3). On day 3 rats were administered vehicle, M_1 PAM **3** (1-10 mg/kg, p.o.., 10 mL/kg, *n* = 13-18), or M_1 PAM **5** (0.3-3 mg/kg, p.o.., 10 mL/kg, *n* = 16-18) and returned to their home cage for 60 min (M_1 PAM **5**) or 2 hr (M_1 PAM **3**. Rats were then placed in the NOR arena containing two identical objects for 10 min. Following the exposure period, rats were placed back into their home cages for 24 h. The rats were then returned to the arena in which one of the previously exposed (familiar) objects was replaced by a novel object and were video recorded for 5 min while they explored the two objects. Time spent exploring each object was scored by an observer blinded to the experimental conditions and the recognition index was calculated as [(time spent exploring novel object) – (time spent exploring familiar object)]/total time exploring objects.

Contextual Fear Conditioning

Contextual Fear Conditioning. Studies were conducted using conditioning chambers in sound attenuating cubicles equipped with a stainless steel grid floor for shock delivery and a video camera for recording freezing behavior (Med Associates, Fairfax, Vermont). Rats were acclimated to the apparatus for 3 days (10 minutes each day) prior to conditioning. On the training (conditioning) day, rats were pretreated by oral gavage with Vehicle (10% Tween 80) or Compound X (VU6080519) (0.003, 0.03, 0.1, 0.3, or 1 mg/kg) 60 minutes before being placed in the conditioning chamber that contained 1 mL 10% vanilla extract as odor cue.[2] After a 180-sec

habituation period in the chamber, a tone was presented (30s tone,3kHz, 80db) that co-terminated with a single foot shock (1 second, 0.5 mA). Rats were left in the chamber for an additional 129 before being returned to their home cages. Approximately 24 h after conditioning, rats were returned to the identical conditioning chamber and odor cue for a 4-min session to assess freezing behavior, i.e., motionless posture, excluding respiratory movements, in the absence of foot shock using the automated conditioned freezing analysis software (Med Associates).

General Methods

All NMR spectra were recorded on a 400 MHz AMX Bruker NMR spectrometer. ^{1}H and ^{13}C chemical shifts are reported in δ values in ppm downfield with the deuterated solvent as the internal standard. Data are reported as follows: chemical shift, multiplicity (s = singlet, d = doublet, t = triplet, q = quartet, b = broad, m = multiplet), integration, coupling constant (Hz). Low resolution mass spectra were obtained on an Agilent 6120/6150 or Waters QDa (Performance) SQ MS with ESI source. *Method A (Agilent 6120/6150):* MS parameters were as follows: fragmentor: 70, capillary voltage: 3000 V, nebulizer pressure: 30 psig, drying gas flow: 13 L/min, drying gas temperature: 350 ºC. Samples were introduced via an Agilent 1290 UHPLC comprised of a G4220A binary pump, G4226A ALS, G1316C TCC, and G4212A DAD with ULD flow cell. UV absorption was generally observed at 215 nm and 254 nm with a 4 nm bandwidth. Column: Waters Acquity BEH C18, 1.0 x 50 mm, 1.7 um. Gradient conditions: 5% to 95% CH_3CN in H_2O (0.1% TFA) over 1.4 min, hold at 95% CH_3CN for 0.1 min, 0.5 mL/min, 55 ºC. *Method B (Agilent 6120/6150):* MS parameters were as follows: fragmentor: 100, capillary voltage: 3000 V, nebulizer pressure: 40 psig, drying gas flow: 11 L/min, drying gas temperature: 350 ºC. Samples were introduced via an Agilent 1200 HPLC comprised of a degasser, G1312A binary pump, G1367B HP-ALS, G1316A TCC, G1315D DAD, and a Varian 380 ELSD (if applicable). UV absorption was generally observed at 215 nm and 254 nm with a 4 nm bandwidth. Column: Thermo Accucore C18, 2.1 x 30 mm, 2.6 um. Gradient conditions: 7% to 95% CH_3CN in H_2O (0.1% TFA) over 1.6 min, hold at 95% CH_3CN for 0.35 min, 1.5 mL/min, 45 ºC. *Method C (Waters QDa (Performance) SQ MS)*: MS parameters were as follows: cone voltage: 15 V, capillary voltage: 0.8 kV, probe temperature: 600° C. Samples were introduced via an Acquity I-Class PLUS UPLC comprised

of a BSM, FL-SM, CH-A, and PDA. UV absorption was generally observed at 215 nm and 254 nm; 4 nm bandwidth. Column: Phenomenex EVO C18, 1.0 x 50 mm, 1.7 um. Column temperature: 55° C. Flow rate: 0.4 mL/min. Default gradient: 5% to 95% CH_3CN (0.05% TFA) in H_2O (0.05% TFA) over 1.4 min (curve 6), hold at 95% CH_3CN for 0.1 min. "Polar" (2% to 70% CH_3CN (0.05% TFA) in H_2O (0.05% TFA) over 0.8 min (curve 6), transition to 95% CH_3CN over 0.1 min (curve 6), hold at 95% CH_3CN for 0.6 min.) and "Non-Polar" (40% to 95% CH_3CN (0.05% TFA) in H_2O (0.05% TFA) over 1.4 min (curve 6), hold at 95% CH_3CN for 0.1 min.) gradients were also available. *Method D (Waters QDa (Performance) SQ MS):* MS parameters were as follows: cone voltage: 15 V, capillary voltage: 0.8 kV, probe temperature: 600° C. Samples were introduced via an Acquity I-Class PLUS UPLC comprised of a BSM, FL-SM, CH-A, and PDA. UV absorption was generally observed at 215 nm and 254 nm with a 4 nm bandwidth. Column: Phenomenex EVO C18, 1.0 x 50 mm, 1.7 um. Column temperature: 55° C. Flow rate: 0.4 mL/min. Default gradient: 5% to 95% CH_3CN in H_2O (5 mM NH_4HCO_3) over 1.4 min (curve 6), hold at 95% CH_3CN for 0.1 min. "Polar" (2% to 70% CH_3CN in H_2O (5 mM NH_4HCO_3) over 0.8 min (curve 6), transition to 95% CH_3CN over 0.1 min (curve 6), hold at 95% CH_3CN for 0.6 min.) and "Non-Polar" (40% to 95% CH_3CN in H_2O (5 mM NH_4HCO_3) over 1.4 min (curve 6), hold at 95% CH_3CN for 0.1 min.) gradients were also available. High resolution mass spectra were obtained on an Agilent 6540 UHD Q-TOF with ESI source. MS parameters were as follows: fragmentor: 150, capillary voltage: 3500 V, nebulizer pressure: 60 psig, drying gas flow: 13 L/min, drying gas temperature: 275 ºC. Samples were introduced via an Agilent 1200 UHPLC comprised of a G4220A binary pump, G4226A 3 ALS, G1316C TCC, and G4212A DAD with ULD flow cell. UV absorption was observed at 215 nm and 254 nm with a 4 nm bandwidth. Column: Agilent Zorbax Extend C18, 1.8 µm, 2.1 x 50 mm. Gradient conditions: 5% to 95% CH_3CN in H_2O (0.1% formic acid) over 1 min, hold at 95% CH_3CN for 0.1 min, 0.5 mL/min, 40 ºC. For compounds that were purified on a Gilson preparative reversed-phase HPLC, the system comprised of a 333 aqueous pump with solvent selection valve, 334 organic pump, GX 271 or GX-281 liquid hander, two column switching valves, and a 155 UV detector. UV wavelength for fraction collection was user-defined, with absorbance at 254 nm always monitored. Method 1: Phenomenex Axia-packed Luna C18, 30 x 50 mm, 5 µm column. Mobile phase: CH_3CN in H_2O (0.1% TFA). Gradient conditions: 0.75 min equilibration, followed by user defined gradient (starting organic percentage, ending organic percentage, duration), hold at 95% CH_3CN in H_2O (0.1% TFA) for 1 min, 50

mL/min, 23 ºC. Method 2: Phenomenex Axia packed Gemini C18, 50 x 250 mm, 10 um column. Mobile phase: CH_3CN in H_2O (0.1% TFA). Gradient conditions: 7 min equilibration, followed by user defined gradient (starting organic percentage, ending organic percentage, duration), hold at 95% CH_3CN in H_2O (0.1% TFA) for 7 min, 120 mL/min, 23 ºC. Solvents for extraction, washing and chromatography were HPLC grade. All compounds are >95% purity by HPLC.

Synthetic Procedures and Spectra

Experimental Procedure for VU6052254

Commercial Reagents (in order of usage)

Name	CAS#	Supplier
methyl 2-amino-4-bromo-3-methylbenzoate	851045-38-0	Enamine
acetic anhydride	108-24-7	Sigma-Aldrich
potassium acetate	127-08-2	Sigma-Aldrich
isoamyl nitrite	110-46-3	Sigma-Aldrich
trimethyloxonium tetrafluoroborate	420-37-1	Sigma-Aldrich
cerium(III) chloride	7790-86-5	Sigma-Aldrich
methylmagnesium bromide (3.0 M in diethyl ether)	75-16-1	Sigma-Aldrich
ethyl 2-formylnicotinate	21908-07-6	Synnovator
(4-bromo-2,6-difluorophenyl)methanamine	887585-99-1	Ambeed
sodium triacetoxyborohydride	56553-60-7	Oakwood Chemicals
bis(pinocolato)diboron	73183-34-3	Combi-Blocks
[1,1'-bis(diphenylphosphino)ferrocene]dichloropalladium(II), complex with DCM	95464-05-4	Strem
cesium carbonate	534-17-8	Sigma-Aldrich

General Synthetic Scheme for the Preparation of VU6052254

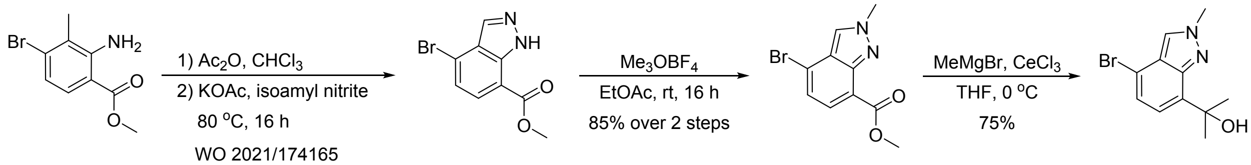

851045-38-0 **8** | **JLE-20-25** **9** | **JLE-20-26** **10** | **JLE-20-95** **11**

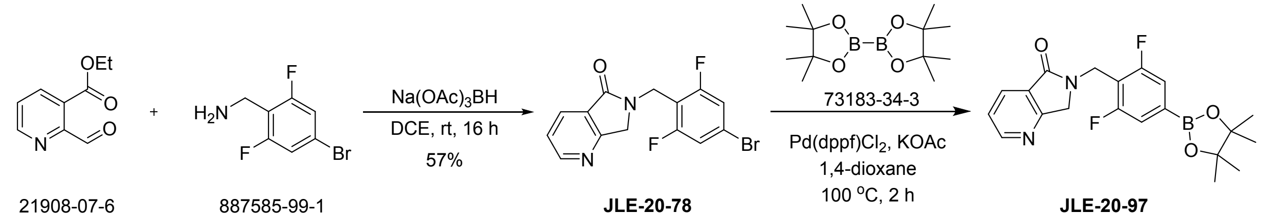

21908-07-6 **12** | 887585-99-1 **13** | **JLE-20-78** **14** | **JLE-20-97** **15**

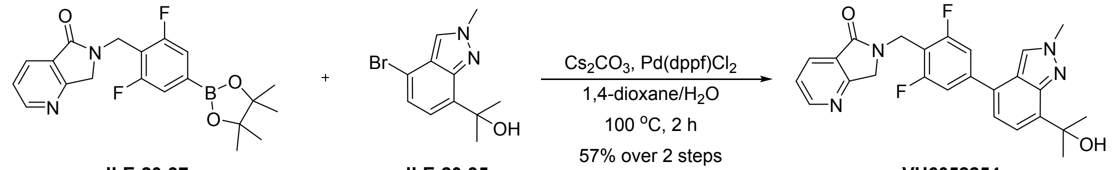

JLE-20-97 **15** | **JLE-20-95** **11** | **VU6052254** **7**

Preparation of methyl 4-bromo-1*H*-indazole-7-carboxylate (9, JLE-20-25)

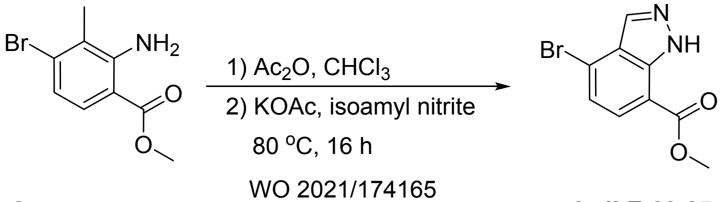

8, 851404-38-0

9, **JLE-20-25**

Similar to the procedure from WO 2021/174165: To a solution of methyl 2-amino-4-bromo-3-methylbenzoate **8** (11.0 g, 45.0 mmol, 1.0 eq.) in chloroform (225 mL, 0.2 M) was added acetic anhydride (9.8 mL, 103.5 mmol, 2.3 eq.). After 5 min, potassium acetate (1.32 g, 13.5 mmol, 0.3 eq.) was added followed by isoamyl nitrile (15.1 mL, 112.5 mmol, 2.5 eq.). The reaction mixture was stirred at 80 °C for 16 h. After cooling to rt, the resulting mixture was diluted with water (~75 mL) and extracted with DCM (3 x 250 mL). The combined extracts were washed with brine, dried over Na_2SO_4, filtered and concentrated under reduced pressure to provide the title compound **9** as a pale yellow powder (14.1 g) which was carried forward with further purification. ^{1}H NMR (400 MHz, DMSO) δ 8.22 (d, *J* = 1.5, 1H), 7.91 (dd, *J* = 7.8, 0.8 Hz, 1H), 7.51 (dd, *J* = 7.8. 0.9 Hz, 1H), 3.96 (s, 3H); ES-MS $[M+H]^+$ = 255.1/257.1.

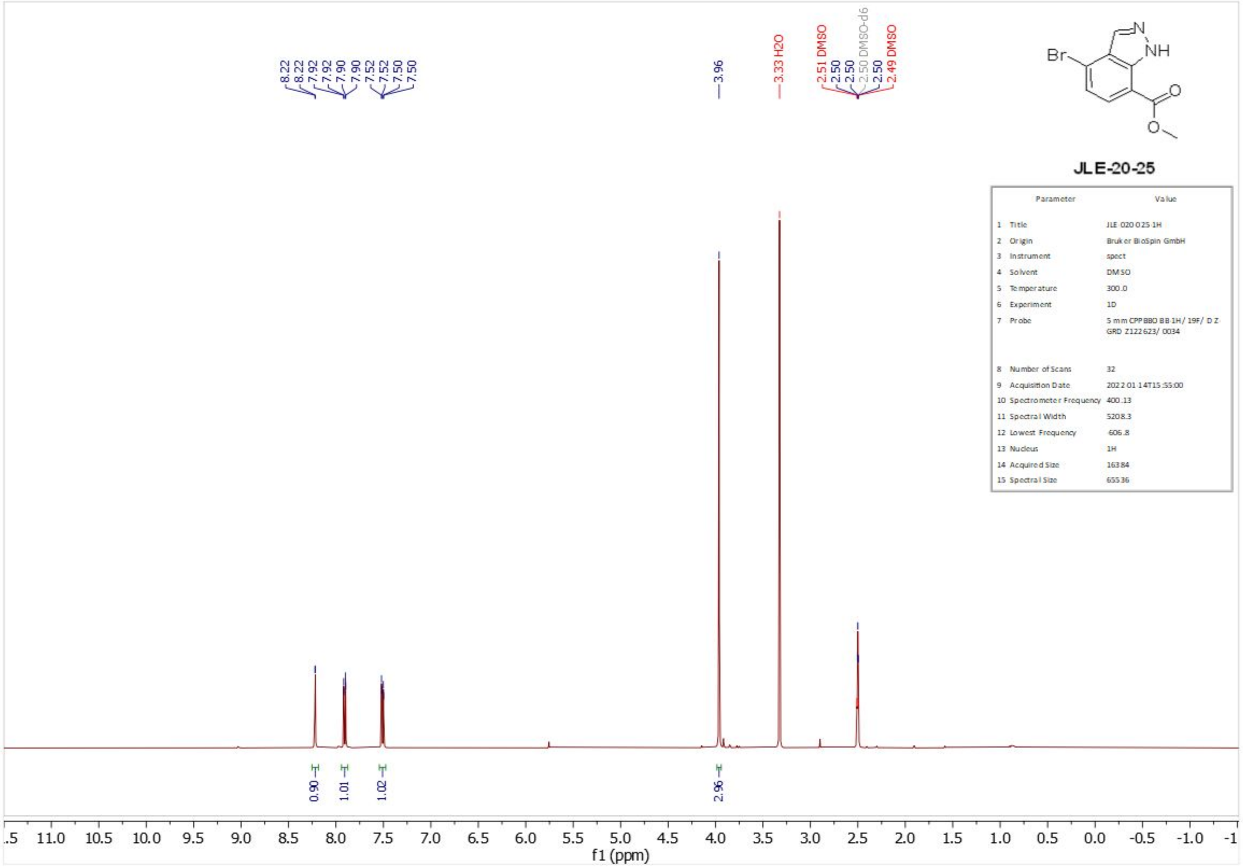

^{1}H NMR (400 MHz, DMSO) δ 8.22 (d, *J* = 1.5, 1H), 7.91 (dd, *J* = 7.8, 0.8 Hz, 1H), 7.51 (dd, *J* = 7.8. 0.9 Hz, 1H), 3.96 (s, 3H)

WCNDD CS Acquity LCMS 5 Page 1
Julie_Engers
Sample ID:JLE-20-25-FINAL Description: Vial:1:8
Date:11-Jan-2022 Time:15:49:58
Method:D:\WCNDD.PRO\OA_Methods\90-Second.olp

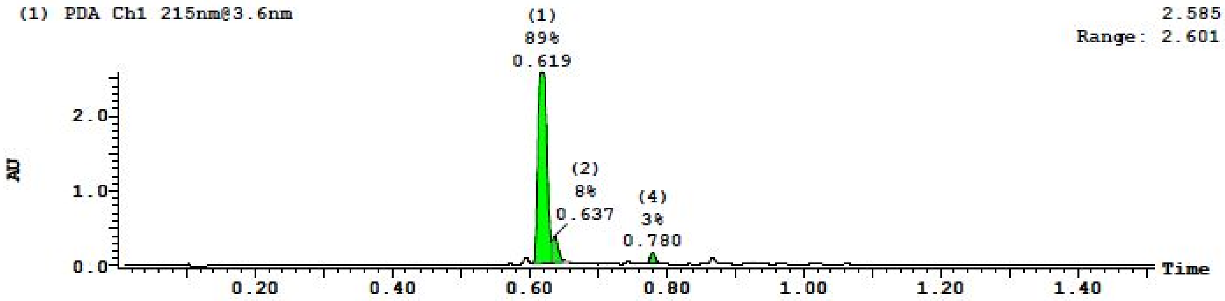

Peak Number	Time	Area %Total	BPM	Mass Found
1	0.619	89.10	255	254.0000
2	0.637	7.58	257	254.0000
4	0.780	3.33	255	254.0000

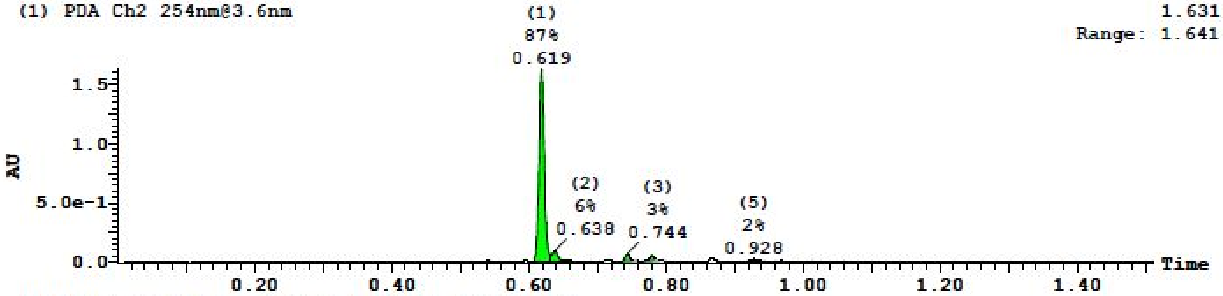

Peak Number	Time	Area %Total	BPM	Mass Found
1	0.619	86.57	255	254.0000
2	0.638	5.84	257	254.0000
3	0.744	3.22	255	254.0000
4	0.780	2.53	255	254.0000
5	0.928	1.84	476	254.0000

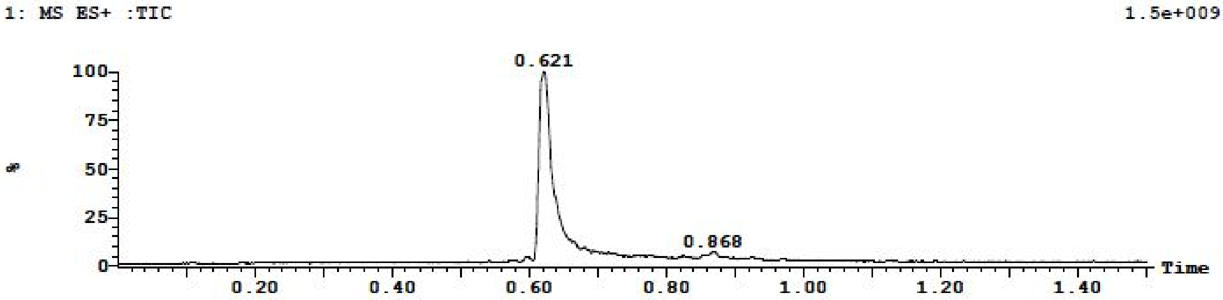

WCNDD CS Acquity LCMS 5
Julie_Engers
Sample ID:JLE-20-25-FINAL Description: Vial:1:8
Date:11-Jan-2022 Time:15:49:58
Method:D:\WCNDD.PRO\OA_Methods\90-Second.olp

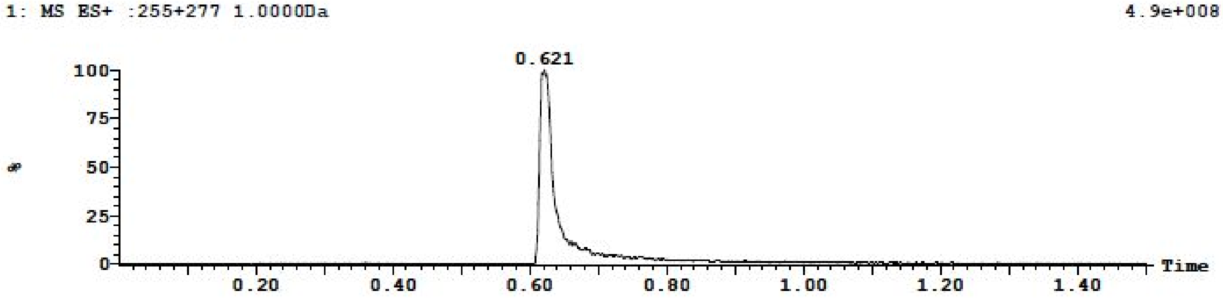

Peak ID	Time
1	0.619

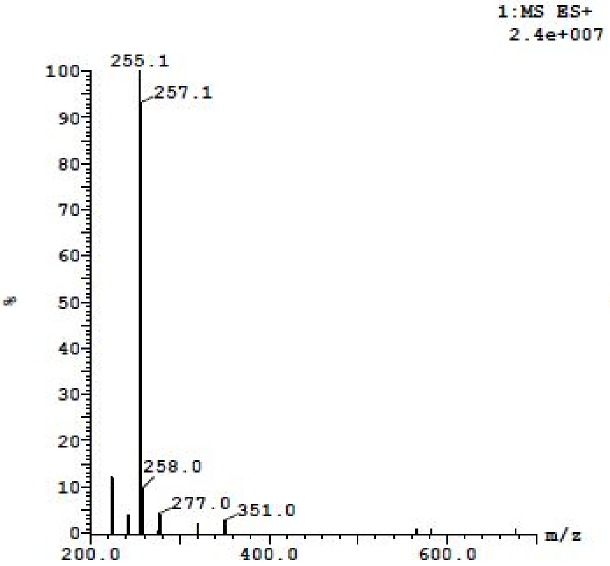

Peak ID	Time
2	0.637

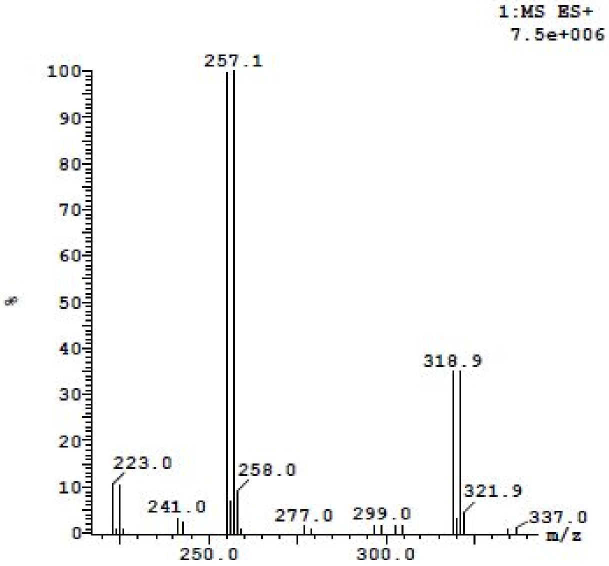

Preparation of methyl 4-bromo-2-methyl-2*H*-indazole-7-carboxylate (10, JLE-20-26)

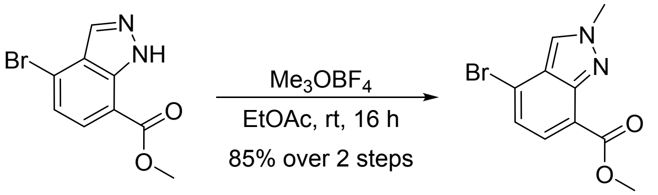

9, **JLE-20-25** **10**, **JLE-20-26**

To a solution of methyl 4-bromo-1*H*-indazole-7-carboxylate (**9**, **JLE-20-25**, 14.1 g, 45.0 mmol, 1.0 eq) in anhydrous ethyl acetate (300 mL, 0.15 M) was added trimethyloxonium tetrafluoroborate (11.3 g, 76.45 mmol, 1.5 eq.). The resulting mixture was allowed to stir at rt. After 16 h, the reaction mixture was diluted with sat. soln. $NaHCO_3$ and extracted with EtOAc (3 x 200 mL). The combined extracts were washed with brine, dried over Na_2SO_4, filtered and concentrated under reduced pressure. The crude material was purified using normal phase chromatography on silica gel (Teledyne ISCO, 220G RediSep Rf column, solid loading, 0-100% EtOAc/DCM) to provide the title compound **10** as an off white powder (10.3 g, 85% yield over 2 steps). ^{1}H NMR (400 MHz, DMSO) δ 8.61 (s, 1H), 7.83 (d, *J* = 7.6 Hz, 1H), 7.40 (dd, *J* = 7.7, 0.9 Hz, 1H), 4.24 (s, 3H), 3.88 (s, 3H); ES-MS $[M+H]^+$ = 269.0/270.9.

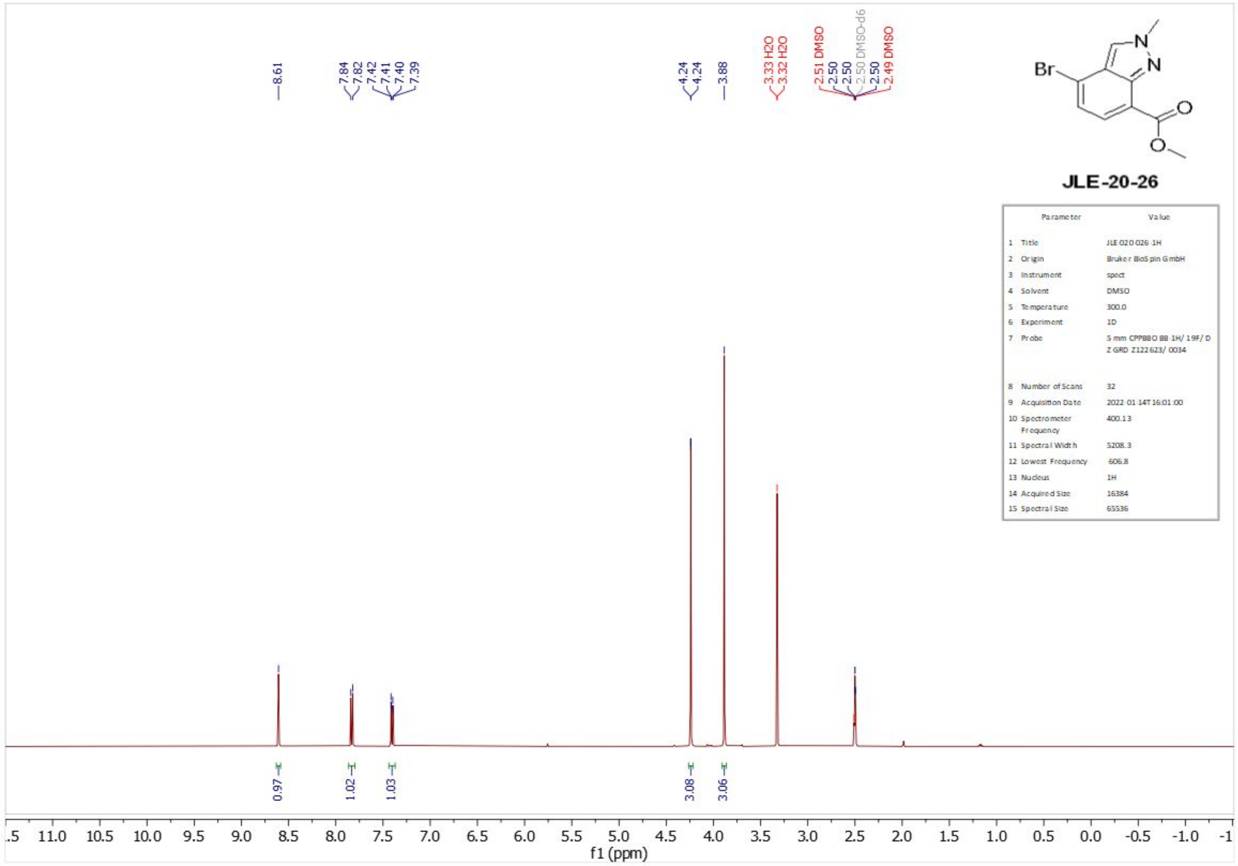

^{1}H NMR (400 MHz, DMSO) δ 8.61 (s, 1H), 7.83 (d, *J* = 7.6 Hz, 1H), 7.40 (dd, *J* = 7.7, 0.9 Hz, 1H), 4.24 (s, 3H), 3.88 (s, 3H)

WCNDD CS Acquity LCMS 5 Page 1
Julie_Engers
Sample ID:JLE-20-26-RXN Description: Vial:1:34
Date:12-Jan-2022 Time:08:08:12
Method:D:\WCNDD.PRO\OA_Methods\90-Second.olp

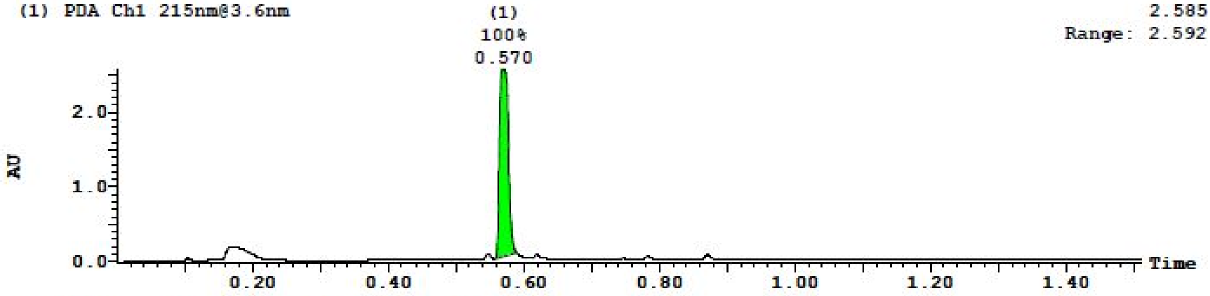

Peak Number	Time	Area %Total	BPM	Mass Found
1	0.570	100.00	271	268.0000

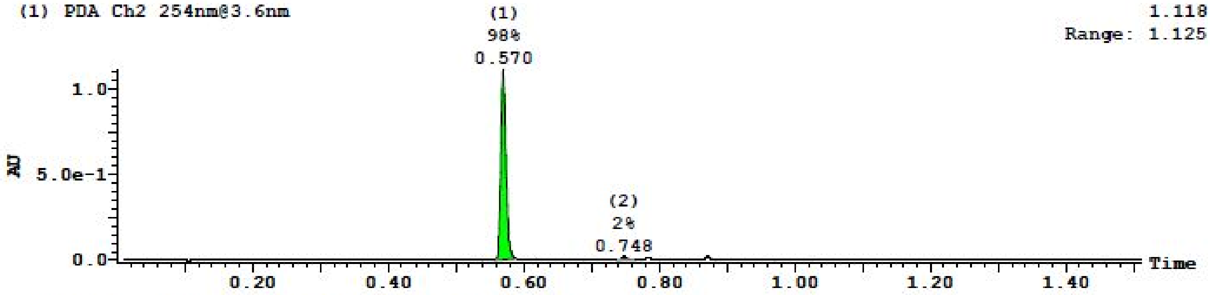

Peak Number	Time	Area %Total	BPM	Mass Found
1	0.570	97.97	271	268.0000
2	0.748	2.03	279	268.0000

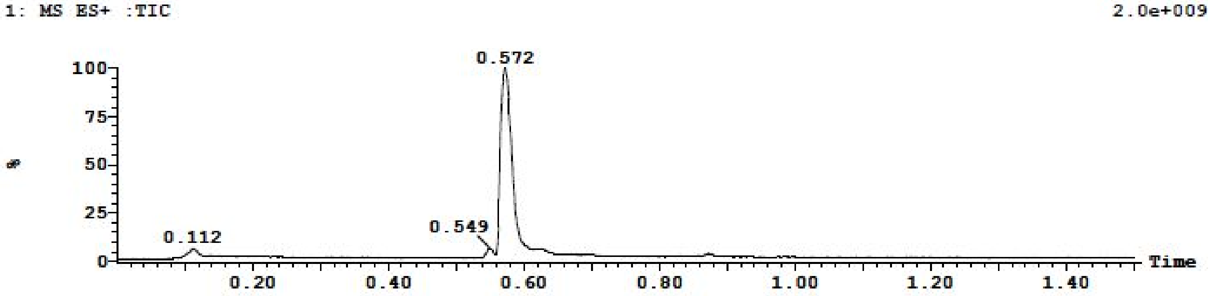

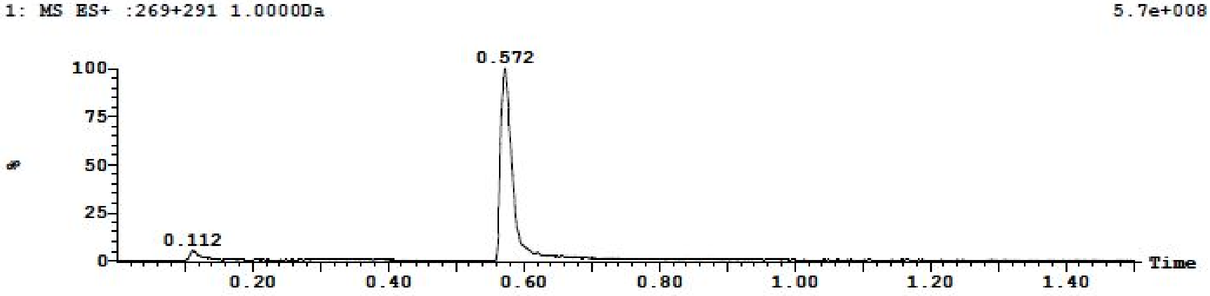

WCNDD CS Acquity LCMS 5
Julie_Engers
Sample ID:JLE-20-26-RXN
Date:12-Jan-2022
Method:D:\WCNDD.PRO\OA_Methods\90-Second.olp

Description:
Time:08:08:12

Vial:1:34

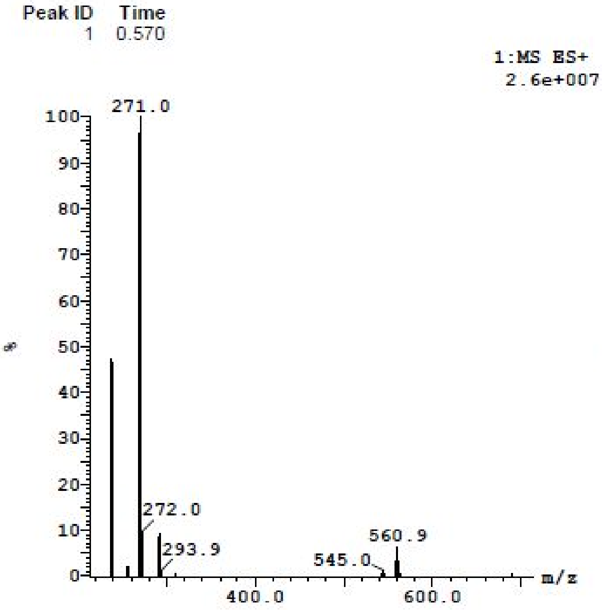

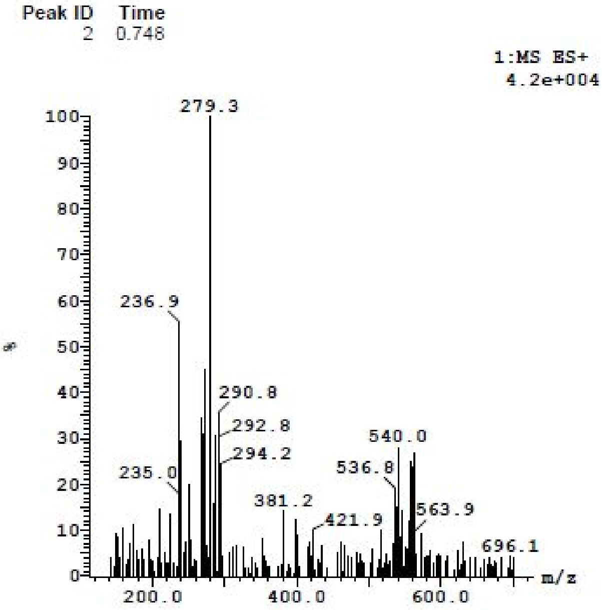

WCNDD CS Acquity LCMS 5 Page 1
Julie_Engers
Sample ID:JLE-20-26-FINAL Description: Vial:1:21
Date:14-Jan-2022 Time:14:01:07
Method:D:\WCNDD.PRO\OA_Methods\90-Second.olp

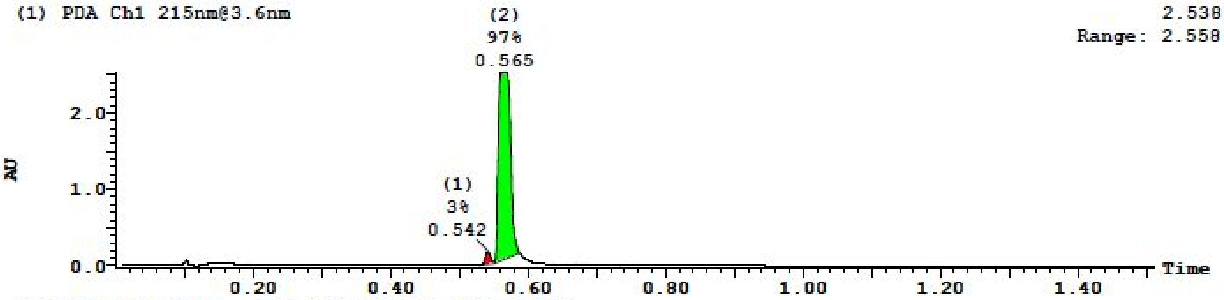

Peak Number	Time	Area %Total	BPM	Mass Found
1	0.542	2.77	193	Not Found
2	0.565	97.23	271	268.0000

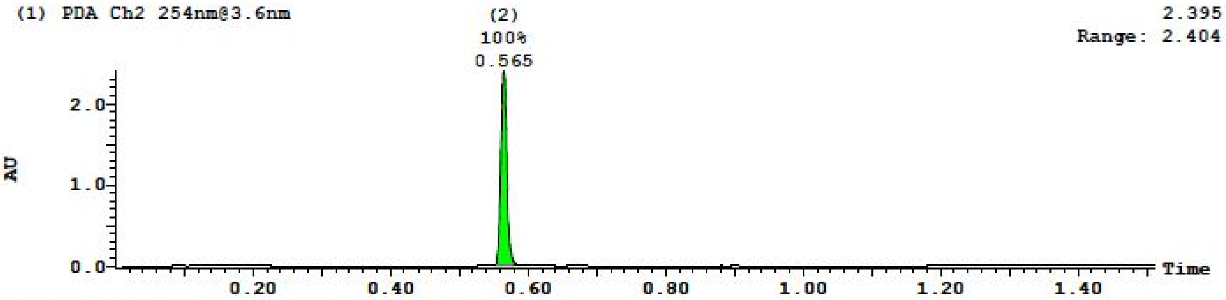

Peak Number	Time	Area %Total	BPM	Mass Found
2	0.565	100.00	271	268.0000

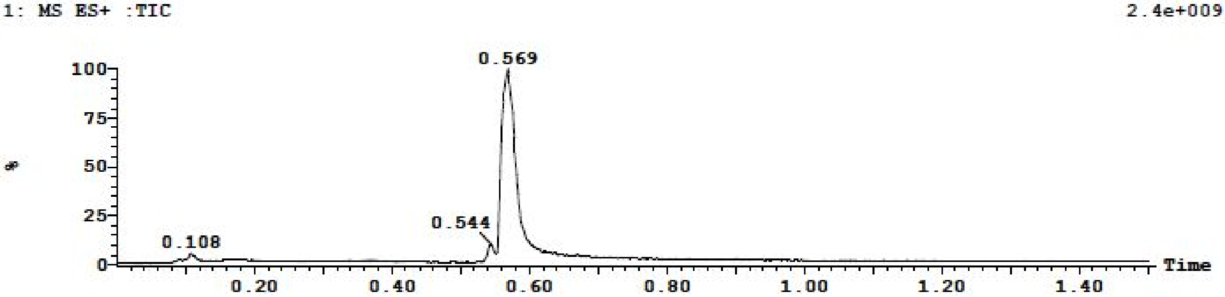

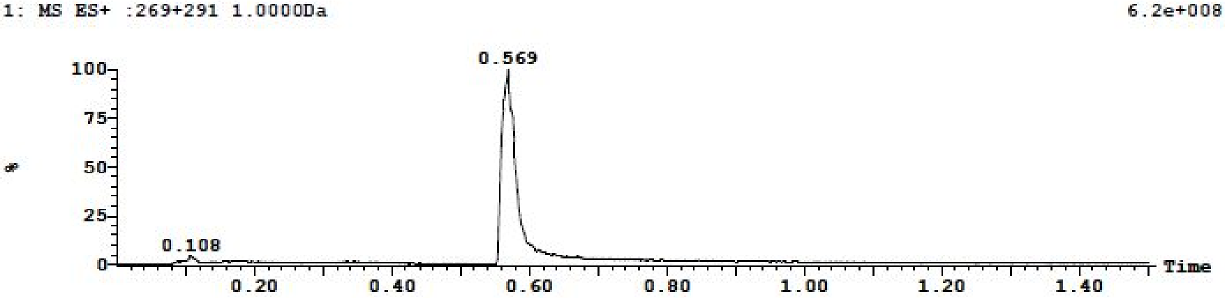

WCNDD CS Acquity LCMS 5
Julie_Engers
Sample ID:JLE-20-26-FINAL   Description: Vial:1:21
Date:14-Jan-2022   Time:14:01:07
Method:D:\WCNDD.PRO\OA_Methods\90-Second.olp

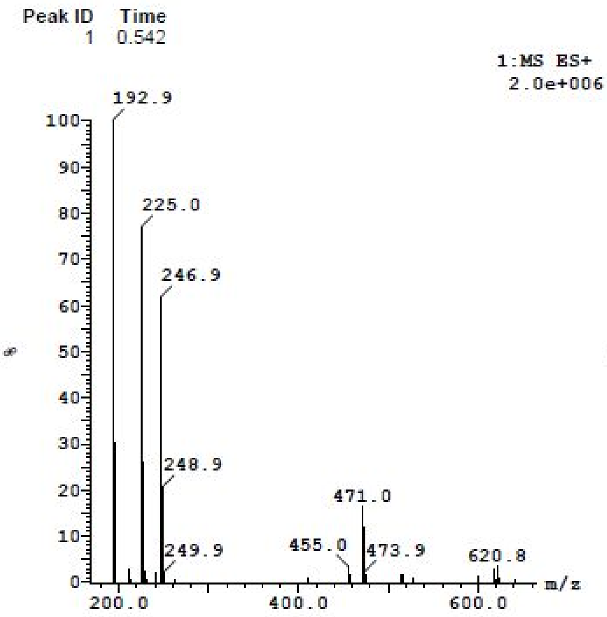

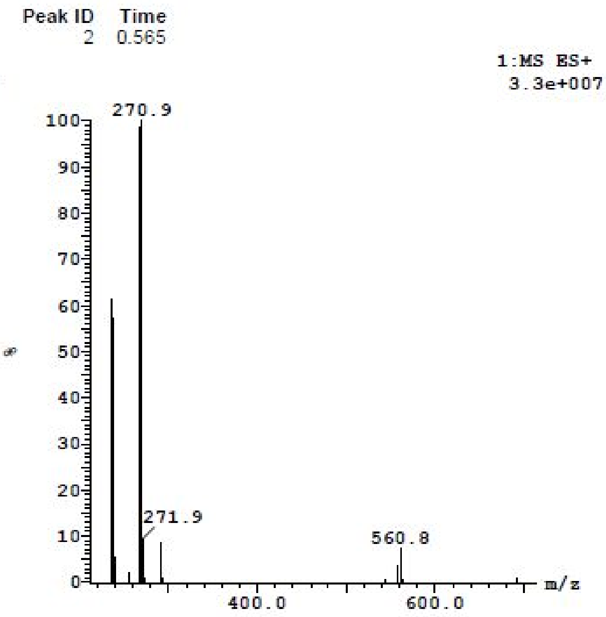

Preparation of 2-(4-bromo-2-methyl-2*H*-indazol-7-yl)propan-2-ol (11, JLE-20-95)

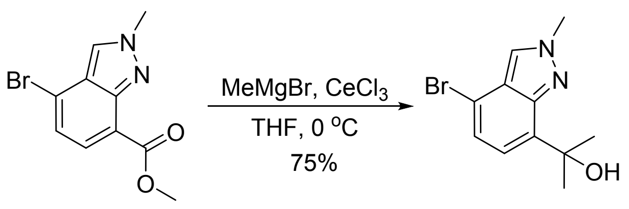

10, **JLE-20-26** **11**, **JLE-20-95**

In an oven-dried 500 mL RBF was charged with cerium(III) chloride (20.0 g, 80.0 mmol, 4.0 eq) and THF (100 mL). The reaction mixture was heated to 50 °C for 30 min then cooled to 0 °C and a solution of methylmagnesium bromide (3.0 M in Et_2O, 26.67 mL, 80.0 mmol, 4.0 eq.) was added. After 15 min, a solution of methyl 4-bromo-2-methyl-2*H*-indazole-7-carboxylate (**10, JLE-20-26**, 5.38 g, 20.0 mmol, 1.0 eq) in THF (45 mL) was added dropwise. After 1 h, sat. soln. NH_4Cl (~100 mL) was added slowly. The resulting mixture was extracted with EtOAc (3 x 150 mL). The combined extracts were washed with brine, dried over Na_2SO_4, filtered and concentrated under reduced pressure. Purification using normal phase chromatography on silica gel (Teledyne ISCO, 120G RediSep Rf column, solid loading, 0-100% EtOAc/hexanes) to provide the title compound **11** as an off white powder (4.05 g, 75% yield). ^{1}H NMR (400 MHz, DMSO) δ 8.36 (s, 1H), 7.27 (d, *J* = 7.5 Hz, 1H), 7.21 (d, *J* = 7.5, 1H), 5.15 (s, 1H), 4.19 (s, 3H), 1.65 (s, 6H); ^{13}C NMR (101 MHz, DMSO) δ 145.47, 138.55, 125.16, 123.56, 123.36, 121.18, 109.89, 70.86, 40.29, 40.15, 30.28; ES-MS $[M-H_2O]^+$ = 252.9; HRMS (ESI/Q-TOF) m/z: $[M+H]^+$ Calcd for $C_{11}H_{13}BrN_2O$ 269.0284, Found 269.0280.

*Note: to avoid the formation of the debromination byproduct (ES-MS $[M-H_2O]^+$ = 187.1), reduce the equivalents of cerium(III) chloride and methylmagnesium bromide to 2.5 – 3.0 and slower addition rate.

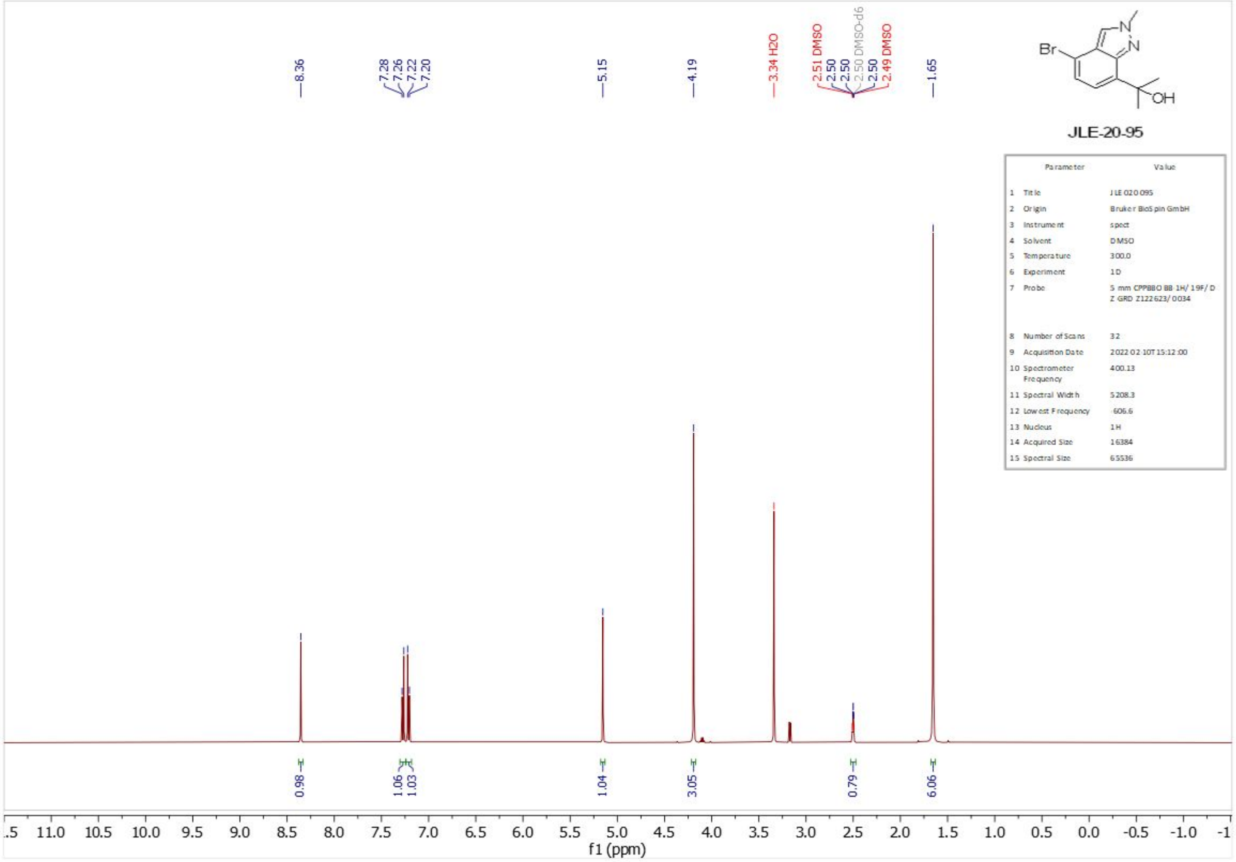

^{1}H NMR (400 MHz, DMSO) δ 8.36 (s, 1H), 7.27 (d, *J* = 7.5 Hz, 1H), 7.21 (d, *J* = 7.5, 1H), 5.15 (s, 1H), 4.19 (s, 3H), 1.65 (s, 6H)

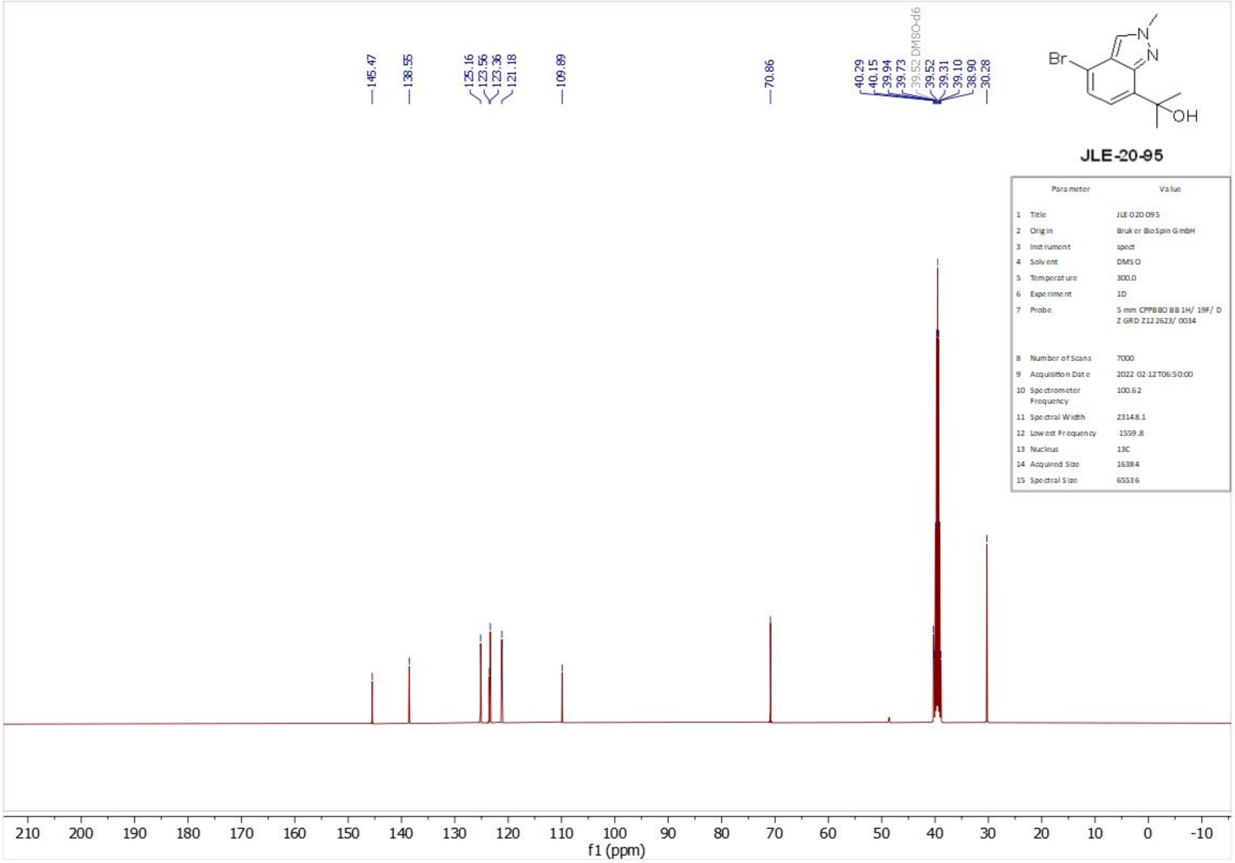

^{13}C NMR (101 MHz, DMSO) δ 145.47, 138.55, 125.16, 123.56, 123.36, 121.18, 109.89, 70.86, 40.29, 40.15, 30.28.

WCNDD CS Acquity LCMS 4

Julie_Engers

Sample ID:JLE-20-95-RXN-1H-OC | Description: | Vial:1:41
Date:09-Feb-2022 | Time:13:49:56
Method:D:\WCNDD.PRO\OA_Methods\90-Second.olp

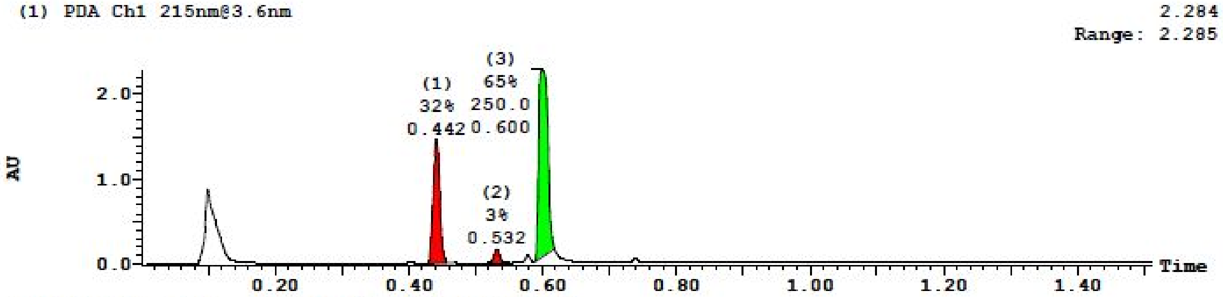

Peak Number	Time	Area %Total	BPM	Mass Found
1	0.442	32.13	187	Not Found
2	0.532	3.17	255	Not Found
3	0.600	64.71	253	250.0000

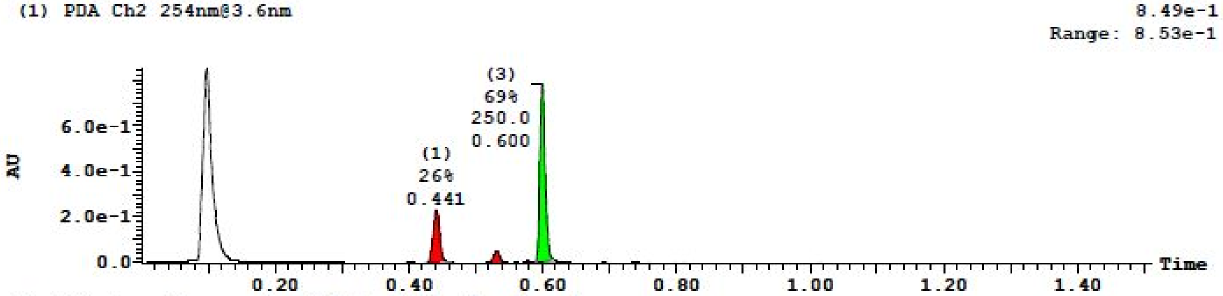

Peak Number	Time	Area %Total	BPM	Mass Found
1	0.441	25.50	187	Not Found
2	0.532	5.38	255	Not Found
3	0.600	69.12	253	250.0000

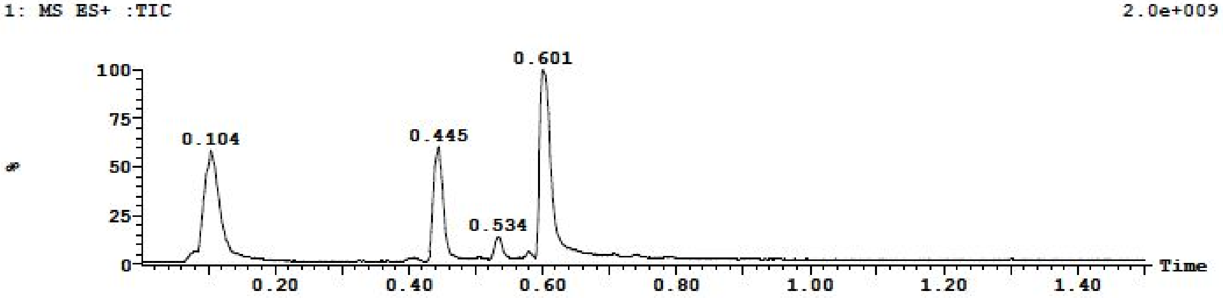

Julie_Engers
Sample ID:JLE-20-95-RXN-1H-OC Description: Vial:1:41
Date:09-Feb-2022 Time:13:49:56
Method:D:\WCNDD.PRO\OA_Methods\90-Second.olp

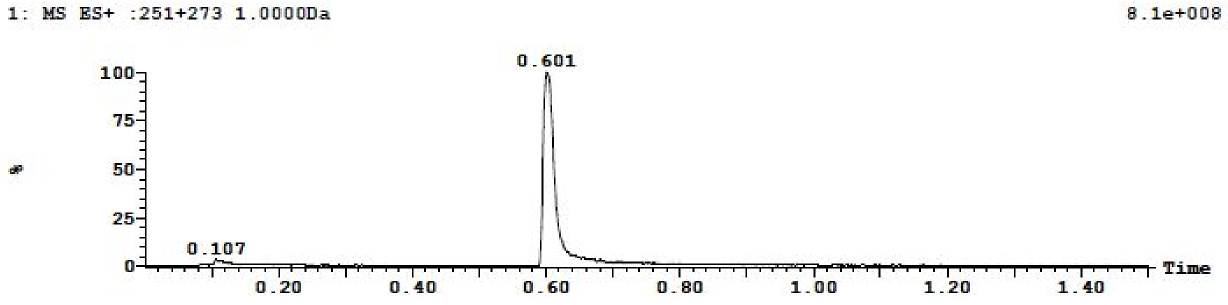

Peak ID	Time
1	0.442

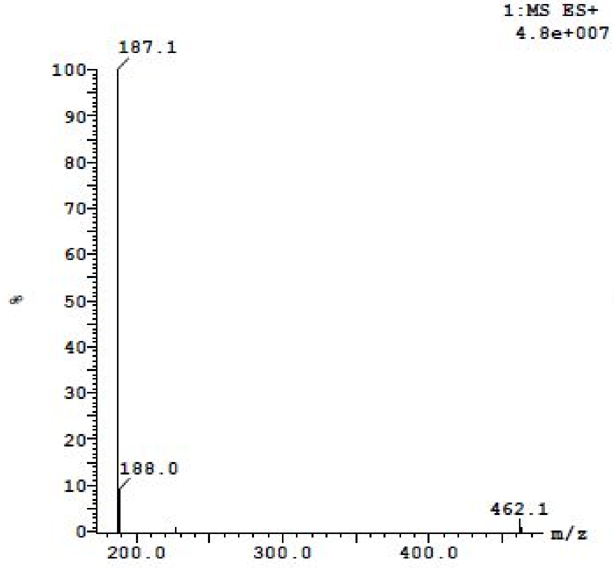

Peak ID	Time
2	0.532

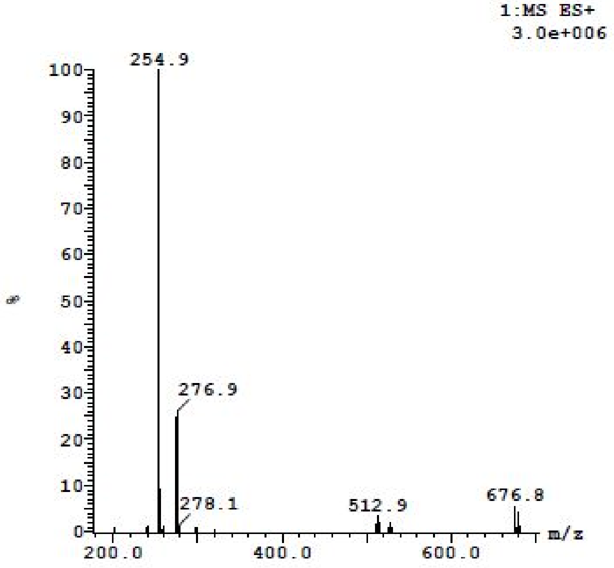

WCNDD CS Acquity LCMS 4 Page 1
Julie_Engers
Sample ID:JLE-20-95-FINAL Description: Vial:1:34
Date:10-Feb-2022 Time:14:39:26
Method:D:\WCNDD.PRO\OA_Methods\90-Second.olp

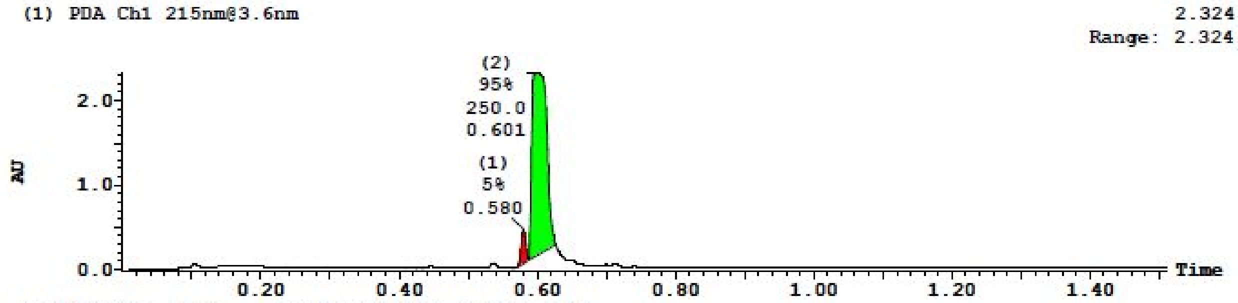

Peak Number	Time	Area %Total	BPM	Mass Found
1	0.580	5.33	207	Not Found
2	0.601	94.67	253	250.0000

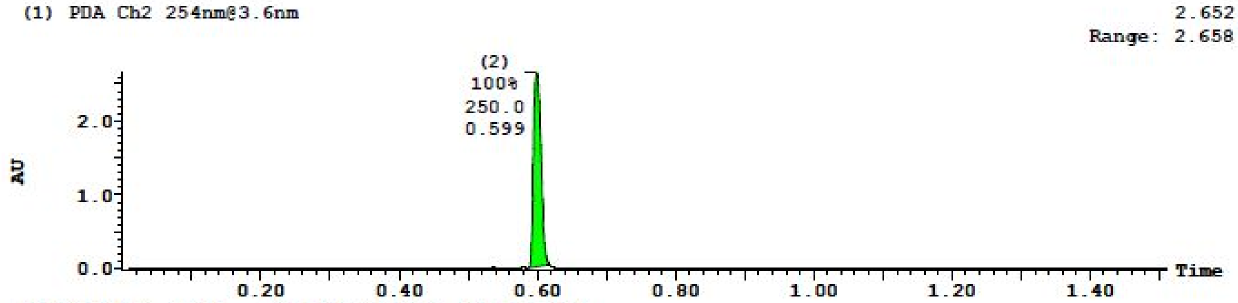

Peak Number	Time	Area %Total	BPM	Mass Found
2	0.599	100.00	253	250.0000

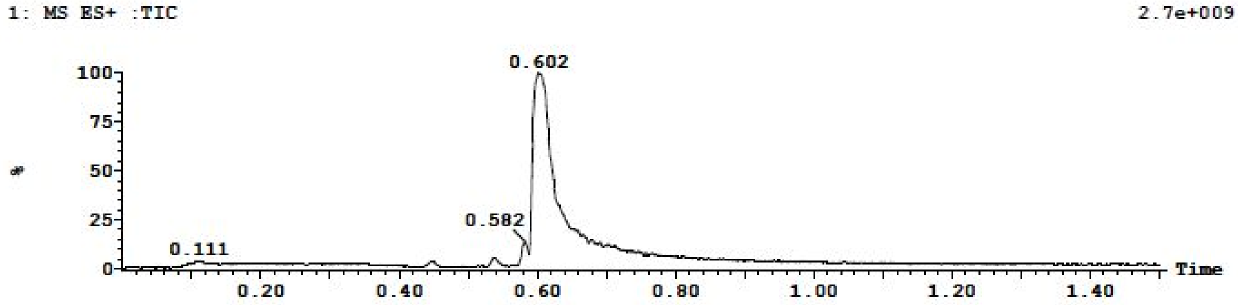

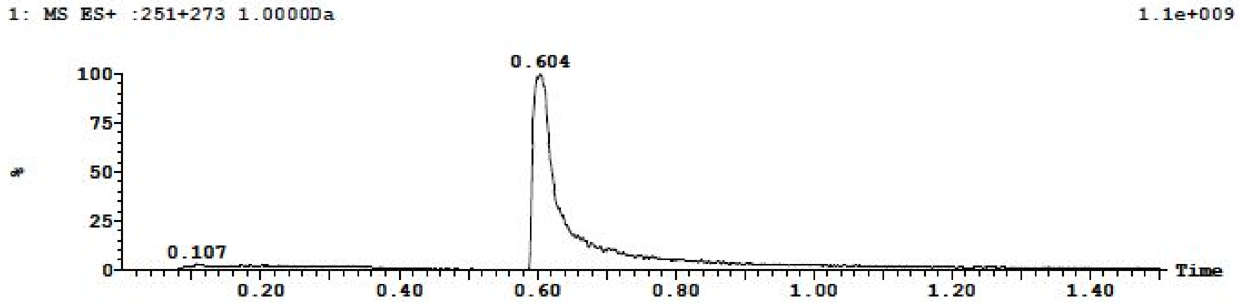

Sample ID:JLE-20-95-FINAL Description: Vial:1:34
Date:10-Feb-2022 Time:14:39:26
Method:D:\WCNDD.PRO\OA_Methods\90-Second.olp

Peak ID	Time
1	0.580

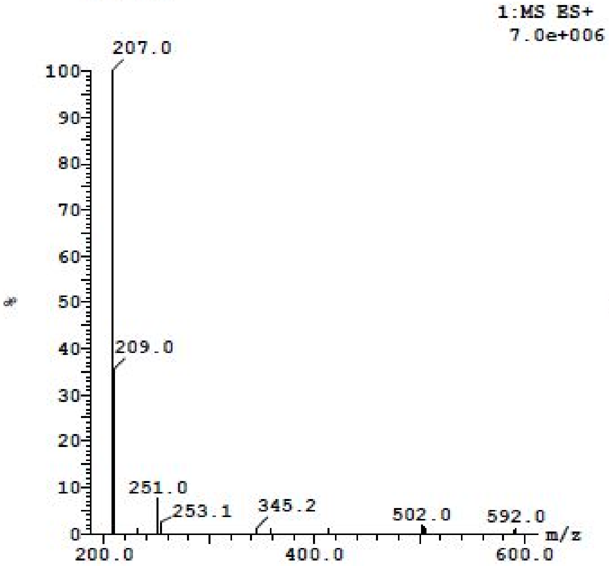

Peak ID	Time
2	0.601

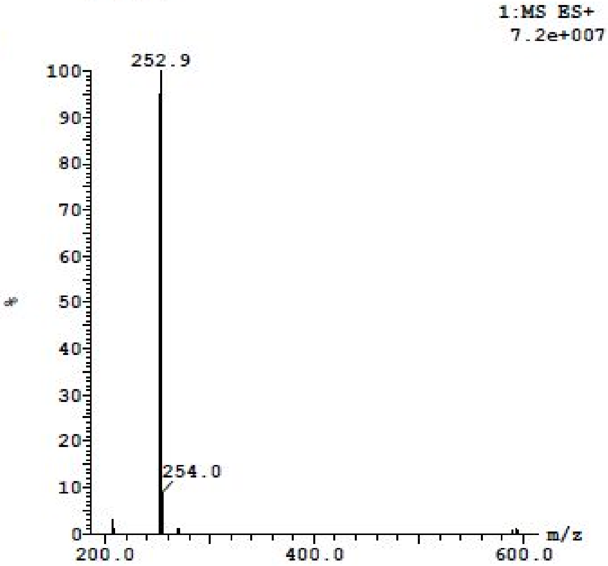

**Data File**	JLE-20-95_0003.d	**Sample Name**	JLE-20-95
**Sample Type**	Sample	**Position**	P1-E2
**Instrument Name**	Q-TOF	**User Name**	Christopher Presley
**Acq Method**	Auto MSMS_1x50_5-95_90sec.m	**Acquired Time**	7/21/2022 4:46:38 PM (UTC-05:00)
**IRM Calibration Status**	Success	**DA Method**	Default Report AMM_210224.m
**Comment**	0.1 µL Injection		

**Sample Group**		**Info.**	
**Molecular Formula**	C11H13BrN2O	**Stream Name**	LC 1
**Acquisition Time (Local)**	7/21/2022 4:46:38 PM (UTC-05:00)	**Acquisition SW Version**	6200 series TOF/6500 series Q-TOF 10.1 (48.0)
**QTOF Driver Version**	10.01.00	**QTOF Firmware Version**	10.811
**DDE Mode**	2	**Tune Mass Range Max.**	3200

Compound Table

Compound Label	RT	$Mass_{m/z\ -\ addt}$	Abund	Name	Formula	$Mass_{TGT}$	Diff (ppm)	Hits (DB)
Cpd 1: JLE-20-95; C11 H13 Br N2 O; 1.256	1.256	268.0212	3045	JLE-20-95	C11 H13 Br N2 O	268.0211	0.33	1

Compound Label	Name	m/z Observed	RT	Algorithm
Cpd 1: JLE-20-95; C11 H13 Br N2 O; 1.256	JLE-20-95	269.0280	1.256	Find by Formula

Calc m/z	Ion
269.0284	(M+H)+

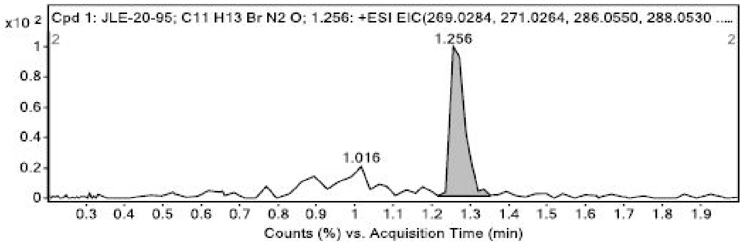

MS Zoomed Spectrum

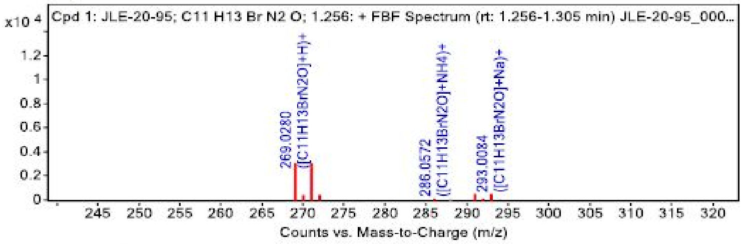

MS Spectrum Peak List

m/z	z	Abund	Formula	Ion
269.028	1	3044.93	C11H13BrN2O	(M+H)+
270.0308	1	312.06	C11H13BrN2O	(M+H)+
271.0268	1	2576.35	C11H13BrN2O	(M+H)+
272.0292	1	401.48	C11H13BrN2O	(M+H)+
286.0572	1	92.61	C11H13BrN2O	(M+NH4)+
291.0119	1	436.67	C11H13BrN2O	(M+Na)+
292.0153	1	78.99	C11H13BrN2O	(M+Na)+
293.0084	1	451.08	C11H13BrN2O	(M+Na)+

MS Zoomed Spectrum

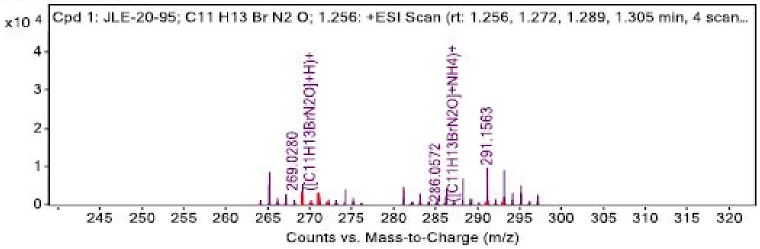

MS Spectrum Peak List

m/z	*Calc m/z*	Diff(ppm)	z	Abund	Formula	Ion
269.028	269.0284	-1.38	1	3044.93	C11H13BrN2O	(M+H)+
270.0308	270.0314	-2.3	1	312.06	C11H13BrN2O	(M+H)+
271.0268	271.0264	1.43	1	2576.35	C11H13BrN2O	(M+H)+
272.0292	272.0294	-0.83	1	401.48	C11H13BrN2O	(M+H)+
286.0572	286.055	7.94	1	92.61	C11H13BrN2O	(M+NH4)+
291.0119	291.0103	5.38	1	436.67	C11H13BrN2O	(M+Na)+
291.1563				9611.13		
292.0153	292.0134	6.62	1	78.99	C11H13BrN2O	(M+Na)+
293.0084	293.0084	0.24	1	451.08	C11H13BrN2O	(M+Na)+

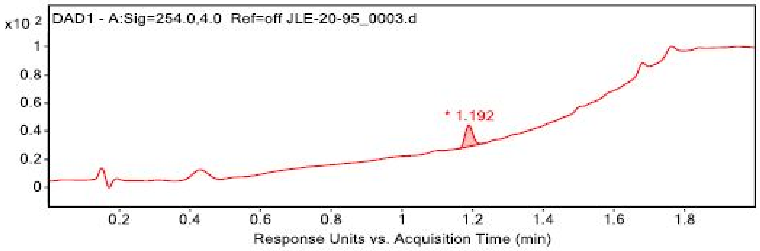

User Chromatogram Peak List

RT	Height	Normalized Height	Height %	Area	Area %	Area Sum %	Symmetry	Width
1.192	2.29	14.92	100	3.5	100	100	41.43	0.07

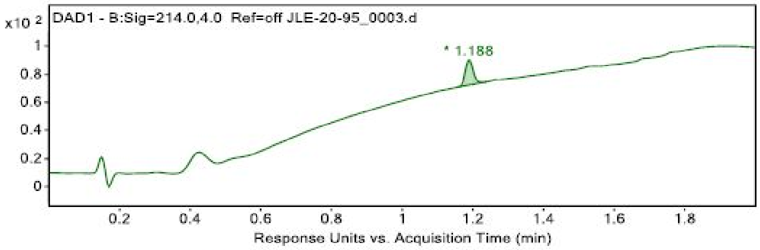

User Chromatogram Peak List

RT	Height	Normalized Height	Height %	Area	Area %	Area Sum %	Symmetry	Width
1.188	43.97	17.7	100	67.97	100	100	48	0.08

--- End Of Report ---

Preparation of 6-(4-bromo-2,6-difluorobenzyl)-6,7-dihydro-5*H*-pyrrolo[3,4-*b*]pyridin-5-one (14, JLE-20-78)

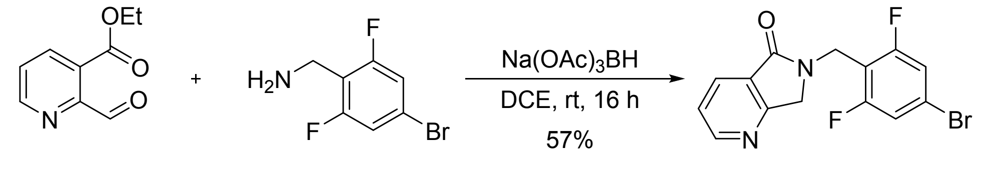

12, 21908-07-6 **13**,887585-99-1 **14**, **JLE-20-78**

In a 500 mL RBF, (4-bromo-2,6-difluorophenyl)methanamine **13** (9.77 g, 44.0 mmol, 1.1 eq.) and ethyl 2-formylnicotinate **12** (7.17 g, 40.0 mmol, 1.0 eq.) were combined and anhydrous DCE (200 mL) was added. After stirring at rt for 30 min, sodium triacetoxyborohydride (12.72 g, 60.0 mmol, 1.5 eq.) was added portionwise. The resulting mixture was stirred at rt for 16 h, diluted with DCM (300 mL) and washed with sat. soln. $NaHCO_3$ (200 mL). The aqueous layer was back-extracted with DCM (2 x 100 mL). The combined extracts were washed with brine, dried over Na_2SO_4, filtered and concentrated under reduced pressure. Purification using normal phase chromatography on silica gel (Teledyne ISCO, 220G RediSep Rf column, liquid loading, 0-50% EtOAc/hexanes then 50-100% EtOAc/DCM) to provide the title compound **14** as an off white powder (7.8 g, 57% yield). ^{1}H NMR (400 MHz, DMSO) δ 8.74 (dd, *J* = 4.9, 1.6 Hz, 1H), 8.09 (dd, *J* = 7.7, 1.6 Hz, 1H), 7.55 – 7.48 (m, 3H), 4.80 (d, *J* = 1.3 Hz, 2H), 4.43 (s, 2H); ^{13}C NMR (101 MHz, DMSO) δ 165.39, 162.31 (d, J_{C-F} = 9.2 Hz, CF), 162.22, 159.81 (d, *J* J_{C-F} = 9.2 Hz), 152.59, 131.42, 125.16, 123.40, 121.46 (t, J_{CH-C-F} = 12.8 Hz), 116.75 – 114.51 (2C, m), 112.01 (t, J_{CH-C-F} = 19.8 Hz), 50.67, 33.23 (t, $J_{CH2-C-F}$ = 3.4 Hz); ES-MS $[M+H]^+$ = 339.0/341.0; HRMS (ESI/Q-TOF) m/z: $[M+H]^+$ Calcd for $C_{14}H_9BrF_2N_2O$ 338.9939, Found 338.9942.

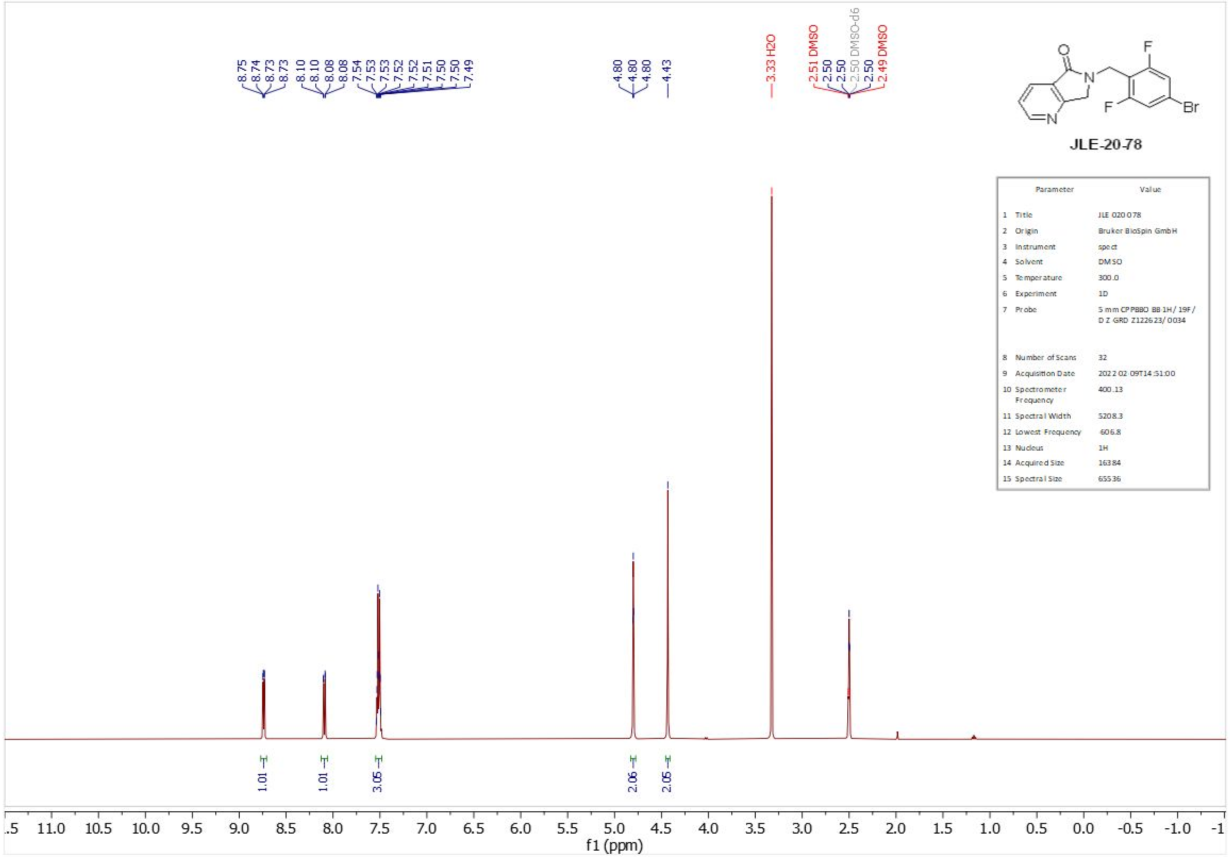

^{1}H NMR (400 MHz, DMSO) δ 8.74 (dd, *J* = 4.9, 1.6 Hz, 1H), 8.09 (dd, *J* = 7.7, 1.6 Hz, 1H), 7.55 – 7.48 (m, 3H), 4.80 (d, *J* = 1.3 Hz, 2H), 4.43 (s, 2H).

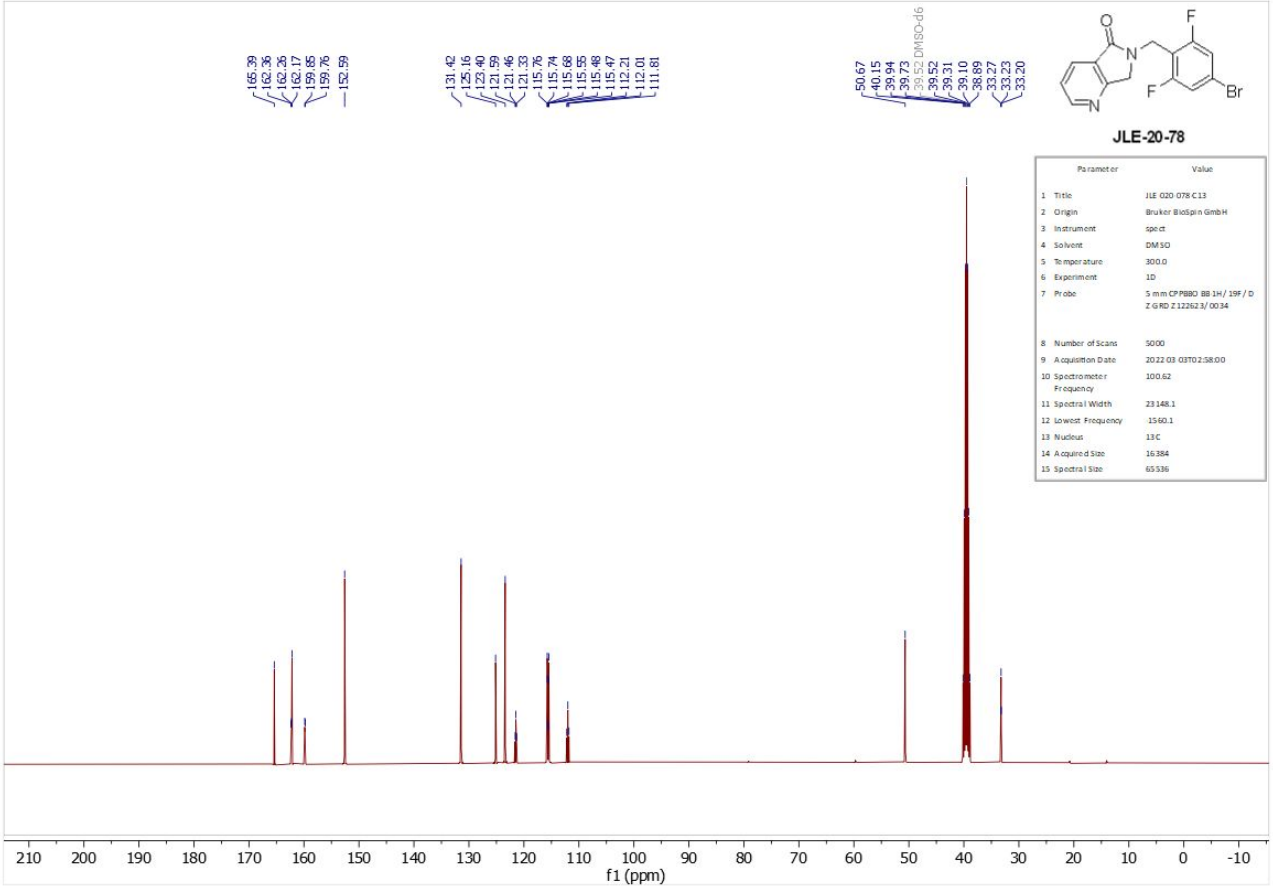

^{13}C NMR (101 MHz, DMSO) δ 165.39, 162.31 (d, $J_{C\text{-}F}$ = 9.2 Hz, CF), 162.22, 159.81 (d, *J* $J_{C\text{-}F}$ = 9.2 Hz), 152.59, 131.42, 125.16, 123.40, 121.46 (t, $J_{CH\text{-}C\text{-}F}$ = 12.8 Hz), 116.75 – 114.51 (2C, m), 112.01 (t, $J_{CH\text{-}C\text{-}F}$ = 19.8 Hz), 50.67, 33.23 (t, $J_{CH2\text{-}C\text{-}F}$ = 3.4 Hz).

WCNDD CS Acquity LCMS 4 Page 1
Julie_Engers
Sample ID:JLE-20-78-RXN Description: Vial:2:30
Date:09-Feb-2022 Time:07:54:38
Method:D:\WCNDD.PRO\OA_Methods\90-Second.olp

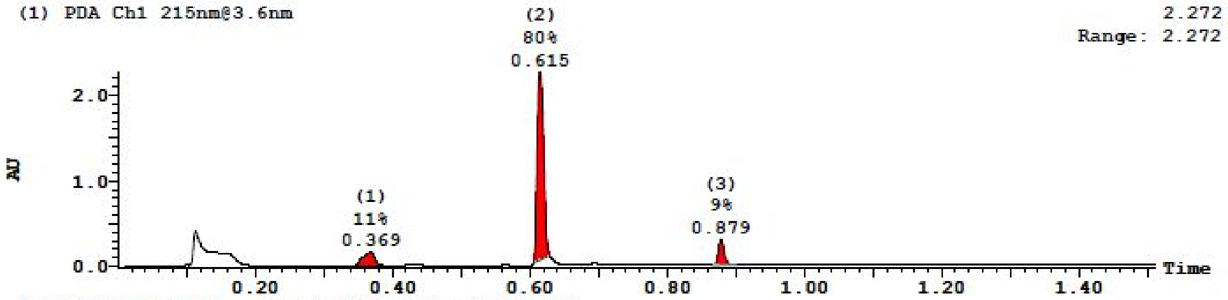

Peak Number	Time	Area %Total	BPM	Mass Found
1	0.369	10.75	359	Not Found
2	0.615	79.90	341	Not Found
3	0.879	9.35	355	Not Found

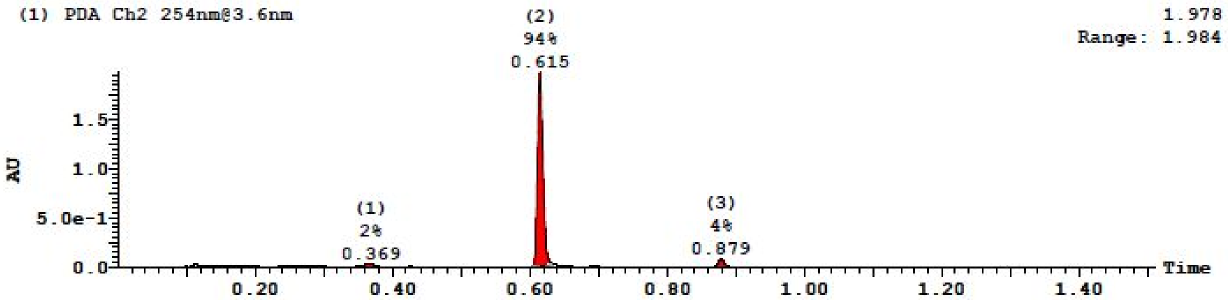

Peak Number	Time	Area %Total	BPM	Mass Found
1	0.369	2.44	359	Not Found
2	0.615	93.60	341	Not Found
3	0.879	3.96	355	Not Found

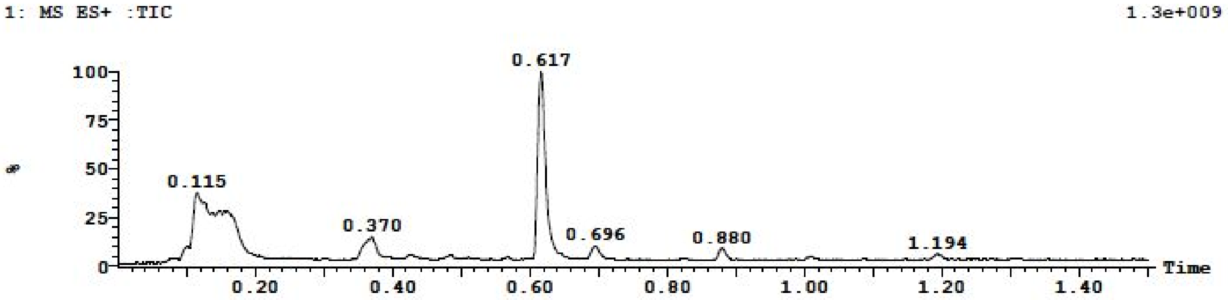

Julie_Engers
Sample ID:JLE-20-78-RXN Description: Vial:2:30
Date:09-Feb-2022 Time:07:54:38
Method:D:\WCNDD.PRO\OA_Methods\90-Second.olp

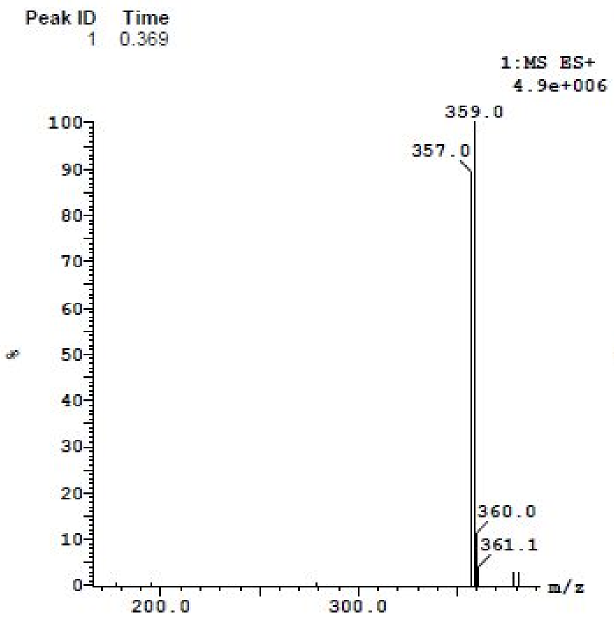

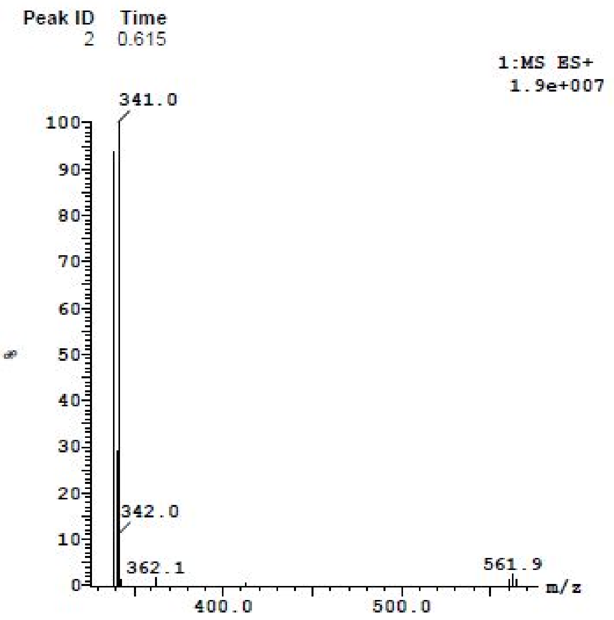

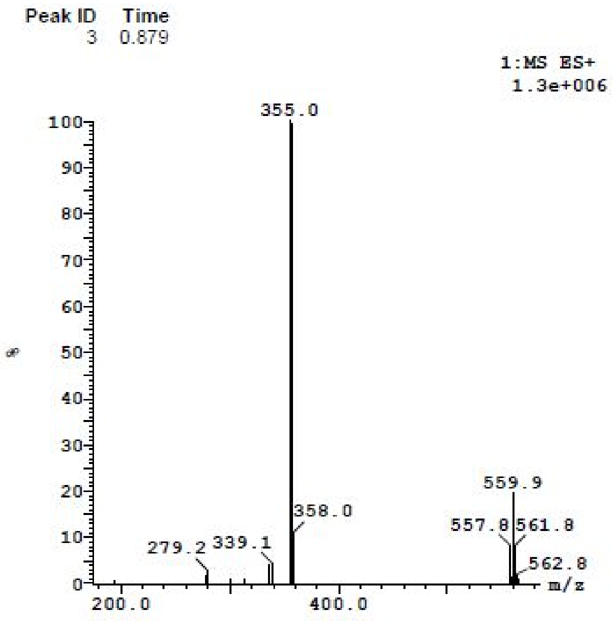

WCNDD CS Acquity LCMS 4
Julie_Engers

Sample ID:JLE-20-78-FINAL Description: Vial:1:42
Date:09-Feb-2022 Time:13:54:19
Method:D:\WCNDD.PRO\OA_Methods\90-Second.olp

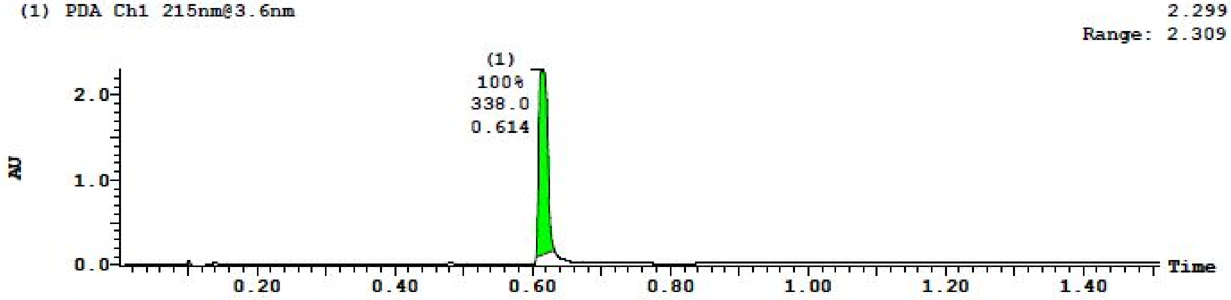

Peak Number	Time	Area %Total	BPM	Mass Found
1	0.614	100.00	341	338.0000

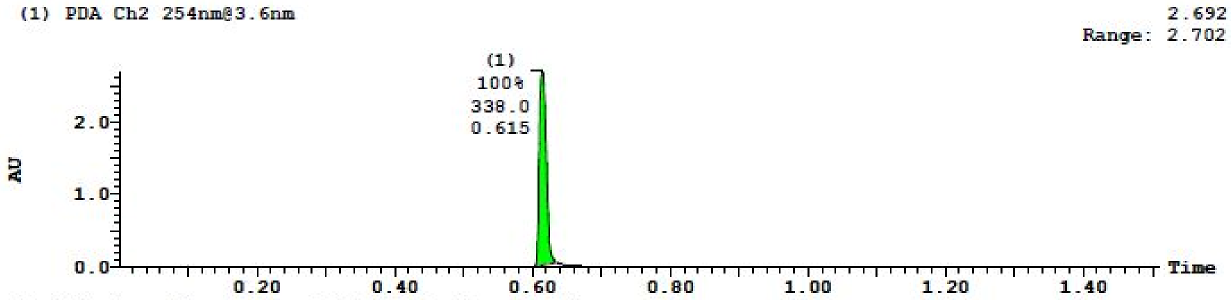

Peak Number	Time	Area %Total	BPM	Mass Found
1	0.615	100.00	341	338.0000

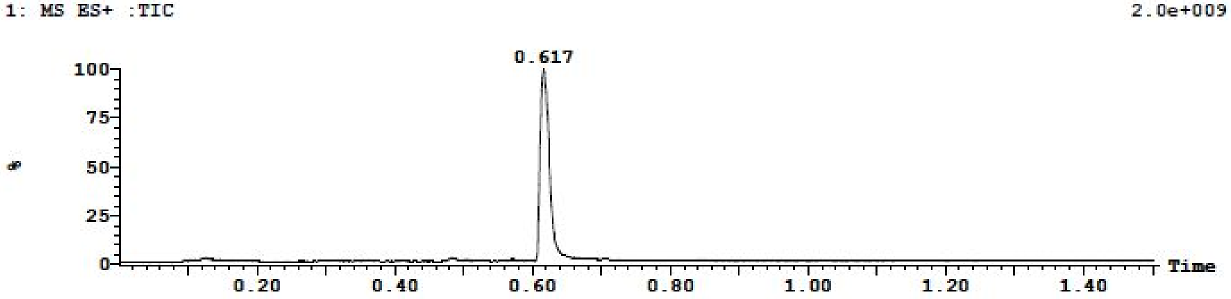

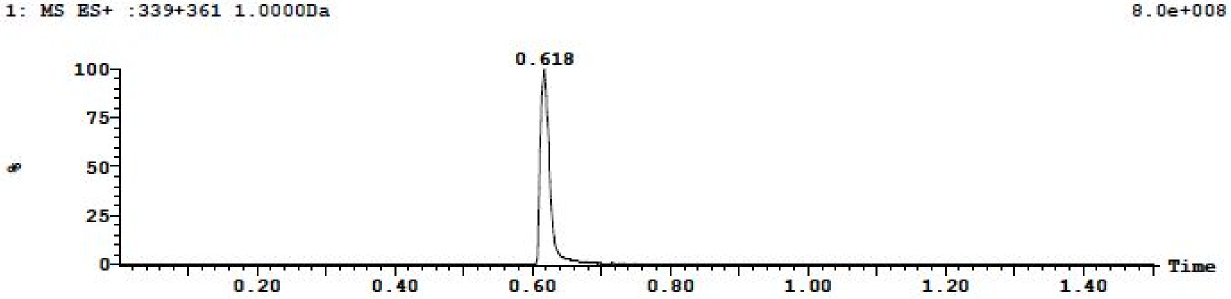

Julie_Engers

Sample ID:JLE-20-78-FINAL Description: Vial:1:42
Date:09-Feb-2022 Time:13:54:19
Method:D:\WCNDD.PRO\OA_Methods\90-Second.olp

Peak ID	Time
1	0.614

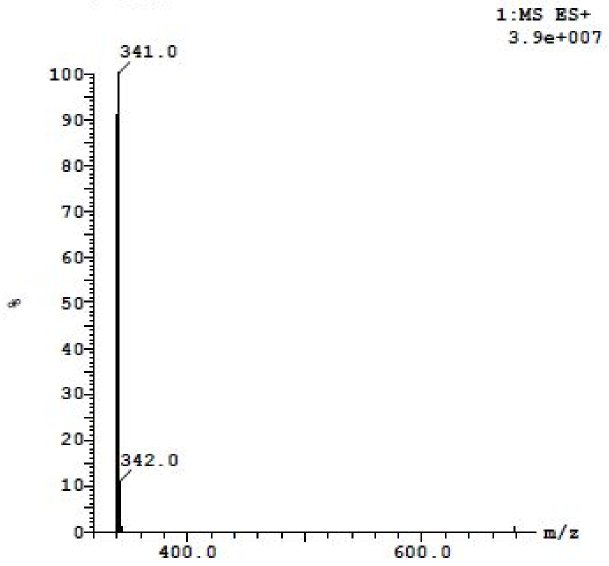

HRMS/Accurate Mass Report

WARREN CENTER FOR NEUROSCIENCE DRUG DISCOVERY at Vanderbilt University

**Data File**	JLE-20-78_0001.d	**Sample Name**	JLE-20-78
**Sample Type**	Sample	**Position**	P1-E1
**Instrument Name**	Q-TOF	**User Name**	Christopher Presley
**Acq Method**	Auto MSMS_1x50_5-95_90sec.m	**Acquired Time**	7/21/2022 4:25:01 PM (UTC-05:00)
**IRM Calibration Status**	Success	**DA Method**	Default Report AMM_210224.m
**Comment**	0.1 µL Injection		

**Sample Group**		**Info.**	
**Molecular Formula**	C14H9BrF2N2O	**Stream Name**	LC 1
**Acquisition Time (Local)**	7/21/2022 4:25:01 PM (UTC-05:00)	**Acquisition SW Version**	6200 series TOF/6500 series Q-TOF 10.1 (48.0)
**QTOF Driver Version**	10.01.00	**QTOF Firmware Version**	10.811
**DDE Mode**	2	**Tune Mass Range Max.**	3200

Compound Table

Compound Label	RT	Mass$_{m/z - addt}$	Abund	Name	Formula	Mass$_{TGT}$	Diff (ppm)	Hits (DB)
Cpd 1: JLE-20-78; C14 H9 Br F2 N2 O; 1.229	1.229	337.9868	252421	JLE-20-78	C14 H9 Br F2 N2 O	337.9866	0.61	1

Compound Label	Name	*m/z* Observed	RT	Algorithm
Cpd 1: JLE-20-78; C14 H9 Br F2 N2 O; 1.229	JLE-20-78	338.9942	1.229	Find by Formula

Calc m/z	Ion
338.9939	(M+H)+

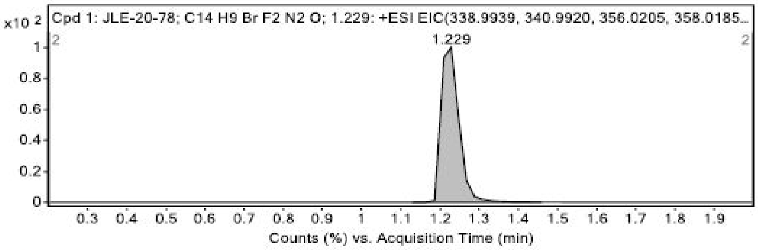

MS Zoomed Spectrum

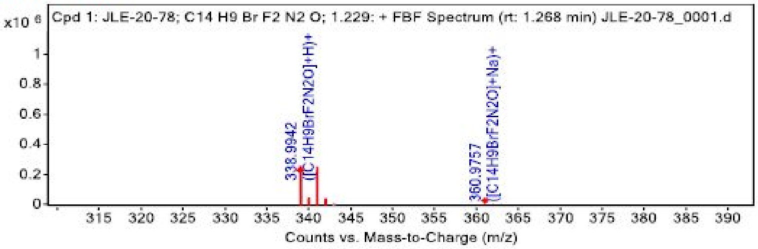

MS Spectrum Peak List

m/z	z	Abund	Formula	Ion
338.9942	1	252421.19	C14H9BrF2N2O	(M+H)+
339.9969	1	36513.58	C14H9BrF2N2O	(M+H)+
340.9922	1	233811.97	C14H9BrF2N2O	(M+H)+
341.9951	1	36413.84	C14H9BrF2N2O	(M+H)+
360.9757	1	1506.48	C14H9BrF2N2O	(M+Na)+
361.9811	1	337.2	C14H9BrF2N2O	(M+Na)+
362.9725	1	953.08	C14H9BrF2N2O	(M+Na)+

MS Zoomed Spectrum

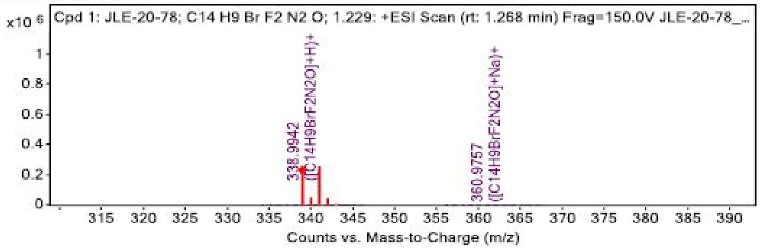

MS Spectrum Peak List

m/z	*Calc m/z*	Diff(ppm)	z	Abund	Formula	Ion
338.9942	338.9939	0.84	1	252421.19	C14H9BrF2N2O	(M+H)+
339.9969	339.997	-0.26	1	36513.58	C14H9BrF2N2O	(M+H)+
340.9922	340.992	0.55	1	233811.97	C14H9BrF2N2O	(M+H)+
341.9951	341.995	0.26	1	36413.84	C14H9BrF2N2O	(M+H)+
360.9757	360.9759	-0.4	1	1506.48	C14H9BrF2N2O	(M+Na)+
361.9811	361.9789	6.09	1	337.2	C14H9BrF2N2O	(M+Na)+
362.9725	362.9739	-3.92	1	953.08	C14H9BrF2N2O	(M+Na)+

MSMS Spectrum

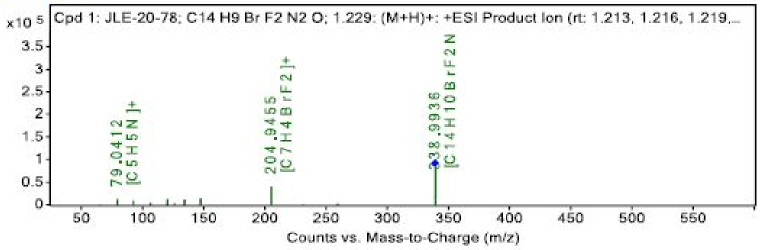

MSMS Spectrum

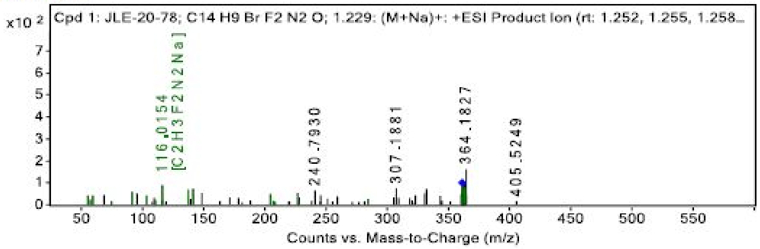

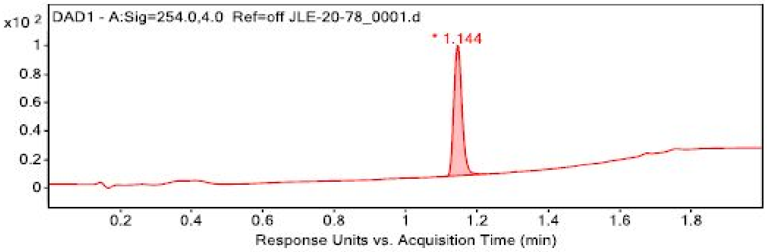

User Chromatogram Peak List

RT	Height	Normalized Height	Height %	Area	Area %	Area Sum %	Symmetry	Width
1.144	55.27	91.25	100	85.84	100	100	1.48	0.12

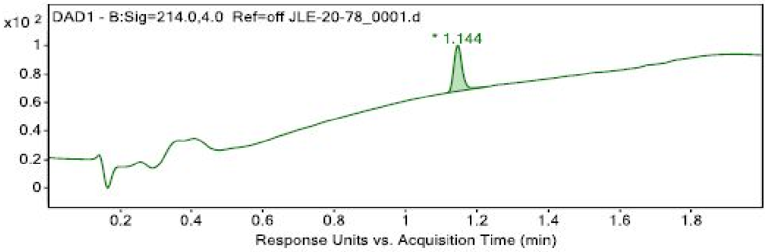

User Chromatogram Peak List

RT	Height	Normalized Height	Height %	Area	Area %	Area Sum %	Symmetry	Width
1.144	105.73	32.05	100	166.06	100	100	1.75	0.103

--- End Of Report ---

Preparation of 6-(2,6-difluoro-4-(4,4,5,5-tetramethyl-1,3,2-dioxaborolan-2-yl)benzyl)-6,7-dihydro-5*H*-pyrrolo[3,4-*b*]pyridin-5-one (15, JLE-20-97)

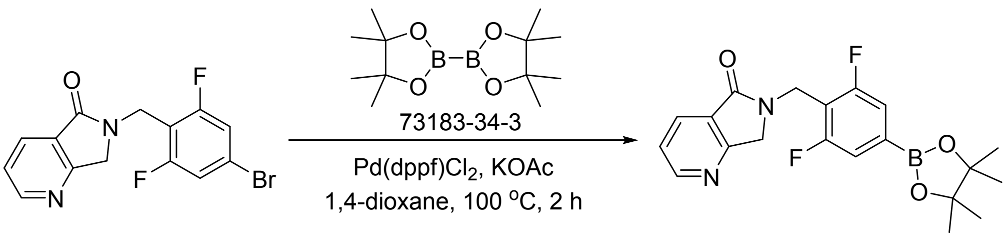

14, JLE-20-78 **15, JLE-20-97**

In a 1L RBF, 6-(4-bromo-2,6-difluorobenzyl)-6,7-dihydro-5*H*-pyrrolo[3,4-*b*]pyridin-5-one (**14**, **JLE-20-78**, 6.44 g, 19.0 mmol, 1.0 eq.), potassium acetate (5.60 g, 57.0 mmol, 3.0 eq.), [1,1'-bis(diphenylphosphino)ferrocene]dichloropalladium(II) complex with dichloromethane (1.39 g, 1.9 mmol, 0.1 eq.) and bis(pinocolato)diboron (7.24 g, 28.5 mmol, 1.5 eq.) were combined. Anhydrous 1,4-dioxane (190 mL, 0.2 M) was added. The reaction mixture was evacuated and purged with nitrogen (3x) and allowed to stir at 100 °C. After 2 h, the reaction mixture was filtered through a pad of Celite® which was rinsed thoroughly with EtOAc. Filtrate was concentrated under reduced pressure to provide the title compound **15** as a brown solid (13.16 g) which was carried forward without futher purification. ES-MS $[M+H]^+$ = 387.0 and $[\text{boronic acid}]^+$ = 304.9.

WCNDD CS Acquity LCMS 4

Julie_Engers

Sample ID:JLE-20-97-RXN Description: Vial:1:33

Date:10-Feb-2022 Time:14:31:43

Method:D:\WCNDD.PRO\OA_Methods\90-Second.olp

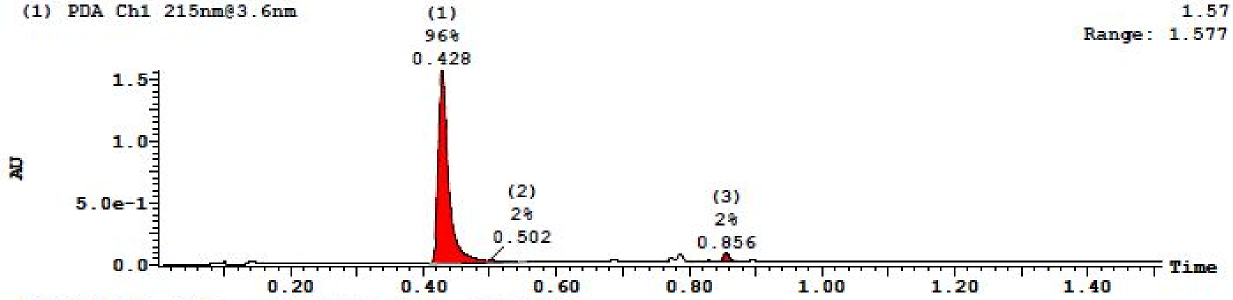

Peak Number	Time	Area %Total	BPM	Mass Found
1	0.428	95.96	305	Not Found
2	0.502	1.98	293	Not Found
3	0.856	2.06	587	Not Found

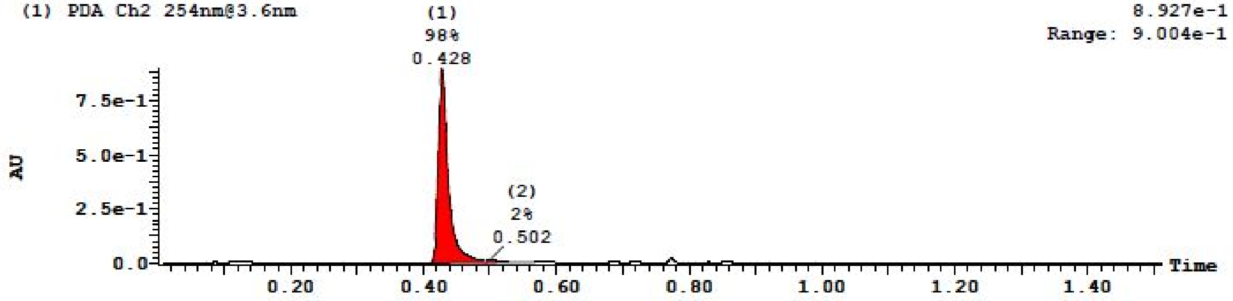

Peak Number	Time	Area %Total	BPM	Mass Found
1	0.428	97.62	305	Not Found
2	0.502	2.38	293	Not Found

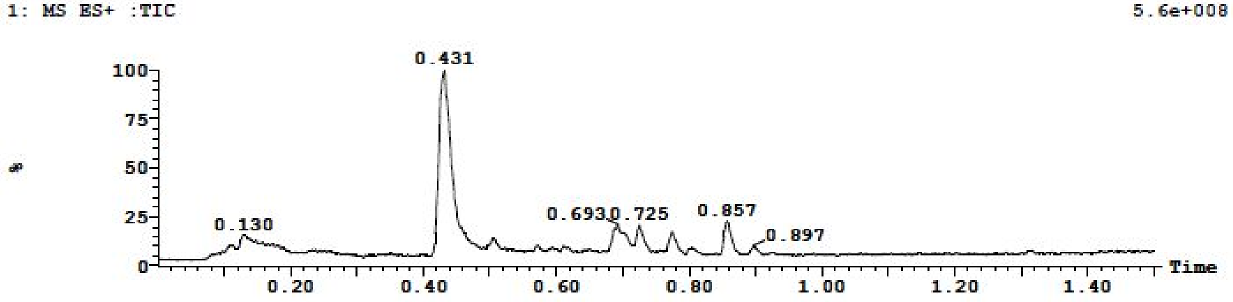

Julie_Engers

Sample ID:JLE-20-97-RXN Description: Vial:1:33
Date:10-Feb-2022 Time:14:31:43
Method:D:\WCNDD.PRO\OA_Methods\90-Second.olp

Peak ID	Time
1	0.428

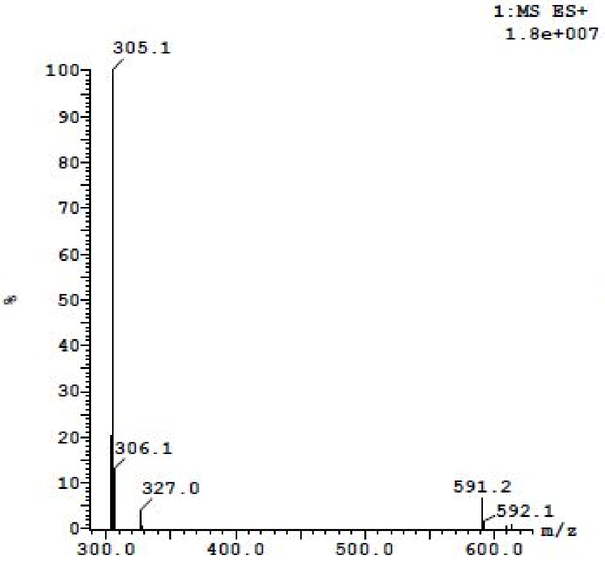

Peak ID	Time
2	0.502

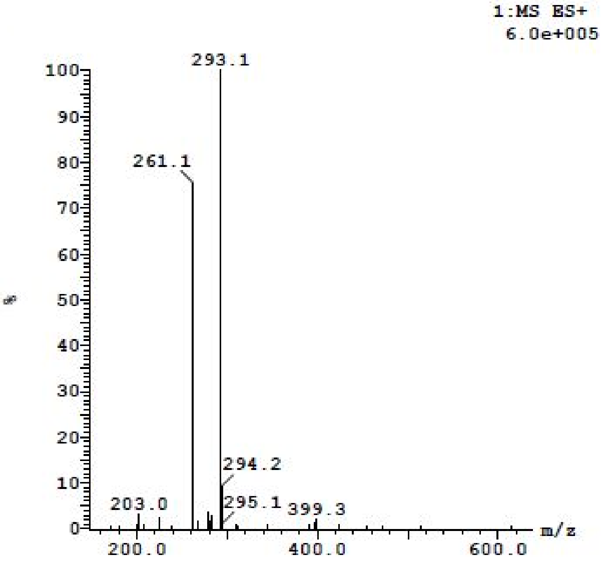

Peak ID	Time
3	0.856

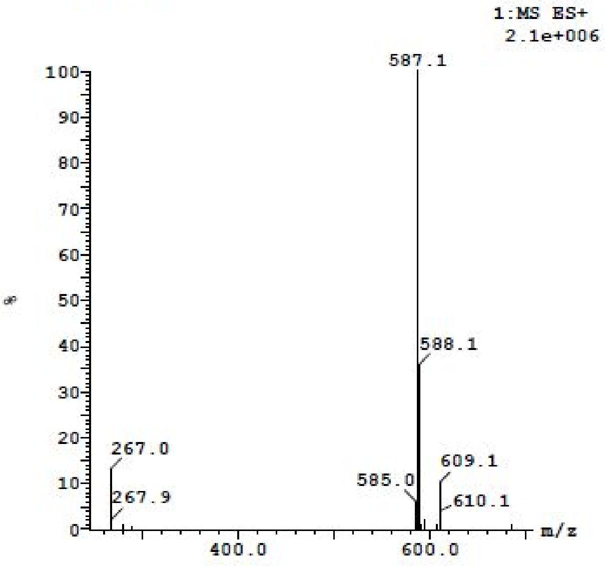

WCNDD CS Acquity LCMS 5 Page 1
Julie_Engers
Sample ID:JLE-20-97-FINAL Description: Vial:2:45
Date:11-Feb-2022 Time:07:33:16
Method:D:\WCNDD.PRO\OA_Methods\90-Second.olp

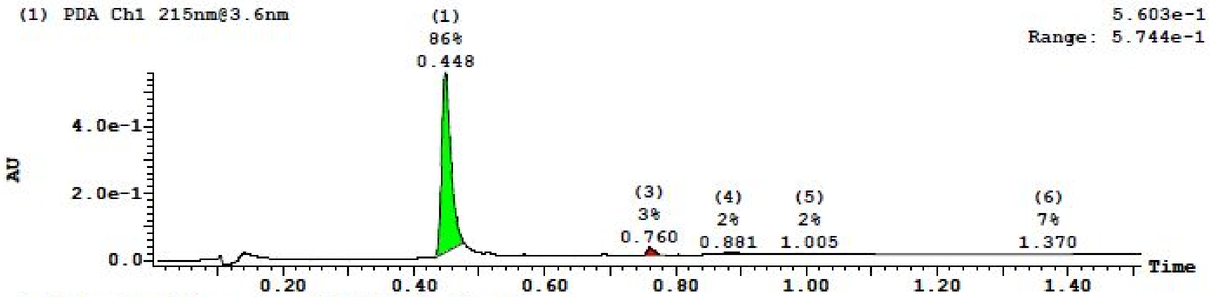

Peak Number	Time	Area %Total	BPM	Mass Found
1	0.448	85.84	305	304.0000
3	0.760	2.89	387	Not Found
4	0.881	2.31	587	Not Found
5	1.005	2.44	603	304.0000
6	1.370	6.52	553	Not Found

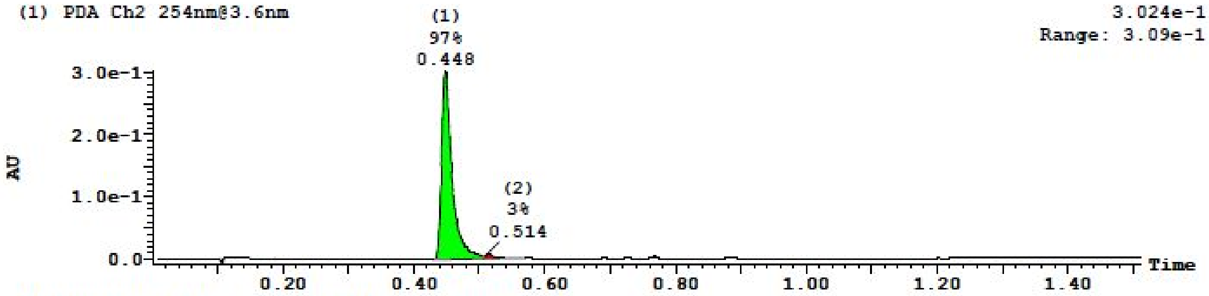

Peak Number	Time	Area %Total	BPM	Mass Found
1	0.448	97.23	305	304.0000
2	0.514	2.77	261	Not Found

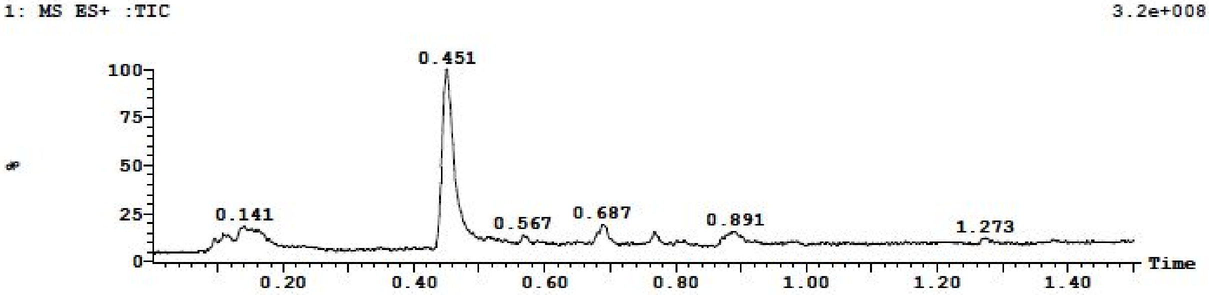

Sample ID:JLE-20-97-FINAL
Description:
Vial:2:45
Date:11-Feb-2022
Time:07:33:16
Method:D:\WCNDD.PRO\OA_Methods\90-Second.olp

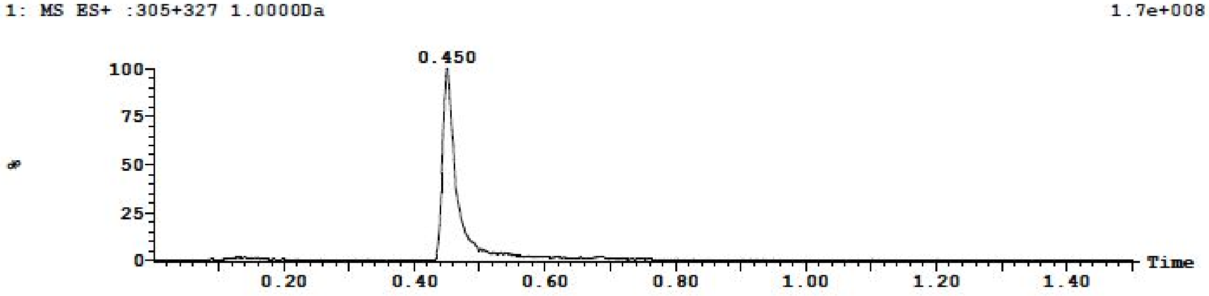

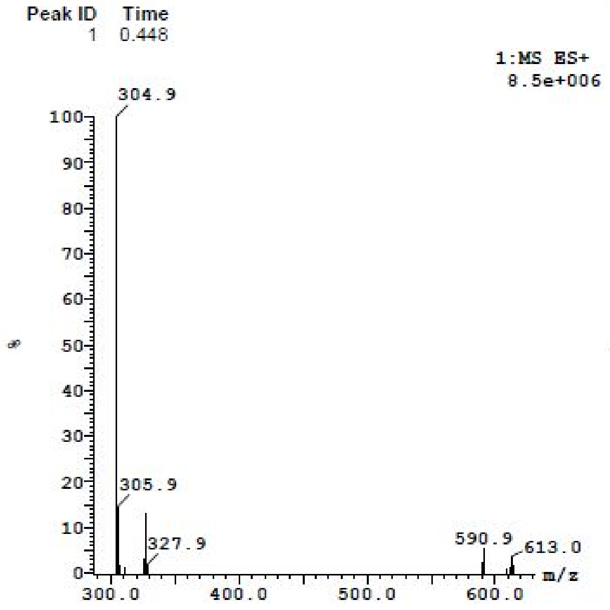

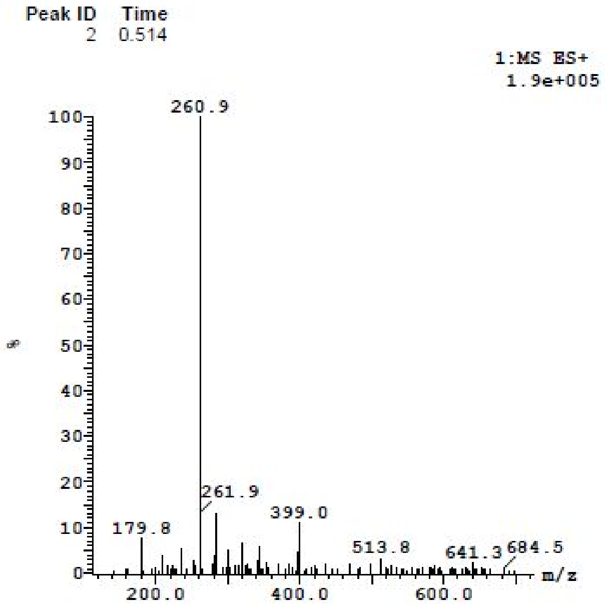

Julie_Engers

Sample ID:JLE-20-97-FINAL Description: Vial:2:45
Date:11-Feb-2022 Time:07:33:16
Method:D:\WCNDD.PRO\OA_Methods\90-Second.olp

Peak ID 3 **Time** 0.760

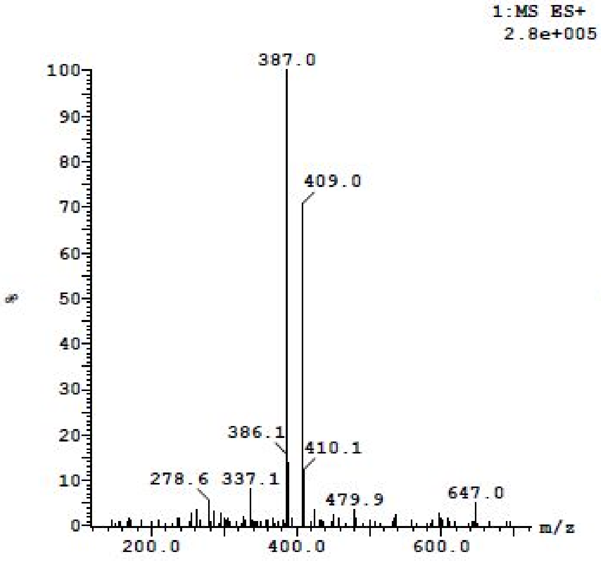

Peak ID 4 **Time** 0.881

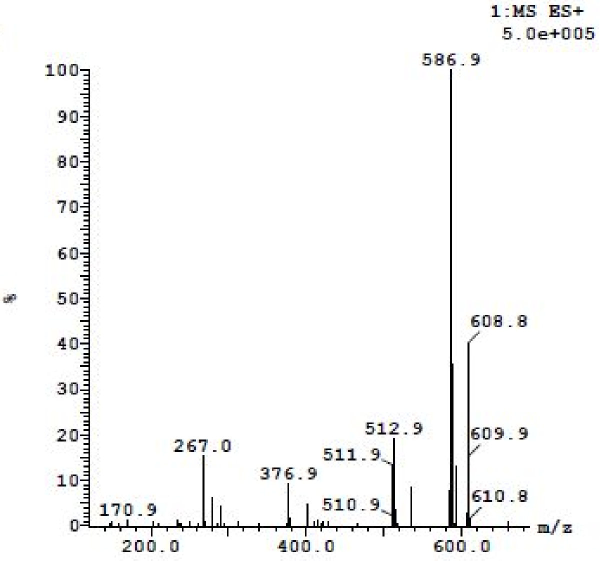

Peak ID 5 **Time** 1.005

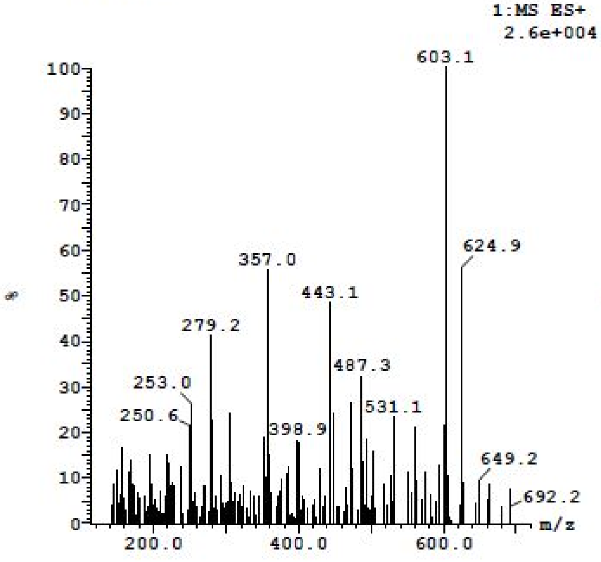

Peak ID 6 **Time** 1.370

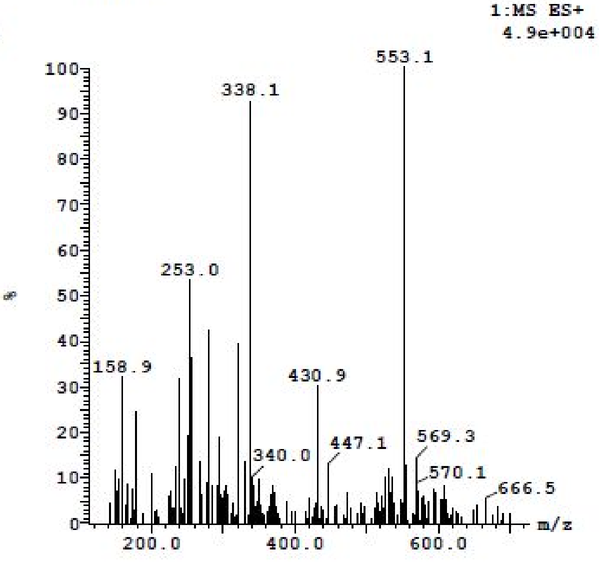

Preparation of 6-(2,6-difluoro-4-(7-(2-hydroxypropan-2-yl)-2-methyl-2*H*-indazol-4-yl)benzyl)-6,7-dihydro-5*H*-pyrrolo[3,4-*b*]pyridin-5-one (7, JLE-20-98 or VU6052254-05)

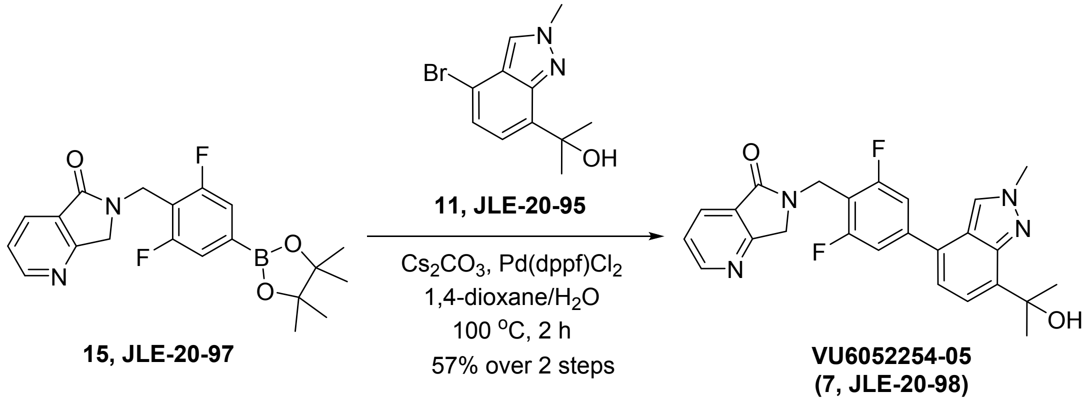

In 500 mL RBF, 2-(4-bromo-2-methyl-2*H*-indazol-7-yl)propan-2-ol (**11**, **JLE-20-95**, 3.85 g, 14.3 mmol, 1.0 eq.), [1,1'-bis(diphenylphosphino)ferrocene]dichloropalladium(II), complex with dichloromethane (1.05 g, 1.43 mmol, 0.1 eq.), and cesium carbonate (14.07 g, 42.9 mmol, 3.0 eq.) were combined. A solution of 6-(2,6-difluoro-4-(4,4,5,5-tetramethyl-1,3,2-dioxaborolan-2-yl)benzyl)-6,7-dihydro-5*H*-pyrrolo[3,4-*b*]pyridin-5-one (**15**, **JLE-20-97**, 11.95 g – 56 %wt, 17.2 mmol, 1.2 eq.) in anhydrous 1,4-dioxane (119.2 mL) was added followed by H_2O (23.8 mL). The reaction mixture was evacuated and purged with N_2 (3x) and allowed to stir at 100 °C. After 2 h, the reaction mixture was diluted with EtOAc (500 mL). The organic layer was filtered through a pad of Celite which was rinsed thoroughly with EtOAc/DCM. The filtrate was concentrated under reduced pressure. The crude material was purified using normal phase chromatography on silica gel (Teledyne ISCO, 330G RediSep Rf columns, solid loading, 0-100% EtOAc/DCM) to afford a light tan powder. The material was re-purified using normal phase chromatography on silica gel (Teledyne ISCO, 120G RediSep Rf column, solid loading, 0-10% MeOH/DCM). After concentration, the material was sonicated with ice cold MeOH (~10-15 mL). The precipitate was collected using vacuum filtration and washed with ice cold MeOH to provide the title compound **7** as a white crystalline powder (2.79 g). The filtrate was concentrated and repeated the purification procedure above to obtain a 2nd batch of material (0.86 g) – yielded a total of 3.65 g (57%). ^1H NMR (400 MHz, DMSO) δ 8.76 (dd, *J* = 4.9, 1.6 Hz, 1H), 8.59 (s, 1H), 8.12 (dd, *J* = 7.7, 1.6 Hz, 1H), 7.53 (dd, *J* = 7.7, 4.9 Hz, 1H), 7.49 – 7.42 (m, 3H), 7.22 (d, *J* = 7.3 Hz, 1H), 5.16 (s, 1H), 4.91 (s, 2H), 4.50 (s, 2H), 4.19 (s, 3H), 1.69 (s, 6H); ^{13}C NMR (101 MHz, DMSO) δ 165.41, 162.48 (d, $J_{C\text{-}F}$ = 9.3 Hz), 162.17, 160.01 (d, $J_{C\text{-}F}$ = 9.2 Hz), 152.62, 145.90, 142.45 (t, *J* = 10.2 Hz), 139.32, 131.47, 129.01 (t, *J* = 2.4 Hz), 125.30, 124.22, 120.64, 120.38, 120.24, 110.89, 110.81, 110.63, 110.60, 71.09, 50.77, 40.17, 33.41 (d, *J* = 3.4 Hz), 30.34 (2C); ES-MS $[M-H_2O]^+$ = 431.4; HRMS (ESI/Q-TOF) m/z: $[M+H]^+$ Calcd for $C_{25}H_{22}F_2N_4O_2$ 449.1784, Found 449.1785.

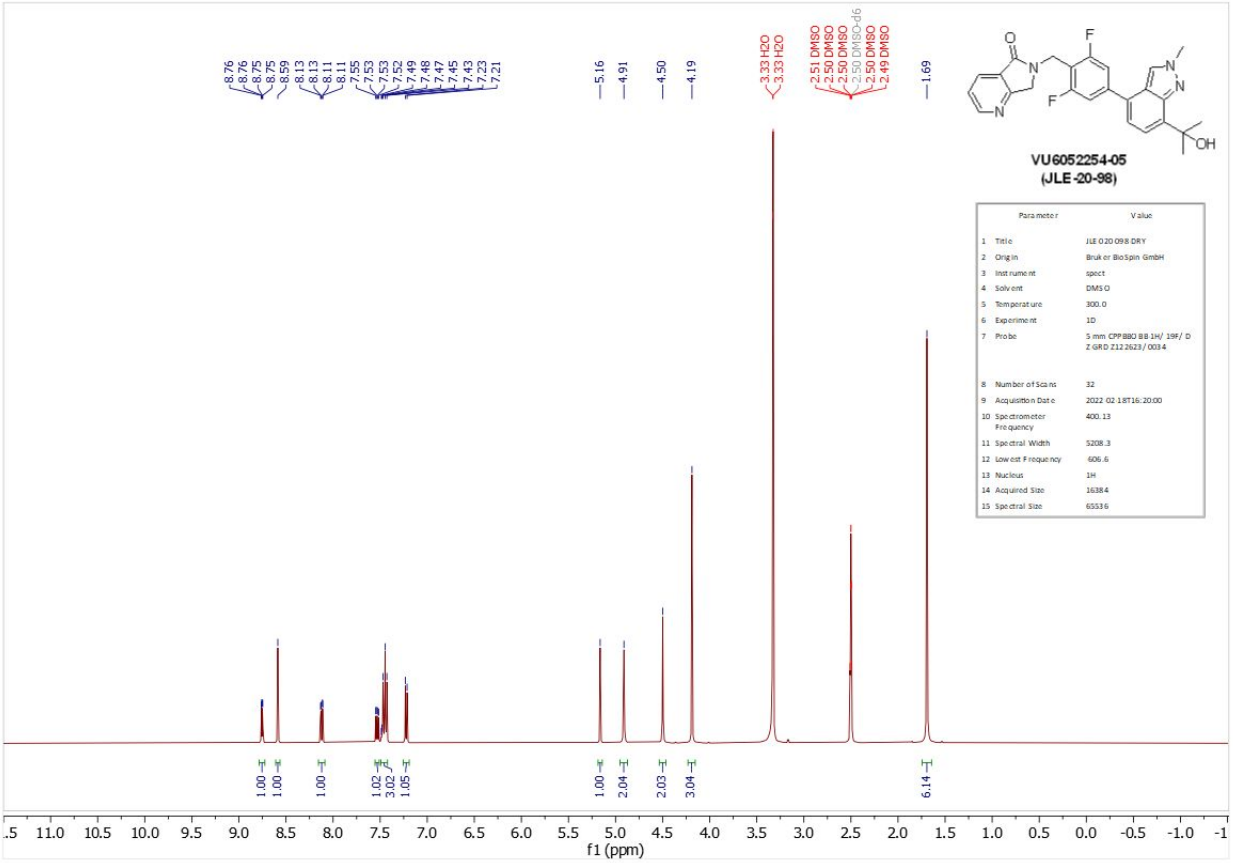

^{1}H NMR (400 MHz, DMSO) δ 8.76 (dd, *J* = 4.9, 1.6 Hz, 1H), 8.59 (s, 1H), 8.12 (dd, *J* = 7.7, 1.6 Hz, 1H), 7.53 (dd, *J* = 7.7, 4.9 Hz, 1H), 7.49 – 7.42 (m, 3H), 7.22 (d, *J* = 7.3 Hz, 1H), 5.16 (s, 1H), 4.91 (s, 2H), 4.50 (s, 2H), 4.19 (s, 3H), 1.69 (s, 6H).

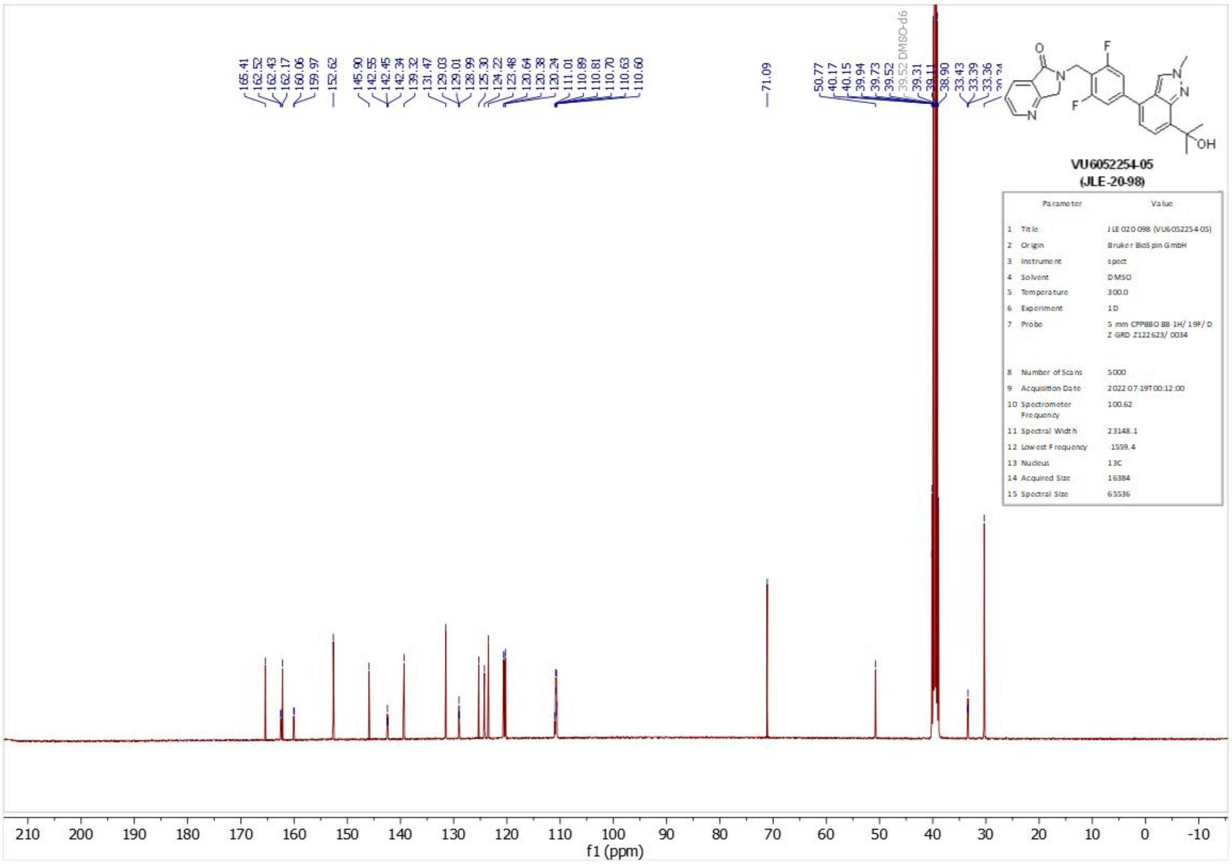

^{13}C NMR (101 MHz, DMSO) δ 165.41, 162.48 (d, $J_{C\text{-}F}$ = 9.3 Hz), 162.17, 160.01 (d, $J_{C\text{-}F}$ = 9.2 Hz), 152.62, 145.90, 142.45 (t, J = 10.2 Hz), 139.32, 131.47, 129.01 (t, J = 2.4 Hz), 125.30, 124.22, 120.64, 120.38, 120.24, 110.89, 110.81, 110.63, 110.60, 71.09, 50.77, 40.17, 33.41 (d, J = 3.4 Hz), 30.34 (2C)

WCNDD CS Acquity LCMS 5
Julie_Engers
Sample ID:JLE-20-98-RXN-100C-2H Description: Vial:1:30
Date:11-Feb-2022 Time:10:49:28
Method:D:\WCNDD.PRO\OA_Methods\90-Second.olp

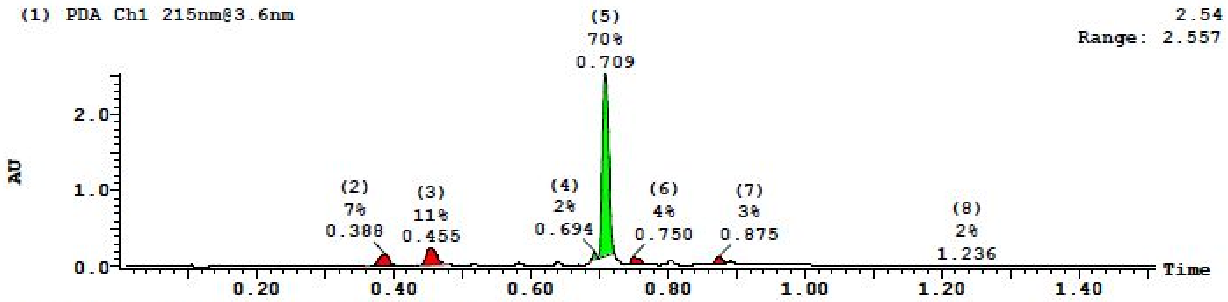

Peak Number	Time	Area %Total	BPM	Mass Found
1	0.307	1.80	217	Not Found
2	0.388	7.31	173	Not Found
3	0.455	11.06	305	Not Found
4	0.694	1.54	299	430.0000
5	0.709	69.67	431	430.0000
6	0.750	3.54	337	Not Found
7	0.875	3.44	587	Not Found
8	1.236	1.63	279	Not Found

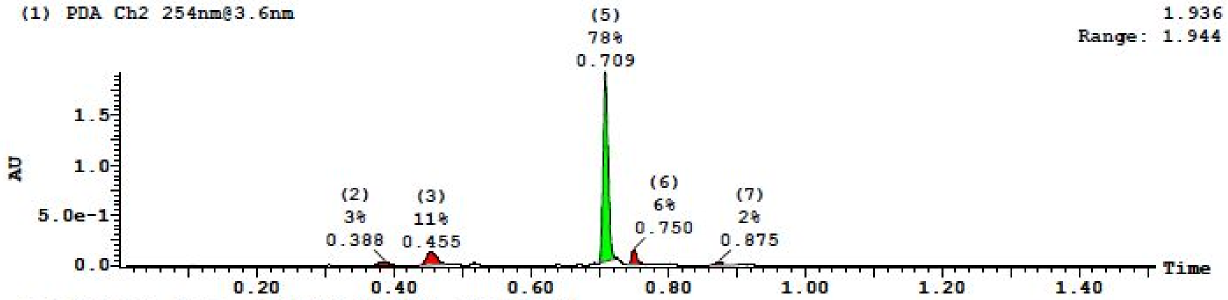

Peak Number	Time	Area %Total	BPM	Mass Found
2	0.388	2.65	173	Not Found
3	0.455	10.74	305	Not Found
5	0.709	78.45	431	430.0000
6	0.750	6.16	337	Not Found
7	0.875	1.99	587	Not Found

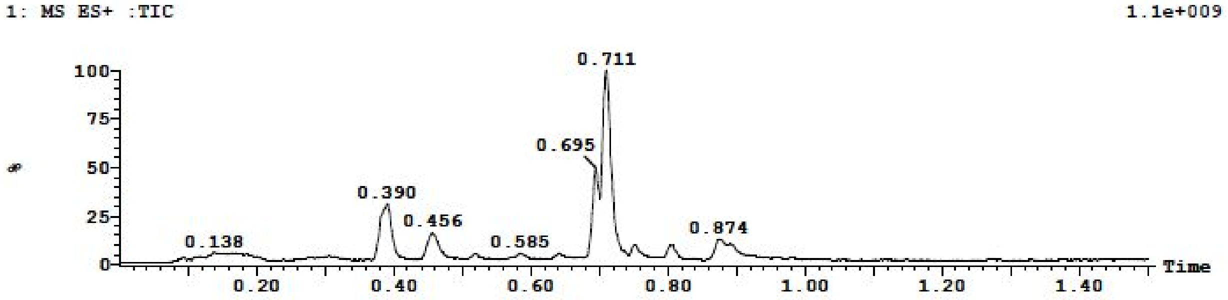

WCNDD CS Acquity LCMS 5
Julie_Engers
Sample ID:JLE-20-98-RXN-100C-2H Description: Vial:1:30
Date:11-Feb-2022 Time:10:49:28
Method:D:\WCNDD.PRO\OA_Methods\90-Second.olp

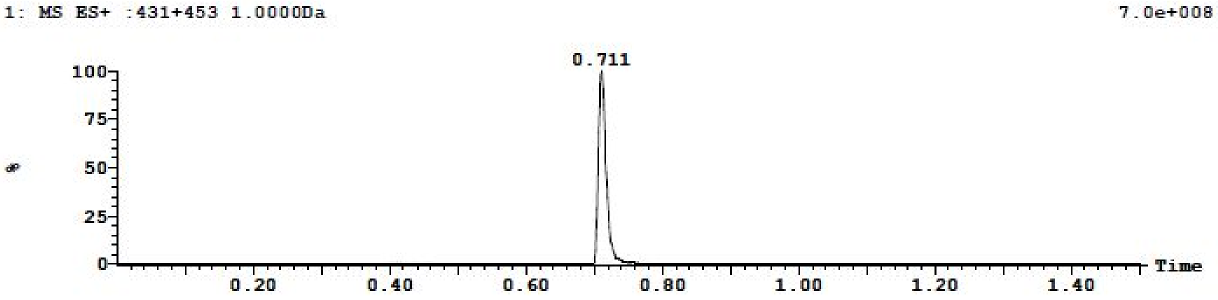

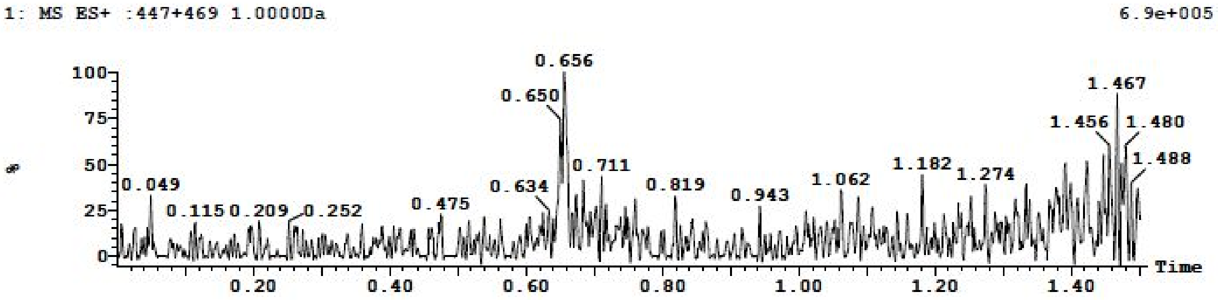

Peak ID 1 Time 0.307

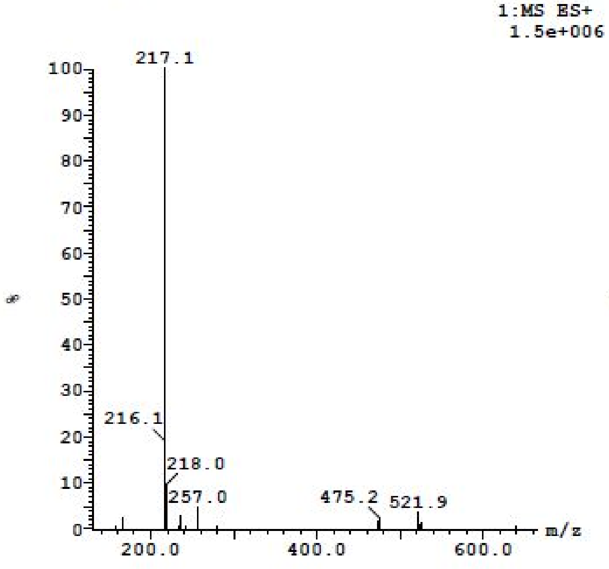

Peak ID 2 Time 0.388

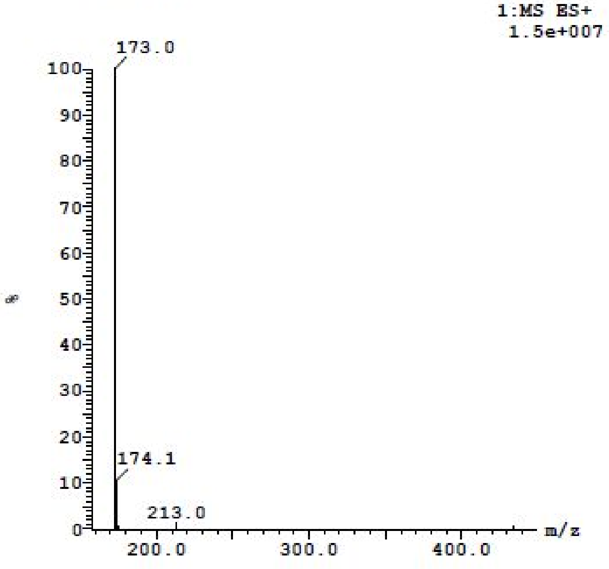

WCNDD CS Acquity LCMS 5
Julie_Engers
Sample ID:JLE-20-98-RXN-100C-2H Description: Vial:1:30
Date:11-Feb-2022 Time:10:49:28
Method:D:\WCNDD.PRO\OA_Methods\90-Second.olp

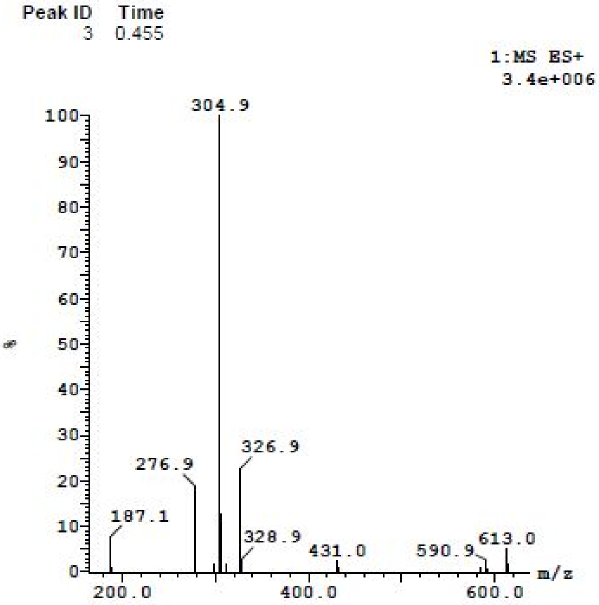

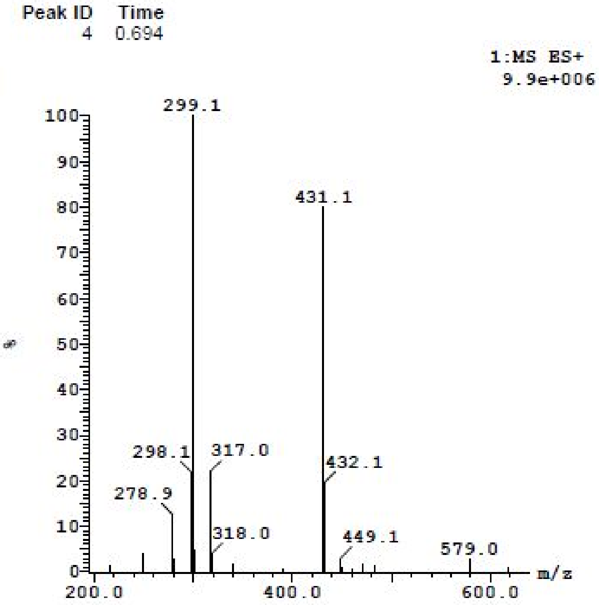

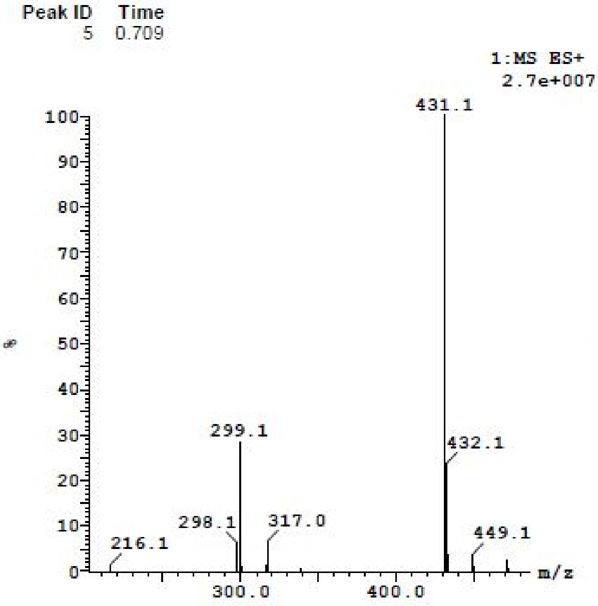

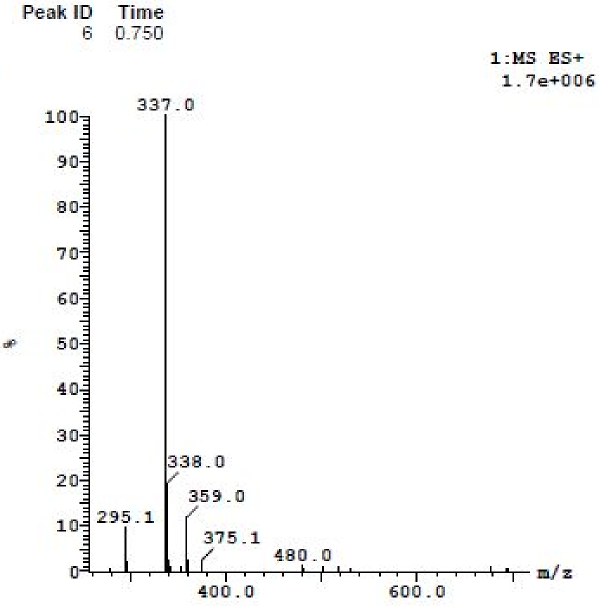

Julie_Engers

Sample ID:JLE-20-98-RXN-100C-2H Description: Vial:1:30
Date:11-Feb-2022 Time:10:49:28
Method:D:\WCNDD.PRO\OA_Methods\90-Second.olp

Peak ID	Time
7	0.875

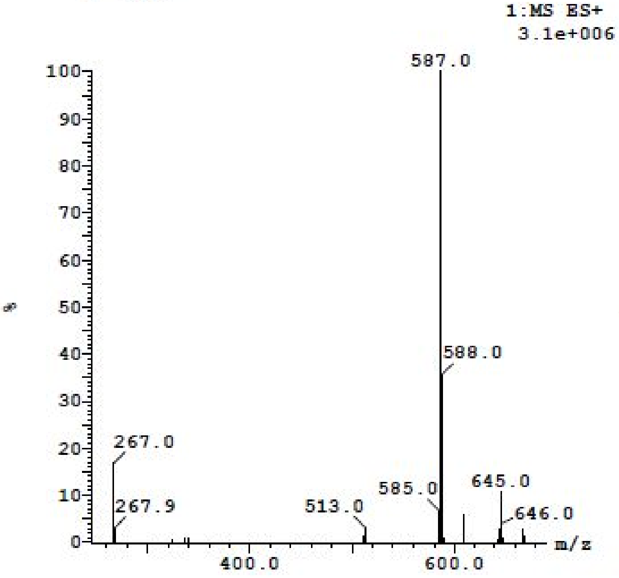

Peak ID	Time
8	1.236

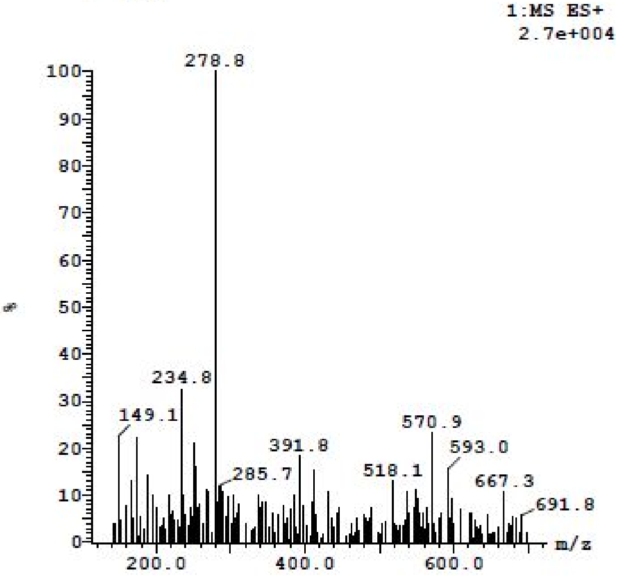

WCNDD CS Acquity LCMS 3

Julie_Engers

Page 1

Sample ID:JLE-20-98-FINAL-COMBINED Description: Vial:2:28

Date:21-Feb-2022 Time:13:31:30

Method:D:\WCNDD.PRO\OA_Methods\90-Second.olp

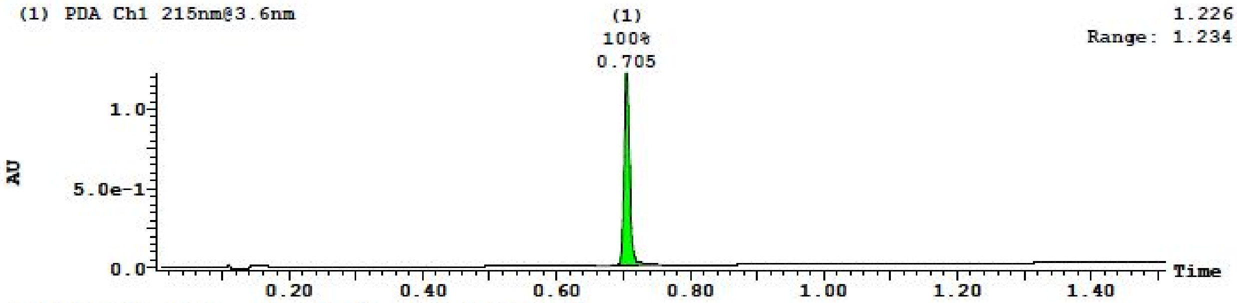

Peak Number	Time	Area %Total	BPM	Mass Found
1	0.705	100.00	431	430.0000

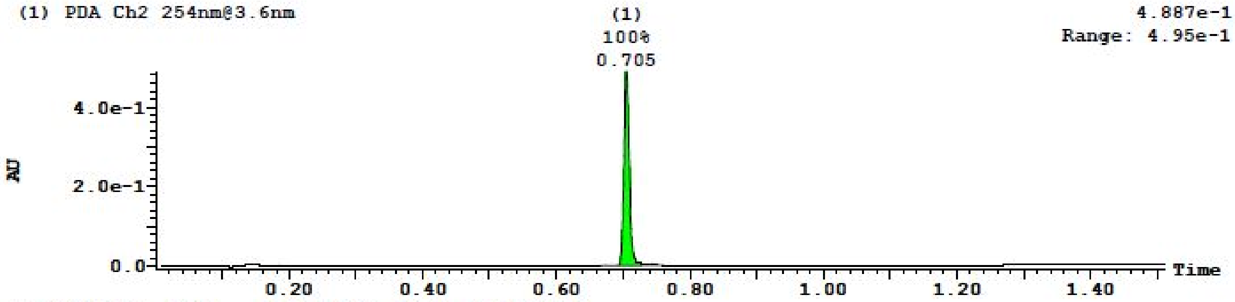

Peak Number	Time	Area %Total	BPM	Mass Found
1	0.705	100.00	431	430.0000

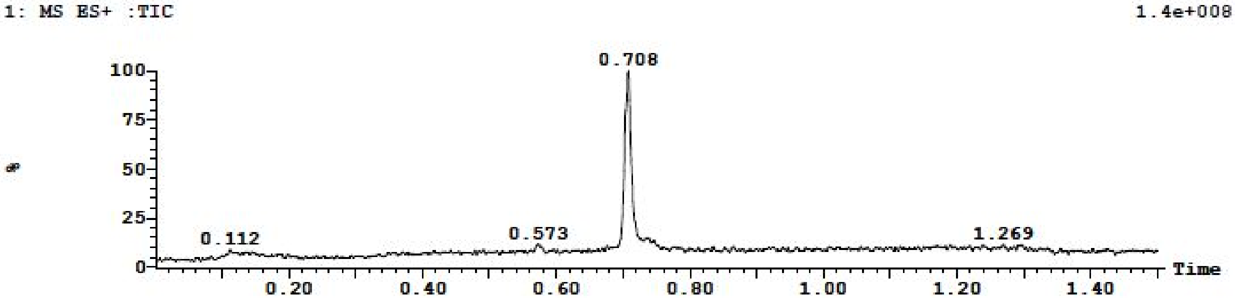

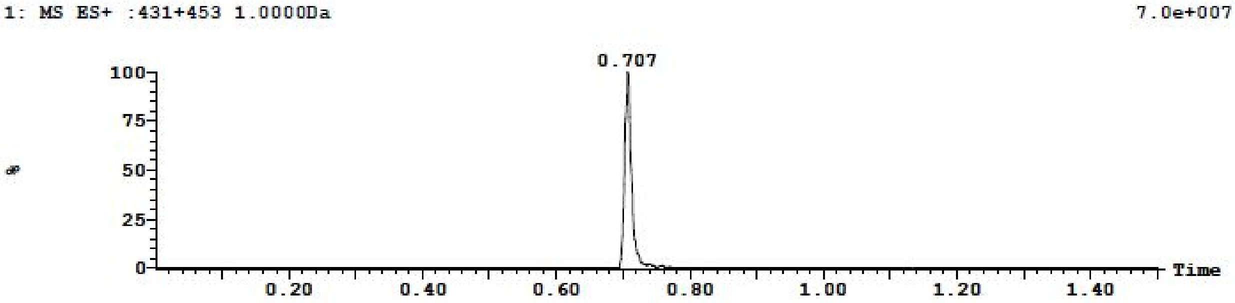

WCNDD CS Acquity LCMS 3
Julie_Engers
Sample ID:JLE-20-98-FINAL-COMBINED Description: Vial:2:28
Date:21-Feb-2022 Time:13:31:30
Method:D:\WCNDD.PRO\OA_Methods\90-Second.olp

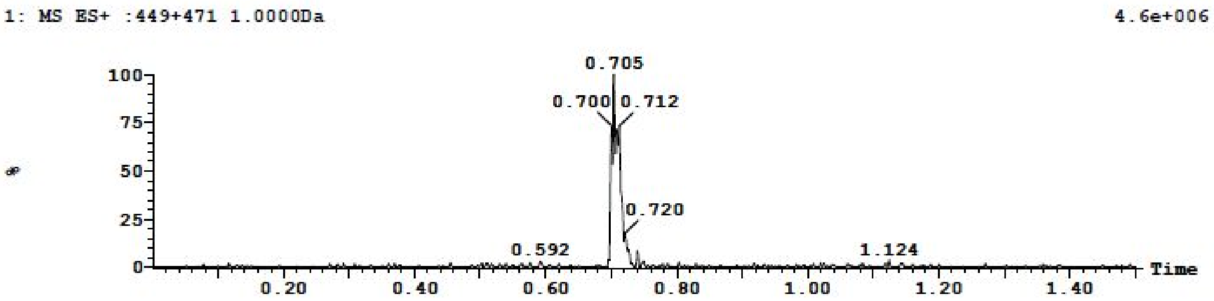

Peak ID	Time
1	0.705

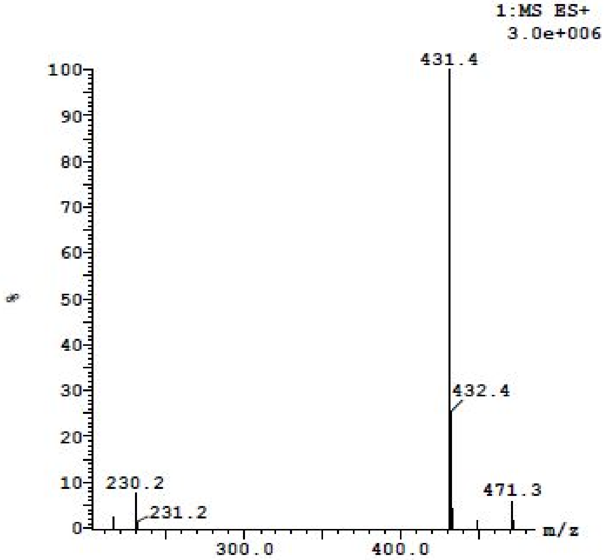

HRMS/Accurate Mass Report

WARREN CENTER FOR NEUROSCIENCE DRUG DISCOVERY at Vanderbilt University

**Data File**	VU6052254-05_0001.d	**Sample Name**	VU6052254-05
**Sample Type**	Sample	**Position**	P1-E3
**Instrument Name**	Q-TOF	**User Name**	Christopher Presley
**Acq Method**	Auto MSMS_1x50_5-95_90sec.m	**Acquired Time**	7/21/2022 4:53:14 PM (UTC-05:00)
**IRM Calibration Status**	Success	**DA Method**	Default Report AMM_210224.m
**Comment**	0.1 µL Injection, JLE-20-98		

**Sample Group**		**Info.**	
**Molecular Formula**	C25H22F2N4O2	**Stream Name**	LC 1
**Acquisition Time (Local)**	7/21/2022 4:53:14 PM (UTC-05:00)	**Acquisition SW Version**	6200 series TOF/6500 series Q-TOF 10.1 (48.0)
**QTOF Driver Version**	10.01.00	**QTOF Firmware Version**	10.811
**DDE Mode**	2	**Tune Mass Range Max.**	3200

Compound Table

Compound Label	RT	Mass$_{m/z\ -\ addt}$	Abund	Name	Formula	Mass$_{TGT}$	Diff (ppm)	Hits (DB)
Cpd 2: VU6052254-05; C25 H22 F2 N4 O2; 1.259	1.259	448.1712	251459	VU6052254-05	C25 H22 F2 N4 O2	448.1711	0.19	1

Compound Label	Name	*m/z* Observed	RT	Algorithm
Cpd 2: VU6052254-05; C25 H22 F2 N4 O2; 1.259	VU6052254-05	449.1785	1.259	Find by Formula

Calc m/z	Ion
449.1784	(M+H)+

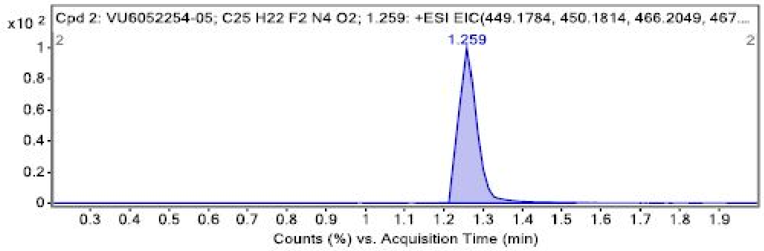

MS Zoomed Spectrum

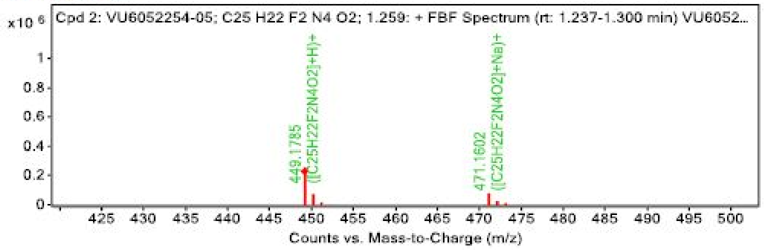

MS Spectrum Peak List

m/z	z	Abund	Formula	Ion
449.1785	1	251458.63	C25H22F2N4O2	(M+H)+
450.1817	1	72239.55	C25H22F2N4O2	(M+H)+
451.1844	1	11166.57	C25H22F2N4O2	(M+H)+
452.1883	1	1243.9	C25H22F2N4O2	(M+H)+
471.1602	1	76784.27	C25H22F2N4O2	(M+Na)+
472.1631	1	20678.66	C25H22F2N4O2	(M+Na)+
473.1662	1	3345.65	C25H22F2N4O2	(M+Na)+

MS Zoomed Spectrum

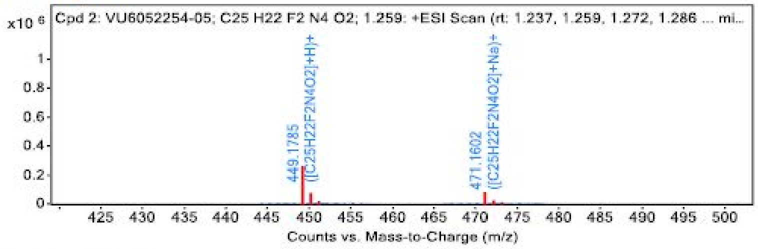

MS Spectrum Peak List

m/z	*Calc m/z*	Diff(ppm)	z	Abund	Formula	Ion
449.1785	449.1784	0.24	1	251458.63	C25H22F2N4O2	(M+H)+
450.1817	450.1814	0.59	1	72239.55	C25H22F2N4O2	(M+H)+
451.1844	451.1843	0.12	1	11166.57	C25H22F2N4O2	(M+H)+
452.1883	452.1871	2.87	1	1243.9	C25H22F2N4O2	(M+H)+
471.1602	471.1603	-0.2	1	76784.27	C25H22F2N4O2	(M+Na)+
472.1631	472.1634	-0.48	1	20678.66	C25H22F2N4O2	(M+Na)+
473.1662	473.1662	-0.15	1	3345.65	C25H22F2N4O2	(M+Na)+

MSMS Spectrum

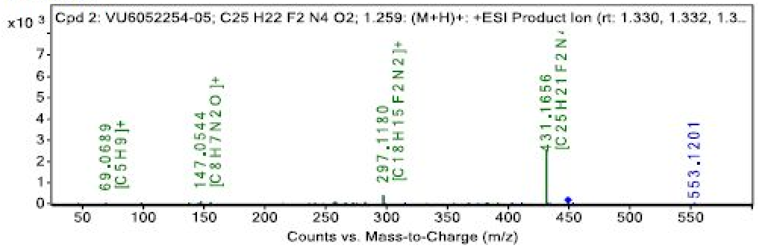

Supplemental Figures

Run	Compounds	% Baseline	PPR (%)	Mouse PAM EC50	[LTD]/ mouse EC50	Human PAM EC50	10,000/ human EC50
#4	ACP-2254, 10 µM	**86%**	**112%**	**80**	**125**	59	169

Figure S1. Rat LTD study with VU6052254/ACP-2254 (7).

Predicted Parameter	Method and Rationale	Value
V_{ss} (L/kg +/- S.D.)	Geo mean of 8 methods	1.16 +/- 0.32
Oral F (%)	Mean of observed %F in preclinical species (rat, dog, cyno)	79
CL_p (mL/min/kg +/- S.D.)	Geo mean of 6 methods	2.6 +/- 0.66
$t_{1/2}$ (hrs)	$t_{1/2} = \ln2 * V_{dss}/CL$	5.1
Dose (mg)*	$Dose = (C_{min,ss}*V_{dss}*(k_a-k_e))/(F*k_a*((e^{-k_e\tau})/(1- e^{-k_e\tau}))-(e^{-k_a\tau}/(1- e^{-k_a\tau})))$	**46 mg QD**
V_{ss} (L/kg +/- S.D.)	4 lowest values	0.91 +/- 0.15
$t_{1/2}$ (hrs)	$t_{1/2} = \ln2 * V_{dss}/CL$	4.0
Dose (mg)*	$Dose = (C_{min,ss}*V_{dss}*(k_a-k_e))/(F*k_a*((e^{-k_e\tau})/(1- e^{-k_e\tau}))-(e^{-k_a\tau}/(1- e^{-k_a\tau})))$	**84 mg QD**
V_{ss} (L/kg +/- S.D.)	4 highest values	1.41 +/- 0.21
$t_{1/2}$ (hrs)	$t_{1/2} = \ln2 * V_{dss}/CL$	6.3
Dose (mg)*	$Dose = (C_{min,ss}*V_{dss}*(k_a-k_e))/(F*k_a*((e^{-k_e\tau})/(1- e^{-k_e\tau}))-(e^{-k_a\tau}/(1- e^{-k_a\tau})))$	**32 mg QD**

Figure S2. Human PK and human dose projection summary for VU6052254 (7).

7. RESULTS

7.1. ADME-Tox: Genetic Toxicity

7.1.1. Test Compound Results

Compound I.D.	Client Compound I.D.	Test Concentration	%Control 1st	%Control 2nd	%Control 3rd	Mean %Control	Cytotoxicity (% of control)
Bacterial cytotoxicity (TA98 - S9)							
100060195-1	VU6052591-01	6.0E-07 M	105.2	120.1	131.5	**118.9**	119
100060195-1	VU6052591-01	1.2E-06 M	102.9	116.6	118.9	**112.8**	113
100060195-1	VU6052591-01	2.5E-06 M	101.8	105.2	115.5	**107.5**	107
100060195-1	VU6052591-01	5.0E-06 M	115.5	108.6	104.0	**109.4**	109
100060195-1	VU6052591-01	1.0E-05 M	104.0	107.5	105.2	**105.6**	106
100060195-1	VU6052591-01	2.5E-05 M	112.1	99.5	110.9	**107.5**	107
100060195-1	VU6052591-01	5.0E-05 M	101.8	101.8	105.2	**102.9**	103
100060195-1	VU6052591-01	1.0E-04 M	105.2	102.9	99.5	**102.5**	103
100060195-2	VU6052254-03	6.0E-07 M	97.2	110.9	104.0	**104.0**	104
100060195-2	VU6052254-03	1.2E-06 M	107.5	115.5	127.0	**116.6**	117
100060195-2	VU6052254-03	2.5E-06 M	108.6	120.1	107.5	**112.1**	112
100060195-2	VU6052254-03	5.0E-06 M	110.9	118.9	116.6	**115.5**	115
100060195-2	VU6052254-03	1.0E-05 M	105.2	117.8	112.1	**111.7**	112
100060195-2	VU6052254-03	2.5E-05 M	99.5	105.2	106.3	**103.7**	104
100060195-2	VU6052254-03	5.0E-05 M	89.2	96.0	98.3	**94.5**	94
100060195-2	VU6052254-03	1.0E-04 M	96.0	91.4	101.8	**96.4**	96
Bacterial cytotoxicity (TA100 - S9)							
100060195-1	VU6052591-01	6.0E-07 M	105.2	133.7	135.1	**124.7**	125
100060195-1	VU6052591-01	1.2E-06 M	105.2	118.0	123.7	**115.6**	116
100060195-1	VU6052591-01	2.5E-06 M	106.6	119.4	122.3	**116.1**	116
100060195-1	VU6052591-01	5.0E-06 M	106.6	118.0	115.2	**113.3**	113
100060195-1	VU6052591-01	1.0E-05 M	96.6	105.2	109.4	**103.7**	104
100060195-1	VU6052591-01	2.5E-05 M	109.4	105.2	105.2	**106.6**	107
100060195-1	VU6052591-01	5.0E-05 M	105.2	118.0	118.0	**113.7**	114
100060195-1	VU6052591-01	1.0E-04 M	128.0	119.4	140.8	**129.4**	129
100060195-2	VU6052254-03	6.0E-07 M	112.3	135.1	112.3	**119.9**	120
100060195-2	VU6052254-03	1.2E-06 M	112.3	123.7	120.9	**119.0**	119
100060195-2	VU6052254-03	2.5E-06 M	90.9	116.6	118.0	**108.5**	108
100060195-2	VU6052254-03	5.0E-06 M	106.6	109.4	89.5	**101.8**	102
100060195-2	VU6052254-03	1.0E-05 M	99.5	93.7	109.4	**100.9**	101
100060195-2	VU6052254-03	2.5E-05 M	95.2	88.0	99.5	**94.2**	94
100060195-2	VU6052254-03	5.0E-05 M	89.5	95.2	103.7	**96.1**	96
100060195-2	VU6052254-03	1.0E-04 M	96.6	100.9	99.5	**99.0**	99
Bacterial cytotoxicity (TA1535 - S9)							
100060195-1	VU6052591-01	6.0E-07 M	115.4	105.5	115.4	**112.1**	112
100060195-1	VU6052591-01	1.2E-06 M	106.9	99.8	108.3	**105.0**	105
100060195-1	VU6052591-01	2.5E-06 M	106.9	101.2	108.3	**105.5**	105
100060195-1	VU6052591-01	5.0E-06 M	104.1	97.0	98.4	**99.8**	100
100060195-1	VU6052591-01	1.0E-05 M	101.2	106.9	101.2	**103.1**	103
100060195-1	VU6052591-01	2.5E-05 M	109.7	101.2	108.3	**106.4**	106
100060195-1	VU6052591-01	5.0E-05 M	114.0	105.5	115.4	**111.6**	112
100060195-1	VU6052591-01	1.0E-04 M	112.6	105.5	114.0	**110.7**	111

Figure S3. Genetic toxicity test (AMES) with VU6052254 (7).

Pharmacokinetic Profile of VU6052254 in Male Sprague Dawley Rats after Single Oral Doses of 10, 30 and 100 mg/kg

Compound	PO Dose Group	Nominal PO Dosage	Animal	Actual PO Dosage (mg/kg)	No pts used for $t_{1/2}$	$t_{1/2}$ (h)	t_{max} (h)	C_{max} (ng/mL)	AUC_{last} (h*ng/mL)	AUC_{Inf} (h*ng/mL)	AUC Extr (%)	MRT_{Inf} (h)	AUC/D (h*kg*ng/mL/mg)
VU6052254	1	10 mg/kg	Rat 1M	10.05	5	4.38	2.00	1620	11200	11200	0	5.13	1110
			Rat 2M	10.10	4	4.39	4.00	3490	28500	28600	0	5.31	2830
			Rat 3M	9.98	4	4.26	4.00	2070	22000	22000	0	5.97	2200
					n	3	3	3	3	3	3	3	3
					**Mean**	**4.34**	**3.33**	**2390**	**20600**	**20600**	**0**	**5.47**	**2050**
					SD	0.0723	1.15	976	8740	8780	0	0.442	870
					%CV	1.7	34.7	40.8	42.4	42.6	NA	8.1	42.4

Last time point for AUC_{last}: 48 h for all animals

T_{last} for all animals was greater than 3-fold the estimated $t_{1/2}$. Extrapolated PK parameters are reported.

NA: Not applicable.

Compound	PO Dose Group	Nominal PO Dosage	Animal	Actual PO Dosage (mg/kg)	No pts used for $t_{1/2}$	$t_{1/2}$ (h)	t_{max} (h)	C_{max} (ng/mL)	AUC_{last} (h*ng/mL)	AUC_{Inf} (h*ng/mL)	AUC Extr (%)	MRT_{Inf} (h)	AUC/D (h*kg*ng/mL/mg)
VU6052254	2	30 mg/kg	Rat 4M	31.99	4	3.91	4.00	13700	113000	113000	0	5.48	3520
			Rat 5M	30.10	5	3.91	2.00	11700	104000	104000	0	5.38	3470
			Rat 6M	30.66	3	4.68	6.00	11100	128000	129000	0	6.90	4190
					n	3	3	3	3	3	3	3	3
					**Mean**	**4.17**	**4.00**	**12200**	**115000**	**115000**	**0**	**5.92**	**3730**
					SD	0.445	2.00	1360	12100	12700	0	0.850	402
					%CV	10.7	50.0	11.1	10.5	11.0	NA	14.4	10.8

Last time point for AUC_{last}: 48 h for all animals

T_{last} for all animals was greater than 3-fold the estimated $t_{1/2}$. Extrapolated PK parameters are reported.

NA: Not applicable.

Compound	PO Dose Group	Nominal PO Dosage	Animal	Actual PO Dosage (mg/kg)	No pts used for $t_{1/2}$	$t_{1/2}$ (h)	t_{max} (h)	C_{max} (ng/mL)	AUC_{last} (h*ng/mL)	AUC_{Inf} (h*ng/mL)	AUC Extr (%)	MRT_{Inf} (h)	AUC/D (h*kg*ng/mL/mg)
VU6052254	3	100 mg/kg	Rat 7M	98.58	4	4.33	4.00	25800	344000	344000	0	8.53	3490
			Rat 8M	101.95	4	5.32	4.00	26000	321000	322000	0	10.0	3150
			Rat 9M	99.54	4	4.40	4.00	22400	283000	283000	0	9.39	2840
					n	3	3	3	3	3	3	3	3
					**Mean**	**4.68**	**4.00**	**24700**	**316000**	**316000**	**0**	**9.31**	**3160**
					SD	0.552	0.00	2020	30800	30900	0	0.74	325
					%CV	11.8	0.0	8.2	9.7	9.8	NA	7.9	10.3

Last time point for AUC_{last}: 48 h for all animals

T_{last} for all animals was greater than 3-fold the estimated $t_{1/2}$. Extrapolated PK parameters are reported.

NA: Not applicable.

Figure S4. Rat dose escalation study with VU6052254 (7).
